# Supplementary material for: Outcomes tested in non-pharmacological interventions in mild cognitive impairment and mild dementia: a scoping review
Source: BMJ Open. 2020 Apr 20;10(4):e035980. doi: 10.1136/bmjopen-2019-035980 (PMC7204934; doi:10.1136/bmjopen-2019-035980)
Supplement: Supplementary data [file bmjopen-2019-035980supp001.pdf]

Supplementary Table 1. Search Strategy for OVID

	Search term		Search term continued
**1**	Early dementia	**39**	self help group
**2**	Mild dementia	**40**	psychotherapy
**3**	mild alzheimer*	**41**	CBT
**4**	early alzheimer*	**42**	Cognitive behavio?ral therap*
**5**	cognitive impairment	**43**	Cognitive behavioural therap*
**6**	age related cognitive impairment	**44**	Talking therap*
**7**	Mild cognitive impairment	**45**	Individual therap*
**8**	MCI	**46**	Peer support
**9**	mild neurocognitive disorder	**47**	Counselling
**10**	**1 OR 2 OR 3 OR 4 OR 5 OR 6 OR 7 OR 8 OR 9**	**48**	Communication
**11**	cognitive training	**49**	acupuncture therap*
**12**	brain training	**50**	acupuncture
**13**	memory training	**51**	acupuncture points
**14**	Behavio?r therap*	**52**	Transcranial Magnetic Stimulation
**15**	Behavio?r modification	**53**	TMS
**16**	pleasant activit*	**54**	Relaxation therap*
**17**	Cognitive stimulation therapy	**55**	Therap* relaxation
**18**	CST	**56**	Relaxation techniques
**19**	Transcutaneous Electrical Nerve Stimulation	**57**	Early intervention
**20**	TENS	**58**	Alternative therap*
**21**	Exercise	**59**	**11 OR 12 OR 13 OR 14 OR 15 OR 16 OR 17 OR 18 OR 19 OR 20 OR 21 OR 22 OR 23 OR 24 OR 25 OR 26 OR 27 OR 28 OR 29 OR 30 OR 31 OR 32 OR 33 OR 34 OR 35 OR 36 OR 37 OR 38 OR 39 OR 40 OR 41 OR 42 OR 43 OR 44 OR 45 OR 46 OR 47 OR 48 OR 49 OR 50 OR 51 OR 52 OR 53 OR 54 OR 55 OR 56 OR 57 OR 58 OR 59**
**22**	exercise therap*	**60**	randomized controlled trial
**23**	Walking	**61**	randomised controlled trial

**24**	music therap*	**62**	RCT
**15**	reminiscence therap*	**63**	Clinical Trial
**26**	massage therap*	**64**	intervention
**27**	therap* touch	**65**	**60 OR 61 OR 62 OR 63 OR 64 OR 65**
**28**	recreation therap*	**66**	early dementia
**29**	light therap*	**67**	mild dementia
**30**	therap* light	**68**	mild alzheimer*
**31**	sensory stimulation	**69**	early alzheimer*
**32**	multisensory stimulation	**70**	cognitive impairment
**33**	complementary therap*	**71**	age related cognitive impairment
**34**	aromatherapy	**72**	Mild cognitive impairment
**35**	support group	**73**	MCI
**36**	therap* group	**74**	mild neurocognitive disorder
**37**	memory group	**75**	**66 OR 67 OR 68 OR 69 OR 70 OR 71 OR 72 OR 73 OR 74 OR 75**
**38**	self help	**76**	**10 AND 59 AND 75**
