# Supplementary material for: Outcomes tested in non-pharmacological interventions in mild cognitive impairment and mild dementia: a scoping review
Source: BMJ Open. 2020 Apr 20;10(4):e035980. doi: 10.1136/bmjopen-2019-035980 (PMC7204934; doi:10.1136/bmjopen-2019-035980)
Supplement: Author's manuscript [file bmjopen-2019-035980.draft_revisions.pdf]

BMJ Open

BMJ Open is committed to open peer review. As part of this commitment we make the peer review history of every article we publish publicly available.

When an article is published we post the peer reviewers' comments and the authors' responses online. We also post the versions of the paper that were used during peer review. These are the versions that the peer review comments apply to.

The versions of the paper that follow are the versions that were submitted during the peer review process. They are not the versions of record or the final published versions. They should not be cited or distributed as the published version of this manuscript.

BMJ Open is an open access journal and the full, final, typeset and author-corrected version of record of the manuscript is available on our site with no access controls, subscription charges or pay-per-view fees (http://bmjopen.bmj.com).

If you have any questions on BMJ Open's open peer review process please email info.bmjopen@bmj.com

Outcomes tested in non-pharmacological interventions in MCI and early dementia: a scoping review

Journal:	*BMJ Open*
Manuscript ID	bmjopen-2019-035980
Article Type:	Original research
Date Submitted by the Author:	25-Nov-2019
Complete List of Authors:	Couch, Elyse; King's College London, Health Service and Population Research<br>Lawrence, Vanessa; King's College London, Health Service and Population Research<br>Co, Melissa; King's College London, Health Service and Population Research<br>Prina, A.Matthew; King's College London, Health Service and Population Research
Keywords:	Dementia < NEUROLOGY, Old age psychiatry < PSYCHIATRY, STATISTICS & RESEARCH METHODS

SCHOLARONE™
Manuscripts

I, the Submitting Author has the right to grant and does grant on behalf of all authors of the Work (as defined in the below author licence), an exclusive licence and/or a non-exclusive licence for contributions from authors who are: i) UK Crown employees; ii) where BMJ has agreed a CC-BY licence shall apply, and/or iii) in accordance with the terms applicable for US Federal Government officers or employees acting as part of their official duties; on a worldwide, perpetual, irrevocable, royalty-free basis to BMJ Publishing Group Ltd ("BMJ") its licensees and where the relevant Journal is co-owned by BMJ to the co-owners of the Journal, to publish the Work in this journal and any other BMJ products and to exploit all rights, as set out in our licence.

The Submitting Author accepts and understands that any supply made under these terms is made by BMJ to the Submitting Author unless you are acting as an employee on behalf of your employer or a postgraduate student of an affiliated institution which is paying any applicable article publishing charge ("APC") for Open Access articles. Where the Submitting Author wishes to make the Work available on an Open Access basis (and intends to pay the relevant APC), the terms of reuse of such Open Access shall be governed by a Creative Commons licence – details of these licences and which Creative Commons licence will apply to this Work are set out in our licence referred to above.

Other than as permitted in any relevant BMJ Author's Self Archiving Policies, I confirm this Work has not been accepted for publication elsewhere, is not being considered for publication elsewhere and does not duplicate material already published. I confirm all authors consent to publication of this Work and authorise the granting of this licence.

For peer review only

Outcomes tested in non-pharmacological interventions in MCI and early dementia: a scoping review

Elyse Couch[1*], Vanessa Lawrence[1], Melissa Co[1], and A. Matthew Prina[1]

[a] Health Service and Population Research Department, Institute of Psychiatry, Psychology and Neuroscience, King's College London, UK

Elyse.Couch@kcl.ac.uk

Melissa.Co@kcl.ac.uk

Matthew.Prina@kcl.ac.uk

Vanessa.c.Lawrence@kcl.ac.uk

*Corresponding author information: Health Service and Population Research Department, Institute of Psychiatry, Psychology and Neuroscience, King's College London, UK SE5 8AF.

Email: Elyse.Couch@kcl.ac.uk

For peer review only

Abstract

Objectives: Non-pharmacological treatments are an important aspect of dementia care. A wide range of interventions have been trialled for the early stages of dementia and mild cognitive impairment. However, the variety of outcome measures used in these trials makes it difficult to make meaningful comparisons. The objective of this study is to map which outcome measures are used in trials of non-pharmacological treatments in MCI and early dementia.

Design: Scoping Review

Data Sources: EMBASE, Psych Info, Medline and the Cochrane Register of Controlled Trials were searched from inception until February 2018. An additional search was conducted in April 2019

Eligibility: We included RCTs testing non-pharmacological interventions for people diagnosed with MCI or early-stage dementia. Studies were restricted to full RCTs; observational, feasibility and pilot studies were not included.

Charting Methods: All outcome measures used by included studies were extracted and grouped thematically. Trends in the types of outcome measures used were explored by type of intervention and over time.

RESULTS: 92 studies were included in this review. We extracted 361 individual outcome measures, of which 78 (22%) were used more than once. Cognitive measures were the most frequently used, with the MMSE being the most popular.

Conclusions: Our findings highlight an inconsistency in the use of outcome measures. Cognition has been prioritised over other domains, despite previous research highlighting the importance of quality of life and caregiver measures. To ensure a robust, globally applicable evidence base, more research is needed to highlight which outcome measures should be used over others.

Protocol Registration**:** The protocol for this study was registered on PROSPERO (ID: CRD42018102649).

For peer review only - http://bmjopen.bmj.com/site/about/guidelines.xhtml

For peer review only

Word count: 3,519

Strengths and Limitations of this study:

- This scoping review has systematically mapped which outcome measures have been used by randomised controlled trials testing non-pharmacological treatments in early dementia and mild cognitive impairment.
- This is the first review to explore the broader trends in the use of outcome measures in this area of work.
- The papers included in this review were limited to full randomised controlled trials, other study designs may be using different types of outcome measures.
- Further research is needed to establish which measures should be used over others.

For peer review only

Introduction

Delivery of treatment in the early stages of dementia has been identified as a global priority [1 2]. Current pharmacological treatments for the cognitive symptoms of dementia have been found to have greater effect when delivered as early as possible [3] however, the benefits of delivering non-pharmacological treatments early are less well understood. Non-pharmacological treatments are an important clinical tool for managing dementia as they are more acceptable to some and less prone to side effects, making them a safe alternative to drug treatments [4] Those diagnosed earlier in the disease have more cognitive abilities available to engage with non-pharmacological treatments and bolster their own methods for coping with the disease [5]. Previous systematic reviews have found non-pharmacological treatments can improve outcomes; however, these reviews were restricted to a small number of outcome measures [6 7].

Mild cognitive impairment (MCI) has been identified as a potential prodrome for dementia, with approximately 10% of people with MCI converting to a diagnosis of dementia [8]. There is an interest in MCI, as a diagnosis of MCI can facilitate an early diagnosis of dementia and therefore earlier access to dementia services and treatment [9]. MCI is a potentially reversible condition, with many people with MCI reverting back to normal levels of cognition [9] therefore, it is important treatments are available. However, it is not clear which treatments can reverse MCI or prevent conversion to dementia [3]. No drug treatments for MCI have been found to be effective [10 11] and acetylcholinesterase inhibitors are not recommended however, there is some limited evidence that non-pharmacological interventions may be beneficial [3 12].

Randomised Controlled Trials (RCTs) testing non-pharmacological treatments in dementia and MCI are becoming more common. However, they are highly heterogeneous in terms of participants recruited, quality of the study and the types of interventions they are testing, making it difficult to establish the effectiveness of one treatment over another [6 12]. Compounding these issues is the inconsistent use of outcome measures in this area of work [9 13].

For peer review only - http://bmjopen.bmj.com/site/about/guidelines.xhtml

Systematic reviews have identified possible benefits of non-pharmacological treatment, yet meta-analyses are difficult to conduct due to the variation in outcome measures used by studies and typically yield small to moderate effect sizes [6 7]. It is possible that these small effect sizes are due to the selection of outcome measures which either lack sensitivity or the change following the intervention not being in the area covered by the outcome measure. It is important researchers are clear on which domains their interventions are targeting, and which measures are best able to capture this change [14]. As non-pharmacological treatments become more effective, there needs to be a more coherent use of outcome measure internationally to ensure a broad and robust evidence base [14].

In 2008, the INTERDEM group, a consortium of dementia researchers across Europe, did work to draw a consensus on which outcome measures should be used when evaluating non-pharmacological treatments. They recommended 22 measures across nine domains including quality of life, mood, global functioning, behaviour, daily living skills, caregiver mood, caregiver burden and staff morale [14]. This guidance does not explore outcomes by the stage of the disease. Furthermore, the outcome measures were selected based on their applicability to European research, it is important to have consistency in outcome measures globally.

It is not understood which outcome measures are currently being used in non-pharmacological treatments for early dementia and MCI. Scoping reviews present the opportunity to map the evidence on a topic [15], unlike a systematic review scoping reviews can be used to summarise the evidence in a heterogeneous body of literature. Therefore, the aim of this scoping review is to map which outcome measures are being used in RCTs for non-pharmacological treatments in MCI and early dementia.

Objectives

The specific objectives of this scoping review are to:

For peer review only - http://bmjopen.bmj.com/site/about/guidelines.xhtml

For peer review only

(1) Chart which outcomes measures have been used to assess the effectiveness of nonpharmacological treatments in early dementia and MCI
(2) Highlight which types of measures have been used most frequently
(3) Explore whether the outcome measures used differ depending on the type of intervention being tested

Methods

Protocol registration

The protocol for this review was developed following the guidelines set out by the Preferred Reporting Items for Systematic Reviews and Meta-Analysis Extension (PRISMA) statement [16] and the PRISMA guidelines for Scoping Reviews (PRISMA-ScR) [15]. The protocol was registered prospectively on PROSPERO (ID: CRD42018102649).

Eligibility criteria

We included RCTs testing non-pharmacological interventions for people diagnosed with MCI or early-stage dementia. Studies were restricted to full RCTs; observational, feasibility and pilot studies were not included.

Studies were included if they met the following criteria:

- Testing non-pharmacological interventions. Studies were not excluded if participants were also treated with acetylcholinesterase inhibitors
- Participants had a diagnosis of MCI or early-stage dementia, which either met standardised diagnostic criteria, such as the International Statistical Classification of Diseases (ICD-10) or The Diagnostic Statistical Manual of Mental Disorders (DSM); or was defined by a standardised clinical measure, such as the Mini-Mental State Exam (MMSE), the Clinical Dementia Rating (CDR) or the Global Deterioration Scale (GDS). Studies which include a mix of participants with early dementia and MCI were included, however, studies which included

For peer review only

For peer review only - http://bmjopen.bmj.com/site/about/guidelines.xhtml

healthy participants and participants with dementia at both at the later stages of the disease were excluded.

- The intervention was targeted for the person living with dementia or MCI. Dyadic interventions, interventions delivered to both the person living with dementia and their caregivers, were included, however, interventions delivered solely to caregivers or health care professionals were excluded.
- Participants were living in care homes or the community
- Written in English

Studies were excluded if:

- Only pharmacological interventions were tested
- The participants were diagnosed with vascular cognitive impairment or young onset dementia as they have a different trajectory of decline
- Participants were living in a psychiatric inpatient or acute hospital setting
- The intervention had the primary aim of treating depression
- The study tested palliative care interventions or advanced care planning
- The only outcome measure were economic outcomes, such as cost effectiveness etc.

Information sources and search strategy

To identify potentially relevant studies, we searched EMBASE, Psych Info, Medline and the Cochrane Register of Controlled Trials from inception until 22nd February 2018, an additional search was conducted on 2nd April 2019. See **Table A.1** for the final search strategy for MEDLINE, which was adapted for the other databases. The final search results were exported into EndNote where duplicates were removed.

Additional papers were identified by searching the references of included papers and other systematic reviews. Conference abstracts and publications were not included.

For peer review only - http://bmjopen.bmj.com/site/about/guidelines.xhtml

Selection of sources of evidence

Study selection was managed in Rayyan, where citations were screened against the inclusion and exclusion criteria. Rayyan is an online app for systematic reviews which allows researchers to create their own coding system for decision making [17]. References were first screened by title and abstract, followed by a full-text screening. A second reviewer (MC) screened 10% of the articles at each stage of the review. Disagreements were resolved by discussions with a third reviewer (MP).

A critical appraisal or assessment of the risk of bias is not necessary for a scoping review [15]. This scoping review is not aiming to critically appraise the cumulative literature of outcome measures for early non-pharmacological treatment in dementia, therefore we did not conduct a critical appraisal or risk of bias assessment for this review.

Data charting process and data items

Data from eligible studies were charted using a standardised extraction tool designed for this study. Items deemed most relevant to the review objectives were the diagnosis of the study participants, description of interventions being tested, the number of intervention groups, and outcome measures used with references.

Synthesis of results

The charted data were mapped to reflect the objectives of this review. Following data charting, outcome measures which used more than once across the included studies were grouped by domain, we grouped the interventions thematically by the type of intervention being tested.

We explored which types of outcome measures were used by intervention type, by tabulating the type of intervention against the domain of the outcome measure. We excluded interventions which were only used once from this summary. Results were presented in tables and summarised narratively.

Patient and Participant Involvement

For peer review only - http://bmjopen.bmj.com/site/about/guidelines.xhtml

The South London and Maudsley MALADY group, of current and former carers of people living with dementia, were consulted in the planning of this study.

Results

Included studies

After duplicates were removed, a total of 7,056 citations were screened for inclusion, 653 were screened at full text and 76 papers were initially identified. A top-up search in April 2019 identified 119 new citations, 18 were included making the total number of included studies 92, See **Figure 1**.

The studies included in this review are described in **Table 1**, including diagnosis of included participants, number of intervention groups, details on the interventions and comparisons tested and the number of outcomes measured used. The included studies were published between 2002 and 2019.

The majority of studies included in this review were conducted in the USA (n=11) followed by Hong Kong (n=10), Italy (n=10), mainland China (n=7), Japan (n=7), and South Korea (n=7). Studies were also conducted in: Argentina, Australia, Brazil, Canada, Czech Republic, Denmark, France, Finland, Germany, Greece, Hungary, Iran, Norway, Pakistan, Singapore, Spain, Taiwan, The Netherlands, Turkey, and the United Kingdom; these countries had fewer than 5 included studies.

Most studies only recruited participants with MCI (n=73), followed by early-stage dementia only (n=15), and six studies recruited both participants with MCI and early-stage dementia.

Results of induvial sources of evidence

We extracted 361 individual outcome measures from the included studies, of these 78 (22%) were used more than once. Out of the 78 measures used more than once, 70 (88%) were measures of participants living with dementia (PLWD), 6 measures were used in both the PLWD and their caregiver, 2 measures were only of the caregiver. The number of outcome measures used by each study ranged between one and 21 with an average of 6.85.

For peer review only - http://bmjopen.bmj.com/site/about/guidelines.xhtml
For peer review only

Types of non-pharmacological interventions

We grouped the interventions thematically by type. The most frequently tested type of intervention was cognitive training (n=37) followed by physical activity (n=25), combined physical activity and cognitive training (n=4), multicomponent psychosocial interventions (n=4) and support groups (n=3). Animal-assisted therapies, art-based therapies, case management, Chinese calligraphy, music-based interventions and reminiscence therapy were each tested in two studies.

A group weight loss programme, mindfulness, social activities, transcranial direct current stimulation (TDS), transcutaneous electrical nerve stimulation (TENs), and Transcranial magnetic stimulation (TMS) were each trialled once. These interventions were not included in the analysis of trends in outcome measures.

PLWD outcome measures

Table 2 presents the PLWD specific outcome measures grouped by domain. The most frequently measured domain in PLWD was cognition/memory, which was measured 219 times across the 93 included studies. The most frequent measure of cognition was the MMSE, which was measured 37 times. In addition to measures of memory performance knowledge of memory strategies was measured 3 times in PLWD.

The next most frequently measured domain in PLWD was behavioural and psychological symptoms of dementia (BPSD), within this depression was the most commonly measured BPSD. The Geriatric Depression Scale was the most used measure in this domain, followed by the Neuropsychiatric Inventory which examines a greater number of symptoms. Other BSPDs measured were apathy and agitation resulting from memory problems.

Quality of life and wellbeing were measured 15 times across the included study. Quality of life was measured 15 times using four different instruments, the most popular of which was Logsdon's Quality of Life in Alzheimer's disease scale which was used seven times.

For peer review only - http://bmjopen.bmj.com/site/about/guidelines.xhtml

Measures of everyday living, physical ability, biological outcomes and adherence to the intervention delivered in the study were measured less than 20 times across the included studies.

Caregiver measures

Eight interventions in this study were dyadic [18-25], all included outcome measures specific to the caregiver in addition to the PLWD. One study of an intervention solely delivered to the PLWD also included a caregiver specific measure [26].

Table 2 also presents the outcome measures administered to caregivers grouped by domain. The Center for Epidemiological Studies Depression Scale and the Zarit Caregiver Burden interview were the only measures which were administered solely to caregivers. The other caregiver measures were also administered to PLWD. The most frequently measured domain in caregivers was depression, followed by caregiver burden. General wellbeing, knowledge of memory strategies, quality of life and stress were each measured once.

Use of outcome measures over time

Randomised controlled trials of non-pharmacological treatments in early dementia and MCI have become more frequent over recent years. Almost half (48%) of studies included in this review were published between 2016 and 2018.

Figure 2 charts the use of outcome measures domains over time. As the number of studies in this area has increased over time, so too has the use of outcome measures in all domains. Cognition/memory has consistently been measured over other domains from the beginning of this sample. The only noticeable trend change is in measures of BPSD, which was generally in line with other domains until around 2012, where it overtakes other domains.

Nearly all studies in 2014 included a measure of everyday living, however, since then, the number of studies including these measures have declined. Where measures of everyday living are being used less, measures of BPSD are being used more.

For peer review only - http://bmjopen.bmj.com/site/about/guidelines.xhtml

For peer review only

Similarly, caregiver measures were consistently used until 2011, where in 2010 and 2011 all studies included a caregiver measure, however since then the use of such measures has declined.

Use of outcome measures by intervention

Table 3 presents diagnosis and type of intervention by the domains measured. Cognition/memory was the most measured domain across all diagnostic groups, followed by BPSD. The next most common domain measured for studies of people with dementia was caregiver specific measures, whereas in MCI it was physical performance.

Cognition/memory was measured in all types of intervention. BPSD was measured in all types of interventions except for combined cognitive and physical training interventions but was particularly favoured by studies testing cognitive training and psychosocial interventions. Quality of life was measured by studies of case management, cognitive training, psychosocial interventions, physical activity and support groups.

Caregiver measures were used in five types of interventions. Case management, cognitive training and psychosocial interventions; followed by arts-based therapy and support groups.

Discussion

In this study, we used a scoping review to map which outcome measures had been used in trials for non-pharmacological treatments of early dementia and MCI. We extracted 361 individual outcome measures used in 92 trials, only 22% of which were used more than once. We grouped the outcome measures which had been used more than once and examined differences in their use over time, by diagnostic group and by the type of intervention they were being used to evaluate. Measures of cognition and BPSDs were the most frequently used across all studies and types of intervention.

Perhaps unsurprisingly, measures of cognition or memory are the most prevalent across all diagnostic groups and types of intervention with the MMSE being the most frequently used outcome measure, despite the ADAS-cog having been validated as the gold-standard measure of cognition [14 27]

For peer review only - http://bmjopen.bmj.com/site/about/guidelines.xhtml

[28]. Measuring cognition is central to measuring the progression of dementia and is clinically and empirically useful outcome to measure in dementia research [28], however, in this review, we charted 40 different measures of cognition. This indicates that while cognition has been prioritised as an outcome in studies of non-pharmacological interventions, there is no consensus between researchers on which specific measures should be used. In addition to measures of cognitive performance, three studies have also measured participants knowledge or retention of memory strategies, indicating an interest in longer-term coping strategies for memory loss.

Measures of the BPSD have become more common over time, becoming in 2017 the most measured outcome after cognition. There is not much variety in the BPSDs which have been measured. Generally, depression was measured over other BPSDs. Other BPSDs such as agitation were measured less, perhaps because they are more associated with the later stages of the disease and depression is associated with the earlier stages [29].

Quality of life and wellbeing was not amongst the most measured domains. Four measures of quality of life were used 13 times across the included studies, all but one of these measures were dementia specific measures. It is surprising quality of life has not been measured more, as previous research has stated that in the absence of a cure, health care providers have a greater ability to improve quality of life than alter the progression of the disease [30]. Furthermore, in a priority setting exercise in people diagnosed with MCI and their caregivers, both people with MCI and caregivers rated quality of life of the patient as the most important outcome to measure, followed by caregiver quality of life/burden [31]. Indicating while quality of life has been identified as a priority by PLWD, MCI and their caregivers in previous research, the findings of this study shows this is not being translated into trials of non-pharmacological treatments for early dementia and MCI.

Likewise, caregiver measures had consistent low use across the studies included in this review. We charted eight caregiver measures which were used 11 times across the included studies. Caregiver measures were more commonly used in studies of PLWD, rather than MCI. Previous research has

For peer review only - http://bmjopen.bmj.com/site/about/guidelines.xhtml

highlighted the profound effect of the disease on their caregivers, with around half of caregivers experiencing high levels of burden [32]. However, a third of caregivers of people with MCI also report extreme levels of burden [33], yet the findings of this study show this is less investigated.

There was a lot of variability in the types of outcome measures being used to evaluate the different types of intervention. All studies measured cognition and all but one measured BPSD. Outcome measures should be selected depending on the domains the intervention is seeking to address [28]

In 2008, the INTERDEM group recommended 22 outcome measures for use across nine domains [14]. We found 11 of these 22 measures (50%) were used by the studies included in this review, one of the recommended domains (staff carer morale) was not applicable to the studies included in this review. All measures recommended for measuring patient mood, patient quality of life and patient quality of life were charted in this review. Only one of the recommended measures for the activities of daily living, caregiver mood, caregiver burden and caregiver quality of life domains were charted and no measures under the global measures domain were charted in this review. This indicates that there is some consistency between which measures are recommended and which measures are utilised, this is largely for patient measures and there is less consistency for caregiver measures.

Limitations

The findings of this review must be interpreted in the context of the study. To make this review feasible we only included full RCTs, other outcome measures may have been used in different types of studies. Furthermore, only outcome measures which were published could be included in this review. The studies included in this study were heterogeneous in terms of participants recruited, interventions tested, and outcome measures used, making it difficult to group them thematically. It is possible some nuance is lost in the exploration of broader themes. As with the nature of scoping reviews, we are only able to present which outcome measures have been used in previous research, we are unable to draw conclusions as to which outcome measures should be used over others.

For peer review only - http://bmjopen.bmj.com/site/about/guidelines.xhtml
For peer review only

Implications and recommendations for future research

The findings of this review indicate there is very little consistency in outcome measures used in RCTs for non-pharmacological interventions in MCI and early dementia, however we care not able to conclude which measures should be used over others. To create a strong, global evidence base for non-pharmacological treatments more research, with the involvement of PLWD and their carers, is needed to determine which measures preferable over a greater number of domains. Additionally, the prevalence of cognitive measures found in this study, suggests that researchers are including such measures because there is an expectation to do. Researchers should be clear on the theory behind how their intervention creates change and use the appropriate outcome measures.

Conclusions

In summary, this study has found RCTs for non-pharmacological treatments in early dementia and MCI use a broad range of outcome measures, with a small proportion being used more than once. Excepting measures of cognition, there is very little commonality between studies. Where previous research has set priorities on outcome measures preferred by PLWD, people with MCI and caregivers, quality of life for example, this has not yet translated into studies measuring new treatments. Further research to understand which outcomes should be prioritised and how they should be measured.

Contributors

EC designed the study, carried out the literature review, the data charting and synthesis, data interpretation, article preparation, article review and correspondence. AMP and VL contributed to the study design, data interpretation, and article review. MC contributed to the data charting.

Funding

EC is supported by a studentship from the ESRC LISS-DTP.

For peer review only - http://bmjopen.bmj.com/site/about/guidelines.xhtml

Competing interests

None declared

For peer review only

For peer review only - http://bmjopen.bmj.com/site/about/guidelines.xhtml

References

1. Organization WH. Global action plan on the public health response to dementia 2017–2025. 2017
2. Prince M, Bryce R, Ferri C. World Alzheimer Report 2011: The benefits of early diagnosis and intervention. 2018
3. Livingston G, Sommerlad A, Orgeta V, et al. Dementia prevention, intervention, and care. *The Lancet* 2017;390(10113):2673-734.
4. Douglas S, James I, Ballard C. Non-pharmacological interventions in dementia. *Advances in psychiatric treatment* 2004;10(3):171-77.
5. Kasl-Godley J, Gatz M. Psychosocial interventions for individuals with dementia: an integration of theory, therapy, and a clinical understanding of dementia. *Clinical psychology review* 2000;20(6):755-82.
6. Olazarán J, Reisberg B, Clare L, et al. Nonpharmacological therapies in Alzheimer's disease: a systematic review of efficacy. *Dementia and geriatric cognitive disorders* 2010;30(2):161-78.
7. Brodaty H, Arasaratnam C. Meta-analysis of nonpharmacological interventions for neuropsychiatric symptoms of dementia. *American Journal of Psychiatry* 2012;169(9):946-53.
8. Bruscoli M, Lovestone S. Is MCI really just early dementia? A systematic review of conversion studies. *International Psychogeriatrics* 2004;16(2):129-40.
9. Portet F, Ousset P, Visser P, et al. Mild cognitive impairment (MCI) in medical practice: a critical review of the concept and new diagnostic procedure. Report of the MCI Working Group of the European Consortium on Alzheimer's Disease. *Journal of Neurology, Neurosurgery & Psychiatry* 2006;77(6):714-18.
10. Allain H, Bentué-Ferrer D, Akwa Y. Treatment of the mild cognitive impairment (MCI). *Human Psychopharmacology: Clinical and Experimental* 2007;22(4):189-97.
11. Petersen RC. MCI treatment trials: failure or not? *The Lancet Neurology* 2007;6(6):473-75.
12. Teixeira CVL, Gobbi LTB, Corazza DI, et al. Non-pharmacological interventions on cognitive functions in older people with mild cognitive impairment (MCI). *Archives of gerontology and geriatrics* 2012;54(1):175-80.
13. Paulino Ramirez Diaz S, Gil Gregório P, Manuel Ribera Casado J, et al. The need for a consensus in the use of assessment tools for Alzheimer's disease: the Feasibility Study (assessment tools for dementia in Alzheimer Centres across Europe), a European Alzheimer's Disease Consortium's (EADC) survey. *International journal of geriatric psychiatry* 2005;20(8):744-48.
14. Moniz-Cook E, Vernooij-Dassen M, Woods R, et al. A European consensus on outcome measures for psychosocial intervention research in dementia care. *Aging and Mental Health* 2008;12(1):14-29.
15. Tricco AC, Lillie E, Zarin W, et al. PRISMA extension for scoping reviews (PRISMA-ScR): checklist and explanation. *Annals of internal medicine* 2018;169(7):467-73.
16. Moher D, Liberati A, Tetzlaff J, et al. Preferred reporting items for systematic reviews and meta-analyses: the PRISMA statement. *Annals of internal medicine* 2009;151(4):264-69.
17. Ouzzani M, Hammady H, Fedorowicz Z, et al. Rayyan—a web and mobile app for systematic reviews. *Systematic reviews* 2016;5(1):210.
18. Greenaway M, Duncan N, Smith G. The memory support system for mild cognitive impairment: randomized trial of a cognitive rehabilitation intervention. *International journal of geriatric psychiatry* 2013;28(4):402-09.
19. Jansen AP, van Hout HP, Nijpels G, et al. Effectiveness of case management among older adults with early symptoms of dementia and their primary informal caregivers: a randomized clinical trial. *International journal of nursing studies* 2011;48(8):933-43.
20. Kinsella GJ, Mullaly E, Rand E, et al. Early intervention for mild cognitive impairment: a randomised controlled trial. *Journal of Neurology, Neurosurgery & Psychiatry* 2009;80(7):730-36.

For peer review only - http://bmjopen.bmj.com/site/about/guidelines.xhtml

21. Koivisto AM, Hallikainen I, Välimäki T, et al. Early psychosocial intervention does not delay institutionalization in persons with mild Alzheimer disease and has impact on neither disease progression nor caregivers' well-being: ALSOVA 3-year follow-up. *International journal of geriatric psychiatry* 2016;31(3):273-83.
22. Lam LC, Lee JS, Chung JC, et al. A randomized controlled trial to examine the effectiveness of case management model for community dwelling older persons with mild dementia in Hong Kong. *International Journal of Geriatric Psychiatry: A journal of the psychiatry of late life and allied sciences* 2010;25(4):395-402.
23. Logsdon RG, Pike KC, McCurry SM, et al. Early-stage memory loss support groups: outcomes from a randomized controlled clinical trial. *Journals of Gerontology Series B: Psychological Sciences and Social Sciences* 2010;65(6):691-97.
24. Tappen RM, Hain D. The effect of in-home cognitive training on functional performance of individuals with mild cognitive impairment and early-stage Alzheimer's disease. *Research in gerontological nursing* 2013;7(1):14-24.
25. Waldorff F, Buss D, Eckermann A, et al. Efficacy of psychosocial intervention in patients with mild Alzheimer's disease: the multicentre, rater blinded, randomised Danish Alzheimer Intervention Study (DAISY). *Bmj* 2012;345:e4693.
26. Hattori H, Hattori C, Hokao C, et al. Controlled study on the cognitive and psychological effect of coloring and drawing in mild Alzheimer's disease patients. *Geriatrics & gerontology international* 2011;11(4):431-37.
27. Rockwood K, Fay S, Gorman M, et al. The clinical meaningfulness of ADAS-Cog changes in Alzheimer's disease patients treated with donepezil in an open-label trial. *BMC neurology* 2007;7(1):26.
28. Sheehan B. Assessment scales in dementia. *Therapeutic advances in neurological disorders* 2012;5(6):349-58.
29. Piccininni M, Di Carlo A, Baldereschi M, et al. Behavioral and psychological symptoms in Alzheimer's disease: frequency and relationship with duration and severity of the disease. *Dementia and geriatric cognitive disorders* 2005;19(5-6):276-81.
30. Brod M, Stewart AL, Sands L, et al. Conceptualization and measurement of quality of life in dementia: the dementia quality of life instrument (DQoL). *The Gerontologist* 1999;39(1):25-36.
31. Barrios PG, González RP, Hanna SM, et al. Priority of treatment outcomes for caregivers and patients with mild cognitive impairment: preliminary analyses. *Neurology and therapy* 2016;5(2):183-92.
32. Schulz R, O'Brien AT, Bookwala J, et al. Psychiatric and physical morbidity effects of dementia caregiving: prevalence, correlates, and causes. *The Gerontologist* 1995;35(6):771-91.
33. Bruce JM, McQuiggan M, Williams V, et al. Burden among spousal and child caregivers of patients with mild cognitive impairment. *Dementia and geriatric cognitive disorders* 2008;25(4):385.
34. Amjad I, Toor H, Niazi IK, et al. Therapeutic effects of aerobic exercise on EEG parameters and higher cognitive functions in mild cognitive impairment patients. *International Journal of Neuroscience* 2019;129(6):551-62.
35. Bae S, Lee S, Lee S, et al. The effect of a multicomponent intervention to promote community activity on cognitive function in older adults with mild cognitive impairment: A randomized controlled trial. *Complementary therapies in medicine* 2019;42:164-69.
36. Baker LD, Frank LL, Foster-Schubert K, et al. Effects of aerobic exercise on mild cognitive impairment: a controlled trial. *Archives of neurology* 2010;67(1):71-79.
37. Belleville S, Hudon C, Bier N, et al. MEMO+: efficacy, durability and effect of cognitive training and psychosocial intervention in individuals with mild cognitive impairment. *Journal of the American Geriatrics Society* 2018;66(4):655-63.

For peer review only - http://bmjopen.bmj.com/site/about/guidelines.xhtml

38. Biasutti M, Mangiacotti A. Assessing a cognitive music training for older participants: a randomised controlled trial. *International journal of geriatric psychiatry* 2018;33(2):271-78.
39. Bono A, Benvenuti C, Buzzi M, et al. Effects of animal assisted therapy (AAT) carried out with dogs on the evolution of mild cognitive impairment. *G Gerontol* 2015;63(1):32-6.
40. Burgio F, Delazer M, Meneghello F, et al. Cognitive training improves ratio processing and decision making in patients with mild cognitive impairment. *Journal of Alzheimer's Disease* 2018(Preprint):1-14.
41. Buschert VC, Teipel SJ, Jolk S, et al. Long-term observation of a multicomponent cognitive intervention in mild cognitive impairment. *The Journal of clinical psychiatry* 2012
42. Carretti B, Borella E, Fostinelli S, et al. Benefits of training working memory in amnestic mild cognitive impairment: specific and transfer effects. *International Psychogeriatrics* 2013;25(4):617-26.
43. Cavallo M, Hunter EM, van der Hiele K, et al. Computerized structured cognitive training in patients affected by early-stage Alzheimer's disease is feasible and effective: a randomized controlled study. *Archives of Clinical Neuropsychology* 2016;31(8):868-76.
44. Chan SC, Lam TL, Fong KN, et al. Generalization of context-specific training in individuals with mild cognitive impairment: an event-related potential study. *Dementia and geriatric cognitive disorders extra* 2016;6(3):568-79.
45. Chan SC, Chan CC, Derbie AY, et al. Chinese calligraphy writing for augmenting attentional control and working memory of older adults at risk of mild cognitive impairment: a randomized controlled trial. *Journal of Alzheimer's Disease* 2017;58(3):735-46.
46. Choi W, Lee S. Ground kayak paddling exercise improves postural balance, muscle performance, and cognitive function in older adults with mild cognitive impairment: a randomized controlled trial. *Medical science monitor: international medical journal of experimental and clinical research* 2018;24:3909.
47. Donnezan LC, Perrot A, Belleville S, et al. Effects of simultaneous aerobic and cognitive training on executive functions, cardiovascular fitness and functional abilities in older adults with mild cognitive impairment. *Mental Health and Physical Activity* 2018;15:78-87.
48. DiNapoli EA, Scogin F, Bryant AN, et al. Effect of individualized social activities on quality of life among older adults with mild to moderate cognitive impairment in a geriatric psychiatry facility. *Aging & mental health* 2016;20(3):262-70.
49. Doi T, Makizako H, Shimada H, et al. Effects of multicomponent exercise on spatial–temporal gait parameters among the elderly with amnestic mild cognitive impairment (aMCI): Preliminary results from a randomized controlled trial (RCT). *Archives of gerontology and geriatrics* 2013;56(1):104-08.
50. Doi T, Verghese J, Makizako H, et al. Effects of cognitive leisure activity on cognition in mild cognitive impairment: results of a randomized controlled trial. *Journal of the American Medical Directors Association* 2017;18(8):686-91.
51. Drumond Marra HL, Myczkowski ML, Maia Memória C, et al. Transcranial magnetic stimulation to address mild cognitive impairment in the elderly: a randomized controlled study. *Behavioural neurology* 2015;2015
52. Emsaki G, NeshatDoost HT, Tavakoli M, et al. Memory specificity training can improve working and prospective memory in amnestic mild cognitive impairment. *Dementia & neuropsychologia* 2017;11(3):255-61.
53. Eyre HA, Siddarth P, Acevedo B, et al. A randomized controlled trial of Kundalini yoga in mild cognitive impairment. *International psychogeriatrics* 2017;29(4):557-67.
54. Feng W, Wang D, Tang L, et al. Effects of Different Cognitive Trainings on Amnestic Mild Cognitive Impairment in the Elderly: A One-Year Longitudinal Functional Magnetic Resonance Imaging (MRI) Study. *Medical science monitor: international medical journal of experimental and clinical research* 2018;24:5517.

For peer review only

55. Fernández-Calvo B, Contador I, Ramos F, et al. Effect of unawareness on rehabilitation outcome in a randomised controlled trial of multicomponent intervention for patients with mild Alzheimer's disease. *Neuropsychological rehabilitation* 2015;25(3):448-77.
56. Singh MAF, Gates N, Saigal N, et al. The Study of Mental and Resistance Training (SMART) study—resistance training and/or cognitive training in mild cognitive impairment: a randomized, double-blind, double-sham controlled trial. *Journal of the American Medical Directors Association* 2014;15(12):873-80.
57. Finn M, McDonald S. Repetition-lag training to improve recollection memory in older people with amnestic mild cognitive impairment. A randomized controlled trial. *Aging, Neuropsychology, and Cognition* 2015;22(2):244-58.
58. Fogarty JN, Murphy KJ, McFarlane B, et al. Taoist Tai Chi® and memory intervention for individuals with mild cognitive impairment. *Journal of aging and physical activity* 2016;24(2):169-80.
59. Förster S, Buschert VC, Teipel SJ, et al. Effects of a 6-month cognitive intervention on brain metabolism in patients with amnestic MCI and mild Alzheimer's disease. *Journal of Alzheimer's Disease* 2011;26(s3):337-48.
60. Galante E, Venturini G, Fiaccadori C. Computer-based cognitive intervention for dementia: preliminary results of a randomized clinical trial. *G Ital Med Lav Ergon* 2007;29(3 Suppl B):B26-32.
61. Hagovská M, Dzvoník O, Olekszyová Z. Comparison of two cognitive training programs with effects on functional activities and quality of life. *Research in gerontological nursing* 2017;10(4):172-80.
62. Hagovska M, Takáč P, Dzvonik O. Effect of a combining cognitive and balanced training on the cognitive, postural and functional status of seniors with a mild cognitive deficit in a randomized, controlled trial. *European journal of physical and rehabilitation medicine* 2016;52(1):101-09.
63. Han JW, Son KL, Byun HJ, et al. Efficacy of the Ubiquitous Spaced Retrieval-based Memory Advancement and Rehabilitation Training (USMART) program among patients with mild cognitive impairment: a randomized controlled crossover trial. *Alzheimer's research & therapy* 2017;9(1):39.
64. Han JW, Lee H, Hong JW, et al. Multimodal cognitive enhancement therapy for patients with mild cognitive impairment and mild dementia: A multi-center, randomized, controlled, double-blind, crossover trial. *Journal of Alzheimer's Disease* 2017;55(2):787-96.
65. Ho RT, Fong TC, Chan WC, et al. Psychophysiological Effects of Dance Movement Therapy and Physical Exercise on Older Adults With Mild Dementia: A Randomized Controlled Trial. *The Journals of Gerontology: Series B*
66. Horie NC, Serrao VT, Simon SS, et al. Cognitive effects of intentional weight loss in elderly obese individuals with mild cognitive impairment. *The Journal of Clinical Endocrinology & Metabolism* 2016;101(3):1104-12.
67. Hyer L, Scott C, Atkinson MM, et al. Cognitive training program to improve working memory in older adults with MCI. *Clinical gerontologist* 2016;39(5):410-27.
68. Jean L, Simard M, Wiederkehr S, et al. Efficacy of a cognitive training programme for mild cognitive impairment: results of a randomised controlled study. *Neuropsychological rehabilitation* 2010;20(3):377-405.
69. Jelcic N, Cagnin A, Meneghello F, et al. Effects of lexical–semantic treatment on memory in early Alzheimer disease: An observer-blinded randomized controlled trial. *Neurorehabilitation and neural repair* 2012;26(8):949-56.
70. Jeong JH, Na HR, Choi SH, et al. Group-and home-based cognitive intervention for patients with mild cognitive impairment: a randomized controlled trial. *Psychotherapy and psychosomatics* 2016;85(4):198-207.

For peer review only - http://bmjopen.bmj.com/site/about/guidelines.xhtml

71. Kohanpour MA, Peeri M, Azarbayjani MA. The effects of aerobic exercise with lavender essence use on cognitive state and serum brain-derived neurotrophic factor levels in elderly with mild cognitive impairment. *Journal of herbmed pharmacology* 2017;6
72. Kovacs E, Sztruhar Jonasne I, Karoczi C, et al. Effects of a multimodal exercise program on balance, functional mobility and fall risk in older adults with cognitive impairment: a randomized controlled single-blind study. *Eur J Phys Rehabil Med* 2013;49(5):639-48.
73. Küster OC, Fissler P, Laptinskaya D, et al. Cognitive change is more positively associated with an active lifestyle than with training interventions in older adults at risk of dementia: a controlled interventional clinical trial. *BMC psychiatry* 2016;16(1):315.
74. Kwok TC, Bai X, Li JC, et al. Effectiveness of cognitive training in Chinese older people with subjective cognitive complaints: A randomized placebo-controlled trial. *International journal of geriatric psychiatry* 2013;28(2):208-15.
75. Lam LC, Chau RC, Wong BM, et al. A 1-year randomized controlled trial comparing mind body exercise (Tai Chi) with stretching and toning exercise on cognitive function in older Chinese adults at risk of cognitive decline. *Journal of the American Medical Directors Association* 2012;13(6):568. e15-68. e20.
76. Lam LC-w, Chan WC, Leung T, et al. Would older adults with mild cognitive impairment adhere to and benefit from a structured lifestyle activity intervention to enhance cognition?: a cluster randomized controlled trial. *PLoS One* 2015;10(3):e0118173.
77. Langoni CdS, Resende TdL, Barcellos AB, et al. The effect of group exercises on balance, mobility, and depressive symptoms in older adults with mild cognitive impairment: a randomized controlled trial. *Clinical rehabilitation* 2019;33(3):439-49.
78. Law LL, Barnett F, Yau MK, et al. Effects of functional tasks exercise on older adults with cognitive impairment at risk of Alzheimer's disease: a randomised controlled trial. *Age and ageing* 2014;43(6):813-20.
79. Lazarou I, Parastatidis T, Tsolaki A, et al. International ballroom dancing against neurodegeneration: a randomized controlled trial in Greek community-dwelling elders with mild cognitive impairment. *American Journal of Alzheimer's Disease & Other Dementias®* 2017;32(8):489-99.
80. Li B-Y, He N-Y, Qiao Y, et al. Computerized cognitive training for Chinese mild cognitive impairment patients: A neuropsychological and fMRI study. *NeuroImage: Clinical* 2019;22:101691.
81. Lim H-W, Saw W-Y, Feng L, et al. Dataset on gene expression in the elderly after Mindfulness Awareness Practice or Health Education Program. *Data in brief* 2018;18:902-12.
82. Luijpen MW, Swaab DF, Sergeant JA, et al. Effects of transcutaneous electrical nerve stimulation (TENS) on memory in elderly with mild cognitive impairment. *Behavioural brain research* 2005;158(2):349-57.
83. Maffei L, Picano E, Andreassi M, et al. Randomized trial on the effects of a combined physical/cognitive training in aged MCI subjects: the Train the Brain study. *Scientific reports* 2017;7:39471.
84. İnel Manav A, Simsek N. The Effect of Reminiscence Therapy With Internet-Based Videos on Cognitive Status and Apathy of Older People With Mild Dementia. *Journal of geriatric psychiatry and neurology* 2019;32(2):104-13.
85. Melendez JC, Torres M, Redondo R, et al. Effectiveness of follow-up reminiscence therapy on autobiographical memory in pathological ageing. *International Journal of Psychology* 2017;52(4):283-90.
86. Nagamatsu LS, Handy TC, Hsu CL, et al. Resistance training promotes cognitive and functional brain plasticity in seniors with probable mild cognitive impairment. *Archives of internal medicine* 2012;172(8):666-68.
87. Olsen C, Pedersen I, Bergland A, et al. Effect of animal-assisted interventions on depression, agitation and quality of life in nursing home residents suffering from cognitive impairment or

For peer review only

dementia: a cluster randomized controlled trial. *International journal of geriatric psychiatry* 2016;31(12):1312-21.
88. Pantoni L, Poggesi A, Diciotti S, et al. Effect of attention training in mild cognitive impairment patients with subcortical vascular changes: the RehAtt Study. *Journal of Alzheimer's Disease* 2017;60(2):615-24.
89. Park J-H, Park J-H. Does cognition-specific computer training have better clinical outcomes than non-specific computer training? A single-blind, randomized controlled trial. *Clinical rehabilitation* 2018;32(2):213-22.
90. Poinsatte K, Smith EE, Torres VO, et al. T and B cell subsets differentially correlate with amyloid deposition and neurocognitive function in patients with amnestic mild cognitive impairment after one year of physical activity. *Exercise immunology review* 2019;25
91. Pongan E, Tillmann B, Leveque Y, et al. Can musical or painting interventions improve chronic pain, mood, quality of life, and cognition in patients with mild Alzheimer's disease? Evidence from a randomized controlled trial. *Journal of Alzheimer's Disease* 2017;60(2):663-77.
92. Poptsi E, Lazarou I, Markou N, et al. A Comparative Single-Blind Randomized Controlled Trial With Language Training in People With Mild Cognitive Impairment. *American Journal of Alzheimer's Disease & Other Dementias®* 2019;34(3):176-87.
93. Qi M, Zhu Y, Zhang L, et al. The effect of aerobic dance intervention on brain spontaneous activity in older adults with mild cognitive impairment: A resting‑state functional MRI study. *Experimental and therapeutic medicine* 2019;17(1):715-22.
94. Rapp S, Brenes G, Marsh A. Memory enhancement training for older adults with mild cognitive impairment: a preliminary study. *Aging & Mental Health* 2002;6(1):5-11.
95. Rojas GJ, Villar V, Iturry M, et al. Efficacy of a cognitive intervention program in patients with mild cognitive impairment. *International Psychogeriatrics* 2013;25(5):825-31.
96. Rozzini L, Costardi D, Chilovi BV, et al. Efficacy of cognitive rehabilitation in patients with mild cognitive impairment treated with cholinesterase inhibitors. *International Journal of Geriatric Psychiatry: A journal of the psychiatry of late life and allied sciences* 2007;22(4):356-60.
97. Savulich G, Piercy T, Fox C, et al. Cognitive training using a novel memory game on an iPad in patients with amnestic mild cognitive impairment (aMCI). *International Journal of Neuropsychopharmacology* 2017;20(8):624-33.
98. Scherder EJ, Van Paasschen J, Deijen J-B, et al. Physical activity and executive functions in the elderly with mild cognitive impairment. *Aging & mental health* 2005;9(3):272-80.
99. Shimada H, Makizako H, Doi T, et al. Effects of combined physical and cognitive exercises on cognition and mobility in patients with mild cognitive impairment: a randomized clinical trial. *Journal of the American Medical Directors Association* 2018;19(7):584-91.
100. Shimizu N, Umemura T, Matsunaga M, et al. Effects of movement music therapy with a percussion instrument on physical and frontal lobe function in older adults with mild cognitive impairment: A randomized controlled trial. *Aging & mental health* 2018;22(12):1614-26.
101. Simon SS, Hampstead BM, P Nucci M, et al. Cognitive and brain activity changes after mnemonic strategy training in amnestic mild cognitive impairment: evidence from a randomized controlled trial. *Frontiers in aging neuroscience* 2018;10:342.
102. Song D, Doris S. Effects of a moderate-intensity aerobic exercise programme on the cognitive function and quality of life of community-dwelling elderly people with mild cognitive impairment: A randomised controlled trial. *International journal of nursing studies* 2019;93:97-105.
103. Suzuki T, Shimada H, Makizako H, et al. Effects of multicomponent exercise on cognitive function in older adults with amnestic mild cognitive impairment: a randomized controlled trial. *BMC neurology* 2012;12(1):128.

For peer review only - http://bmjopen.bmj.com/site/about/guidelines.xhtml

104. Troyer AK, Murphy KJ, Anderson ND, et al. Changing everyday memory behaviour in amnestic mild cognitive impairment: a randomised controlled trial. *Neuropsychological rehabilitation* 2008;18(1):65-88.
105. Tsai C-L, Ukropec J, Ukropcová B, et al. An acute bout of aerobic or strength exercise specifically modifies circulating exerkine levels and neurocognitive functions in elderly individuals with mild cognitive impairment. *NeuroImage: Clinical* 2018;17:272-84.
106. Tsantali E, Economidis D, Rigopoulou S. Testing the benefits of cognitive training vs. cognitive stimulation in mild Alzheimer's disease: a randomised controlled trial. *Brain Impairment* 2017;18(2):188-96.
107. Valdés EG, Andel R, Lister JJ, et al. Can Cognitive Speed of Processing Training Improve Everyday Functioning Among Older Adults With Psychometrically Defined Mild Cognitive Impairment? *Journal of aging and health* 2019;31(4):595-610.
108. Van Uffelen JG, Paw MJCA, Hopman-Rock M, et al. The effect of walking and vitamin B supplementation on quality of life in community-dwelling adults with mild cognitive impairment: a randomized, controlled trial. *Quality of Life Research* 2007;16(7):1137-46.
109. Wei X-h, Ji L-l. Effect of handball training on cognitive ability in elderly with mild cognitive impairment. *Neuroscience letters* 2014;566:98-101.
110. Yang H, Leaver AM, Siddarth P, et al. Neurochemical and neuroanatomical plasticity following memory training and yoga interventions in older adults with mild cognitive impairment. *Frontiers in aging neuroscience* 2016;8:277.
111. Yoon DH, Kang D, Kim Hj, et al. Effect of elastic band-based high-speed power training on cognitive function, physical performance and muscle strength in older women with mild cognitive impairment. *Geriatrics & gerontology international* 2017;17(5):765-72.
112. Young DK, Kwok TC, Ng PY. A single blind randomized control trial on support groups for Chinese persons with mild dementia. *Clinical interventions in aging* 2014;9:2105.
113. Young K-w, Ng P, Kwok T, et al. The effects of holistic health group interventions on improving the cognitive ability of persons with mild cognitive impairment: a randomized controlled trial. *Clinical interventions in aging* 2017;12:1543.
114. Yun K, Song I-U, Chung Y-A. Changes in cerebral glucose metabolism after 3 weeks of noninvasive electrical stimulation of mild cognitive impairment patients. *Alzheimer's research & therapy* 2016;8(1):49.
115. Zhao J, Li H, Lin R, et al. effects of creative expression therapy for older adults with mild cognitive impairment at risk of Alzheimer's disease: a randomized controlled clinical trial. *Clinical interventions in aging* 2018;13:1313.
116. Zhu Y, Wu H, Qi M, et al. Effects of a specially designed aerobic dance routine on mild cognitive impairment. *Clinical interventions in aging* 2018;13:1691.

For peer review only

Table A1. Search Strategy for OVID

	Search term		Search term continued
**1**	Early dementia	**39**	self help group
**2**	Mild dementia	**40**	psychotherapy
**3**	mild alzheimer*	**41**	CBT
**4**	early alzheimer*	**42**	Cognitive behavio?ral therap*
**5**	cognitive impairment	**43**	Cognitive behavioural therap*
**6**	age related cognitive impairment	**44**	Talking therap*
**7**	Mild cognitive impairment	**45**	Individual therap*
**8**	MCI	**46**	Peer support
**9**	mild neurocognitive disorder	**47**	Counselling
**10**	**1 OR 2 OR 3 OR 4 OR 5 OR 6 OR 7 OR 8 OR 9**	**48**	Communication
**11**	cognitive training	**49**	acupuncture therap*
**12**	brain training	**50**	acupuncture
**13**	memory training	**51**	acupuncture points
**14**	Behavio?r therap*	**52**	Transcranial Magnetic Stimulation
**15**	Behavio?r modification	**53**	TMS
**16**	pleasant activit*	**54**	Relaxation therap*
**17**	Cognitive stimulation therapy	**55**	Therap* relaxation
**18**	CST	**56**	Relaxation techniques
**19**	Transcutaneous Electrical Nerve Stimulation	**57**	Early intervention
**20**	TENS	**58**	Alternative therap*
**21**	Exercise	**59**	**11 OR 12 OR 13 OR 14 OR 15 OR 16 OR 17 OR 18 OR 19 OR 20 OR 21 OR 22 OR 23 OR 24 OR 25 OR 26 OR 27 OR 28 OR 29 OR 30 OR 31 OR 32 OR 33 OR 34 OR 35 OR 36 OR 37 OR 38 OR 39 OR 40 OR 41 OR 42 OR 43 OR 44 OR 45 OR 46 OR 47 OR 48 OR 49 OR 50 OR 51 OR 52 OR 53 OR 54 OR 55 OR 56 OR 57 OR 58 OR 59**
**22**	exercise therap*	**60**	randomized controlled trial
**23**	Walking	**61**	randomised controlled trial
**24**	music therap*	**62**	RCT
**15**	reminiscence therap*	**63**	Clinical Trial
**26**	massage therap*	**64**	intervention
**27**	therap* touch	**65**	**60 OR 61 OR 62 OR 63 OR 64 OR 65**
**28**	recreation therap*	**66**	early dementia
**29**	light therap*	**67**	mild dementia
**30**	therap* light	**68**	mild alzheimer*
**31**	sensory stimulation	**69**	early alzheimer*
**32**	multisensory stimulation	**70**	cognitive impairment
**33**	complementary therap*	**71**	age related cognitive impairment
**34**	aromatherapy	**72**	Mild cognitive impairment
**35**	support group	**73**	MCI
**36**	therap* group	**74**	mild neurocognitive disorder
**37**	memory group	**75**	**66 OR 67 OR 68 OR 69 OR 70 OR 71 OR 72 OR 73 OR 74 OR 75**
**38**	self help	**76**	**10 AND 59 AND 75**

For peer review only

For peer review only - http://bmjopen.bmj.com/site/about/guidelines.xhtml

Table 1. Included Studies

Lead Author	Year	Country	Diagnosis	Number of Groups	Group 1	Group 2	Group 3	Group 4	Group 5	Number of measures
Amjad [34]	2019	Pakistan	MCI	2	Aerobic Exercise	Non-Aerobic Exercise	-	-	-	4
Bae [35]	2019	Japan	MCI	2	Multi-Intervention Programme	Active Control	-	-	-	10
Baker [36]	2010	USA	MCI	2	Aerobic Exercise	Stretching	-	-	-	11
Belleville [37]	2018	Canada	MCI	3	Cognitive Training	Psychosocial Intervention	Control	-	-	7
Biasutti [38]	2017	Italy	MCI	2	Cognitive Training	Gym Activities	-	-	-	4
Bono [39]	2015	Italy	MCI	2	Animal Assisted Therapy	Control	-	-	-	4
Burgio [40]	2018	Italy	MCI	2	Numerical Training	Executive Training	-	-	-	13
Buschert [41]	2012	Germany	MCI	2	Cognitive Training	Active Control	-	-	-	5
Carretti [42]	2013	Italy	MCI	2	Cognitive Training	Active Control	-	-	-	16
Cavallo [43]	2016	Italy	Dementia	2	Cognitive Training	Active Control	-	-	-	3
Chan [44]	2016	Hong Kong	MCI	2	Chinese Calligraphy	Computer Activities	-	-	-	13
Chan [45]	2017	Hong Kong	MCI	2	Chinese Calligraphy	Computer Activities	-	-	-	8
Choi [46]	2018	South Korea	MCI	2	Ground Kayaking	Home Exercise Education	-	-	-	7
Combourieu Donnezan [47]	2018	France	MCI	4	Physical Training	Cognitive Training	Simultaneous Cognitive and Physical Training	Control	-	4
DiNapoli [48]	2016	USA	MCI	2	Individualised Social Activities	Control	-	-	-	4
Doi [49]	2013	Japan	MCI	2	Exercise	Active Control	-	-	-	4
Doi [50]	2017	Japan	MCI	3	Dance	Playing Musical Instruments	Health Education Group	-	-	4
Drumond Marra [51]	2015	Brazil	MCI	2	TMS	Sham TMS	-	-	-	6
Emsaki [52]	2017	Iran	MCI	2	Cognitive Training	Active Control	-	-	-	9
Eyre [53]	2017	USA	MCI	2	Yoga	Cognitive Training	-	-	-	10
Feng [54]	2018	China	MCI	2	Single Component Cognitive Training	Multiple Component Cognitive Training	-	-	-	3
Fernandez-Calvo [55]	2015	Spain	Dementia	2	Multi-Intervention Programme	Control	-	-	-	21
Fiatarone Singh [56]	2014	Australia	MCI	4	Progressive resistance training and sham cognitive training	Progressive resistance training and cognitive training	Cognitive training	Control	-	12

For peer review only - http://bmjopen.bmj.com/site/about/guidelines.xhtml

Finn [57]	2015	Australia	MCI	2	Repetition-lag Training	Control	-	-	-	6
Fogarty [58]	2016	Canada	MCI	2	Memory Intervention Program and Tai Chi	Memory Intervention Program	-	-	-	5
Forster [59]	2011	Germany	Both	2	Cognitive Training	Control	-	-	-	10
Galante [60]	2007	Italy	Dementia	2	Cognitive Training	Active Control	-	-	-	12
Greenaway [18]	2013	USA	MCI	2	Memory Support System (Memory Rehabilitation) with Training	Memory Support System without Training	-	-	-	15
Hagovska [61]	2017	Czech Republic	MCI	2	Cognitive Training (Computer Based)	Cognitive Training	-	-	-	0
Hagovska [62]	2016	Czech Republic	MCI	2	Cognitive Training and Dynamic Balance Training	Balance Training	-	-	-	4
Han [63]	2017	South Korea	MCI	2	Ubiquitous Spaced Retrieval-based Memory Advancement and Rehabilitation Training	Control	-	-	-	4
Han [64]	2017	South Korea	Both	2	Multimodal Cognitive Enhancement Therapy	Active Control	-	-	-	7
Hattori [26]	2011	Japan	Dementia	2	Art Therapy	Active Control	-	-	-	4
Ho [65]	2018	Hong Kong	Both	3	Dance Movement Therapy	Physical Exercise	Control	-	-	7
Horie [66]	2016	Brazil	MCI	2	Group Weight Loss Programme	Control	-	-	-	10
Hyer [67]	2016	USA	MCI	2	Cognitive Training (Computer Based)	Active Control	-	-	-	3
Jansen [19]	2011	The Netherlands	Dementia	2	Case Management	Control	-	-	-	5
Jean [68]	2010	Canada	MCI	2	Cognitive Training	Active Control	-	-	-	10
Jelcic [69]	2012	Italy	Dementia	2	Lexical-Semantic Treatment	Cognitive Stimulation	-	-	-	11
Jeong [70]	2016	South Korea	MCI	2	Cognitive Intervention (Group based)	Cognitive Intervention (Home Based)	-	-	-	8
Kinsella [20]	2009	Australia	MCI	2	Cognitive Intervention	Control	-	-	-	4
Kohanpour [71]	2017	Iran	MCI	4	Aerobic Exercise	Lavender Extract	Aerobic Exercise and Lavender Extract	Control	-	14
Koivisto [21]	2016	Finland	Dementia	2	Psychosocial Intervention	Control	-	-	-	7
Kovacs [72]	2013	Hungary	MCI	2	Multimodal Exercise	Control	-	-	-	1
Kuster [73]	2016	Germany	MCI	3	Cognitive Training	Physical Training	Control	-	-	7
Kwok [74]	2012	Hong Kong	MCI	2	Cognitive Training	Active Control	-	-	-	5

For peer review only

Lam [75]	2012	Hong Kong	MCI	2	Tai Chi	Stretching	-	-	-	4
Lam [76]	2015	Hong Kong	MCI	4	Cognitive Training	Cognitive and Physical Training	Physical Training	Social Groups	-	2
Lam [22]	2010	Hong Kong	Dementia	2	Case Management	Control	-	-	-	2
Langoni [77]	2019	Brazil	MCI	2	Group Exercise	Control	-	-	-	14
Law [78]	2014	Hong Kong	MCI	2	Functional Tasks Exercise Programme	Cognitive Training	-	-	-	7
Lazarou [79]	2017	Greece	MCI	2	Ballroom Dancing	Control	-	-	-	5
Li [80]	2019	China	MCI	2	Computerised Cognitive Training	Control	-	-	-	4
Lim [81]	2018	Singapore	MCI	2	Mindfulness	Health Education	-	-	-	5
Logsdon [23]	2010	USA	Dementia	2	Early Stage Memory Loss Support Group	Control	-	-	-	10
Luijpen [82]	2005	The Netherlands	MCI	2	TENs	Sham TENS	-	-	-	6
Maffei [83]	2017	Italy	MCI	2	Multidomain Training	Control	-	-	-	10
Manav [84]	2019	Turkey	Dementia	2	Reminiscence Therapy	Social Interview	-	-	-	6
Melendez [85]	2015	Spain	Both	2	Reminiscence Therapy	Control	-	-	-	6
Nagamatsu [86]	2012	Canada	MCI	2	Aerobic Exercise	Resistance Training	-	-	-	13
Olsen [87]	2016	Norway	Both	2	Animal Assisted Therapy	Control	-	-	-	9
Pantoni [88]	2017	Italy	MCI	2	Attention Process Training	Control	-	-	-	4
Park [89]	2018	South Korea	MCI	2	Cognition specific computer training	Non-specific computer training	-	-	-	5
Poinsatte [90]	2019	USA	MCI	2	Aerobic Exercise	Stretching	-	-	-	3
Pongan [91]	2017	France	Dementia	2	Choral Singing	Painting	-	-	-	14
Poptsi [92]	2018	Greece	MCI	5	Paper Language Tasks	Computer Language Tasks	Oral Language Tasks	Active Control	Control	4
Qi [93]	2019	China	MCI	2	Aerobic Exercise	Control	-	-	-	3
Rapp [94]	2002	USA	MCI	2	Memory Enhancement Training (Multi-Component)	Control	-	-	-	9
Rojas [95]	2013	Argentina	MCI	2	Cognitive Intervention	Control	-	-	-	8
Rozzini [96]	2007	Italy	MCI	2	Cognitive Training and AChEIs	AChEIs	-	-	-	7
Savulich [97]	2017	UK	MCI	2	Cognitive Training	Control	-	-	-	9

For peer review only

For peer review only - http://bmjopen.bmj.com/site/about/guidelines.xhtml

Scherder [98]	2010	The Netherlands	MCI	3	Walking	Hand and Face Exercises	Control	-	-	11
Shimada [99]	2017	Japan	MCI	2	Physical and Cognitive Training	Health Education Group	-	-	-	7
Shimizu [100]	2017	Japan	MCI	2	Movement Music Therapy	Single Training Task	-	-	-	4
Simon [101]	2018	Brazil	MCI	2	Memory Training	Active Control	-	-	-	8
Song [102]	2019	China	MCI	2	Aerobic Exercise	Active Control	-	-	-	4
Suzuki [103]	2012	Japan	MCI	2	Multicomponent Exercise Group	Active Control	-	-	-	6
Tappen [24]	2014	USA	Both	2	Cognitive Training (Home Based)	Life Story Interview	-	-	-	11
Troyer [104]	2008	Canada	MCI	2	Multicomponent Intervention	Control	-	-	-	6
Tsai [105]	2018	Taiwan	MCI	3	Aerobic Exercise	Resistance Training	Control	-	-	7
Tsantali [106]	2017	Greece	Dementia	3	Cognitive Training	Cognitive Stimulation	Control	-	-	5
Valdes [107]	2019	USA	MCI	2	Speed Processing Training	Active Control	-	-	-	2
van Uffelen [108]	2007	The Netherlands	MCI	4	Walking	Placebo Activity	Folic Acid/ Vitamin B Supplements	Placebo Pills	-	3
Waldorff [25]	2012	Denmark	Dementia	2	Multifaceted Counselling, Education and Support	Control	-	-	-	2
Wei [109]	2014	China	MCI	2	Handball Training	Control	-	-	-	8
Yang [110]	2016	USA	MCI	2	Memory Enhancement Training	Yoga	-	-	-	3
Yoon [111]	2017	South Korea	MCI	2	High-Speed Power Strength Training	Low-Speed Strength Training	-	-	-	5
Young [112]	2014	Hong Kong	Dementia	2	Support Groups	Control	-	-	-	4
Young [113]	2017	Hong Kong	MCI	2	Holistic Health Group	Control	-	-	-	4
Yun [114]	2016	South Korea	MCI	2	TDS	Sham TDS	-	-	-	1
Zhao [115]	2018	China	MCI	2	Creative Expression Therapy	Cognitive Training	-	-	-	7
Zhu [116]	2018	China	MCI	2	Dance	Control	-	-	-	7

For peer review only - http://bmjopen.bmj.com/site/about/guidelines.xhtml

Table 2. Outcome measures by domain and subdomains

**Person living with dementia measures**<br>**Domain and subdomain**	**Outcome Measure**	**N**
**Cognition/Memory**		**219**
Cognition	MMSE	37
	Trail Making Test	27
	Digit Span Test	12
	ADAS-Cog	10
	Rey Auditory Test	9
	Rivermead Behavioural Memory Test	9
	Stroop Test	7
	MMQ	7
	Novelli Lexical Test	7
	MoCA	6
	CDR	6
	Verbal Fluency	6
	CERAD-NB	5
	Addenbrooke's Cognitive Examination	4
	Boston Naming Test	4
	Rey Osterrieth Complex Figure Task	4
	Montreal Cognitive Test	3
	Attentional Matrices Test	3
	California Verbal Learning Test	3
	Digit Symbol Coding Test	3
	Hopkins Verbal Learning Test	3
	The Wechsler Memory Scale	3
	CAMcog	2
	Cognitive Failures Test	2
	Color Trails Test	2
	Dementia Rating Scale-2	2
	DSM IV Test	2
	Auditory Verbal Learning Test	2
	Corsi's Block Tapping Test	2
	Frontal Assessment Test	2
	Fuld Object Memory Evaluation	2
	Logical Memory (Subtest of Wechsler Memory Scale)	2
	Prospective and Retrospective Memory Questionnaire	2
	Pyramids & Palm Trees	2
	Questionnaire d'Auto Evaluation de la Memoire	2
	Raven's Coloured Matrices	2
	Repeatable Battery Test	2
	The verbal learning and memory test	2
	Visual Memory Span	2
	Wechsler Adult Intelligence Scale	2
Knowledge of Memory Strategies	Memory Strategy Toolbox	2
	Strategy Knowledge Repertoire	1
Attention	Test of Everyday Attention	2
**Behavioural and Psychological Symptoms of dementia**		**51**
Anxiety/Depression	Geriatric Depression Scale*	21
	Cornell Scale for Depression in Dementia*	7

For peer review only - http://bmjopen.bmj.com/site/about/guidelines.xhtml

	Hospital Anxiety and Depression Scale	4
	Beck Depression Inventory	1
Other	Neuropsychiatric Inventory*	12
	Apathy Evaluation Scale	3
	Revised Memory and behaviour problem checklist*	
**Everyday Living**		**20**
Activities of daily Living	Instrumental Activities of Daily Living*	8
	Bayer Activities of Daily Living Scale	3
	Alzheimer's Disease Cooperative Study Activities of Daily Living Scale	2
	Barthel index	2
Functional Ability	Functional Activities Questionnaire	3
	Functional and Cognitive Assessment Test and Functional Rating Scale for Dementia	2
**Physical Outcomes**		**19**
Physical Performance	Timed Up and Go Test	7
	Gait	3
	Handgrip strength	3
	Stride	2
	Walking Speed	2
Physical Measures	Weight	2
**Quality of Life/Wellbeing**		**15**
Quality of Life	QoL in Alzheimer's Disease*	7
	Dementia Quality of Life Instrument*	3
	EuroQoL EQ 5D*	2
	EQ-VAS	1
Stress	Perceived Stress Scale	1
General Wellbeing	SF-36	1
**Biological Outcome**		**9**
Brain Activity	EEG	4
	MRI	2
Biomarker	BDNF	3
**Adherence to Intervention**		**2**
Adherence to intervention	Adherence	2

Caregiver Measures

**Domain**	**Outcome Measure**	**N**
**Depression**		**5**
	The Center for Epidemiological Studies Depression Scale*	3
	Geriatric Depression Scale	1
	Beck Depression Inventory	1
**Caregiver Burden**		**2**
	Zarit caregiver burden interview*	2
**General Wellbeing**		**1**
	Short Form Health Survey (SF-36)*	1
**Knowledge of Memory Strategies**		**1**
	Strategy Knowledge Repertoire	1
**Quality of Life**		**1**
	EQ-VAS	1

For peer review only - http://bmjopen.bmj.com/site/about/guidelines.xhtml

**Stress**	**1**
Perceived Stress Scale	1

*Measure recommended by INTERDEM Consensus [14]

For peer review only

For peer review only - http://bmjopen.bmj.com/site/about/guidelines.xhtml

Table 3. Outcome measures by diagnosis and intervention

	Number of Studies	BPSD	Biological Outcome	Caregiver Measure	Cognition/ Memory	Everyday Living	Physical Measures	Physical Performance	Quality of Life/ Wellbeing	Task Performance
**Diagnosis**										
Both	6	5	-	1	12	1	-	-	-	-
Dementia	14	16	-	7	42	6	-	-	6	-
MCI	72	30	9	3	165	13	2	17	9	2
**Type of Intervention**										
Animal Assisted Therapy	2	2	-	-	2	1	-	-	-	-
Art-Based Therapy	2	1	-	1	6	1	-	-	-	-
Case Management	2	2	-	3	1	-	-	-	1	-
Chinese Calligraphy	2	1	1	-	4	-	-	-	-	-
Cognitive Training	37	23	2	3	103	11	-	1	6	2
Cognitive Training and Physical Activity	4	-	-	-	14	2	-	2	-	-
Multicomponent Psychosocial Intervention	4	6	-	3	10	2	-	2	3	-
Music Based Intervention	2	1	-	-	7	-	1	2	1	-
Physical Activity	25	11	6	-	53	3	1	10	2	-
Reminiscence Therapy	2	1	-	-	2	-	-	-	-	-
Support Group	3	3	-	1	1	-	-	-	1	-

For peer review only

For peer review only

For peer review only - http://bmjopen.bmj.com/site/about/guidelines.xhtml

Figure 1. Flow Chart of Included Studies

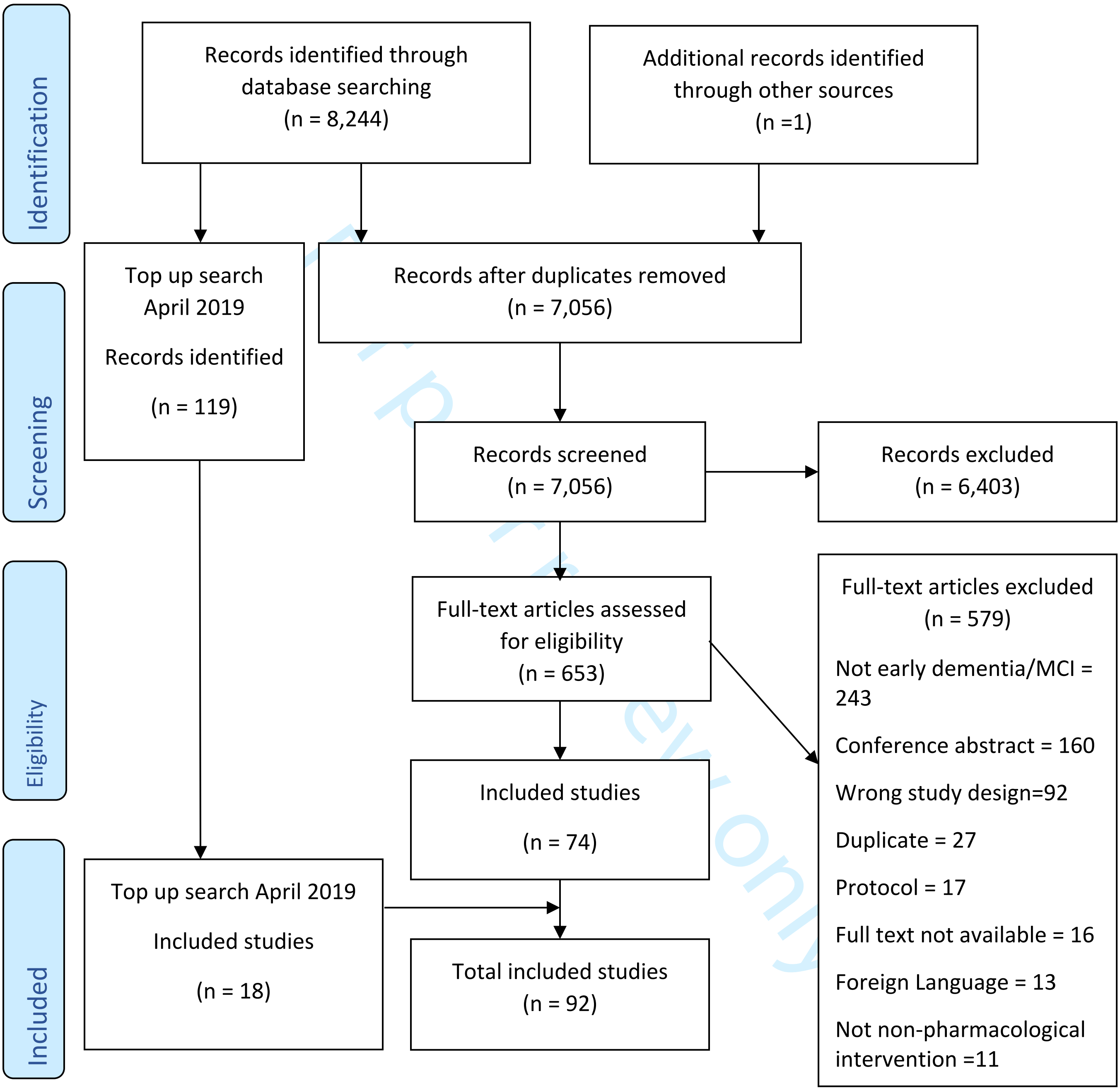

Figure 2. outcome measures over time

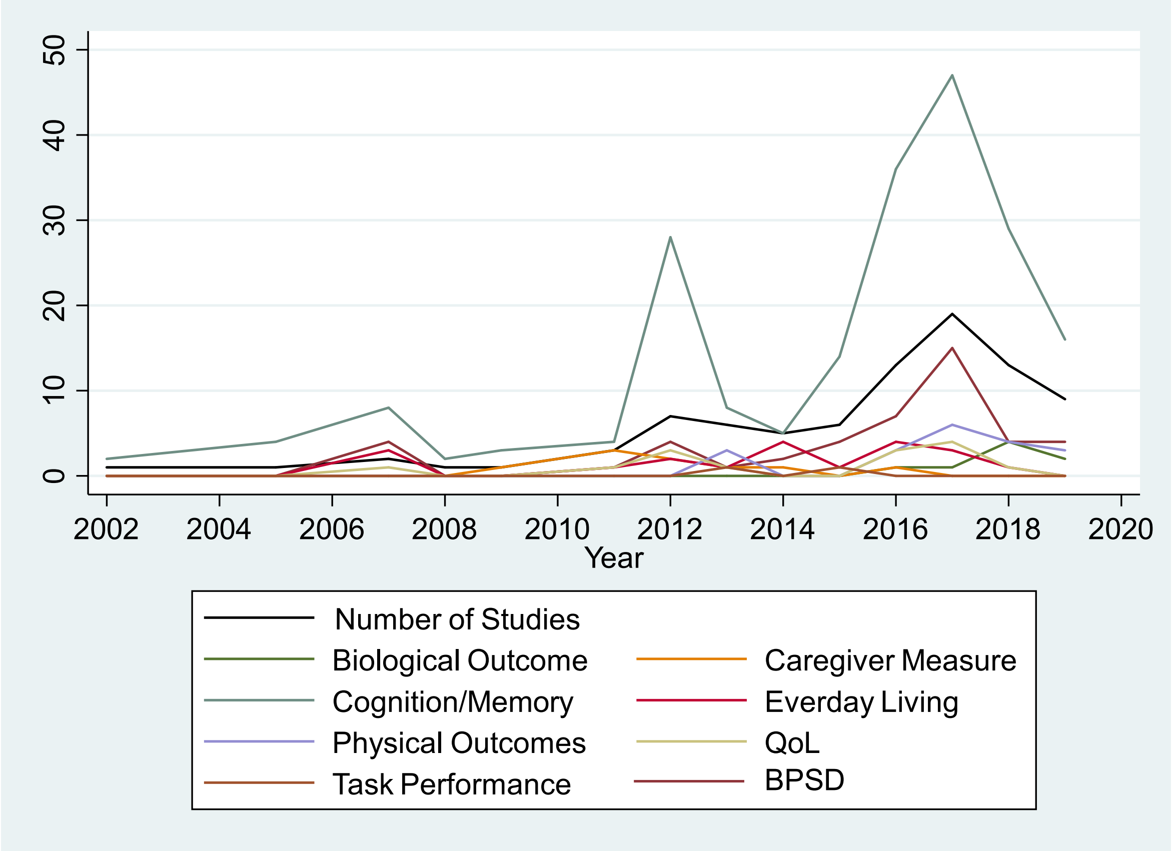

Note: QoL = quality of life; BPSD = behavioural and psychological symptoms of dementia.

Preferred Reporting Items for Systematic reviews and Meta-Analyses extension for Scoping Reviews (PRISMA-ScR) Checklist

SECTION	ITEM	PRISMA-ScR CHECKLIST ITEM	REPORTED ON PAGE #
**TITLE**			
Title	1	Identify the report as a scoping review.	1
**ABSTRACT**			
Structured summary	2	Provide a structured summary that includes (as applicable): background, objectives, eligibility criteria, sources of evidence, charting methods, results, and conclusions that relate to the review questions and objectives.	2
**INTRODUCTION**			
Rationale	3	Describe the rationale for the review in the context of what is already known. Explain why the review questions/objectives lend themselves to a scoping review approach.	5
Objectives	4	Provide an explicit statement of the questions and objectives being addressed with reference to their key elements (e.g., population or participants, concepts, and context) or other relevant key elements used to conceptualize the review questions and/or objectives.	5-6
**METHODS**			
Protocol and registration	5	Indicate whether a review protocol exists; state if and where it can be accessed (e.g., a Web address); and if available, provide registration information, including the registration number.	6
Eligibility criteria	6	Specify characteristics of the sources of evidence used as eligibility criteria (e.g., years considered, language, and publication status), and provide a rationale.	6-7
Information sources*	7	Describe all information sources in the search (e.g., databases with dates of coverage and contact with authors to identify additional sources), as well as the date the most recent search was executed.	7
Search	8	Present the full electronic search strategy for at least 1 database, including any limits used, such that it could be repeated.	Table 1
Selection of sources of evidence†	9	State the process for selecting sources of evidence (i.e., screening and eligibility) included in the scoping review.	8
Data charting process‡	10	Describe the methods of charting data from the included sources of evidence (e.g., calibrated forms or forms that have been tested by the team before their use, and whether data charting was done independently or in duplicate) and any processes for obtaining and confirming data from investigators.	8
Data items	11	List and define all variables for which data were sought and any assumptions and simplifications made.	8
Critical appraisal of individual sources of evidence§	12	If done, provide a rationale for conducting a critical appraisal of included sources of evidence; describe the methods used and how this information was used in any data synthesis (if appropriate).	N/A
Synthesis of results	13	Describe the methods of handling and summarizing the data that were charted.	8

For peer review only

SECTION	ITEM	PRISMA-ScR CHECKLIST ITEM	REPORTED ON PAGE #
**RESULTS**			
Selection of sources of evidence	14	Give numbers of sources of evidence screened, assessed for eligibility, and included in the review, with reasons for exclusions at each stage, ideally using a flow diagram.	9 and Figure 1
Characteristics of sources of evidence	15	For each source of evidence, present characteristics for which data were charted and provide the citations.	9 and Table 2
Critical appraisal within sources of evidence	16	If done, present data on critical appraisal of included sources of evidence (see item 12).	N/A
Results of individual sources of evidence	17	For each included source of evidence, present the relevant data that were charted that relate to the review questions and objectives.	Not feasible
Synthesis of results	18	Summarize and/or present the charting results as they relate to the review questions and objectives.	10-12
**DISCUSSION**			
Summary of evidence	19	Summarize the main results (including an overview of concepts, themes, and types of evidence available), link to the review questions and objectives, and consider the relevance to key groups.	12
Limitations	20	Discuss the limitations of the scoping review process.	14
Conclusions	21	Provide a general interpretation of the results with respect to the review questions and objectives, as well as potential implications and/or next steps.	15
**FUNDING**			
Funding	22	Describe sources of funding for the included sources of evidence, as well as sources of funding for the scoping review. Describe the role of the funders of the scoping review.	15

JBI = Joanna Briggs Institute; PRISMA-ScR = Preferred Reporting Items for Systematic reviews and Meta-Analyses extension for Scoping Reviews.
* Where *sources of evidence* (see second footnote) are compiled from, such as bibliographic databases, social media platforms, and Web sites.
† A more inclusive/heterogeneous term used to account for the different types of evidence or data sources (e.g., quantitative and/or qualitative research, expert opinion, and policy documents) that may be eligible in a scoping review as opposed to only studies. This is not to be confused with *information sources* (see first footnote).
‡ The frameworks by Arksey and O'Malley (6) and Levac and colleagues (7) and the JBI guidance (4, 5) refer to the process of data extraction in a scoping review as data charting.
§ The process of systematically examining research evidence to assess its validity, results, and relevance before using it to inform a decision. This term is used for items 12 and 19 instead of "risk of bias" (which is more applicable to systematic reviews of interventions) to include and acknowledge the various sources of evidence that may be used in a scoping review (e.g., quantitative and/or qualitative research, expert opinion, and policy document).

From: Tricco AC, Lillie E, Zarin W, O'Brien KK, Colquhoun H, Levac D, et al. PRISMA Extension for Scoping Reviews (PRISMA-ScR): Checklist and Explanation. Ann Intern Med. ;169:467–473. doi: 10.7326/M18-0850

For peer review only - http://bmjopen.bmj.com/site/about/guidelines.xhtml

BMJ Open

Outcomes tested in non-pharmacological interventions in mild cognitive impairment and mild dementia: a scoping review

Journal:	*BMJ Open*
Manuscript ID	bmjopen-2019-035980.R1
Article Type:	Original research
Date Submitted by the Author:	30-Jan-2020
Complete List of Authors:	Couch, Elyse; King's College London, Health Service and Population Research<br>Lawrence, Vanessa; King's College London, Health Service and Population Research<br>Co, Melissa; King's College London, Health Service and Population Research<br>Prina, A.Matthew; King's College London, Health Service and Population Research
**Primary Subject Heading**:	Mental health
Secondary Subject Heading:	Geriatric medicine
Keywords:	Dementia < NEUROLOGY, Old age psychiatry < PSYCHIATRY, STATISTICS & RESEARCH METHODS

SCHOLARONE™
Manuscripts

I, the Submitting Author has the right to grant and does grant on behalf of all authors of the Work (as defined in the below author licence), an exclusive licence and/or a non-exclusive licence for contributions from authors who are: i) UK Crown employees; ii) where BMJ has agreed a CC-BY licence shall apply, and/or iii) in accordance with the terms applicable for US Federal Government officers or employees acting as part of their official duties; on a worldwide, perpetual, irrevocable, royalty-free basis to BMJ Publishing Group Ltd ("BMJ") its licensees and where the relevant Journal is co-owned by BMJ to the co-owners of the Journal, to publish the Work in this journal and any other BMJ products and to exploit all rights, as set out in our licence.

The Submitting Author accepts and understands that any supply made under these terms is made by BMJ to the Submitting Author unless you are acting as an employee on behalf of your employer or a postgraduate student of an affiliated institution which is paying any applicable article publishing charge ("APC") for Open Access articles. Where the Submitting Author wishes to make the Work available on an Open Access basis (and intends to pay the relevant APC), the terms of reuse of such Open Access shall be governed by a Creative Commons licence – details of these licences and which Creative Commons licence will apply to this Work are set out in our licence referred to above.

Other than as permitted in any relevant BMJ Author's Self Archiving Policies, I confirm this Work has not been accepted for publication elsewhere, is not being considered for publication elsewhere and does not duplicate material already published. I confirm all authors consent to publication of this Work and authorise the granting of this licence.

For peer review only

Outcomes tested in non-pharmacological interventions in mild cognitive impairment and mild dementia: a scoping review

Elyse Couch[1*], Vanessa Lawrence[1], Melissa Co[1], and A. Matthew Prina[1]

[1] Health Service and Population Research Department, Institute of Psychiatry, Psychology and Neuroscience, King's College London, UK

Elyse.Couch@kcl.ac.uk

Melissa.Co@kcl.ac.uk

Matthew.Prina@kcl.ac.uk

Vanessa.c.Lawrence@kcl.ac.uk

*Corresponding author information: Health Service and Population Research Department, Institute of Psychiatry, Psychology and Neuroscience, King's College London, UK SE5 8AF.

Email: Elyse.Couch@kcl.ac.uk

For peer review only

For peer review only - http://bmjopen.bmj.com/site/about/guidelines.xhtml

Abstract

Objectives: Non-pharmacological treatments are an important aspect of dementia care. A wide range of interventions have been trialled for mild dementia and mild cognitive impairment (MCI). However, the variety of outcome measures used in these trials makes it difficult to make meaningful comparisons. The objective of this study is to map trends which outcome measures are used in trials of non-pharmacological treatments in MCI and mild dementia.

Design: Scoping Review

Data Sources: EMBASE, Psych Info, Medline and the Cochrane Register of Controlled Trials were searched from inception until February 2018. An additional search was conducted in April 2019

Eligibility: We included RCTs testing non-pharmacological interventions for people diagnosed with MCI or early-stage dementia. Studies were restricted to full RCTs; observational, feasibility and pilot studies were not included.

Charting Methods: All outcome measures used by included studies were extracted and grouped thematically. Trends in the types of outcome measures used were explored by type of intervention country and year of publication.

Results: 91 studies were included in this review. We extracted 358 individual outcome measures, of which 78 (22%) were used more than once. Cognitive measures were the most frequently used, with the MMSE being the most popular.

Conclusions: Our findings highlight an inconsistency in the use of outcome measures. Cognition has been prioritised over other domains, despite previous research highlighting the importance of quality of life and caregiver measures. To ensure a robust evidence base, more research is needed to highlight which outcome measures should be used over others.

Protocol Registration**:** The protocol for this study was registered on PROSPERO (ID: CRD42018102649).

For peer review only - http://bmjopen.bmj.com/site/about/guidelines.xhtml

Word count: 4,255

Strengths and Limitations of this study:

- This scoping review has systematically mapped which outcome measures have been used by randomised controlled trials testing non-pharmacological treatments in mild dementia and mild cognitive impairment.
- This review has explored how the use of outcome measures varies by diagnosis, type of intervention, country and year of publication.
- The papers included in this review were limited to full randomised controlled trials, other study designs may be using different types of outcome measures.
- Further research is needed to establish which measures should be used over others.

For peer review only

Introduction

Delivery of both pharmacological and non-pharmacological treatment in the early stages of dementia has been identified as a global priority [1 2]. Current pharmacological treatments for the cognitive symptoms of dementia have been found to have a greater effect when delivered as early as possible [3] however, the benefits of delivering non-pharmacological treatments early are less well understood. Non-pharmacological treatments are an important clinical tool for managing dementia as they are more acceptable to some and less prone to side effects, making them a safe alternative to drug treatments [4]. Those diagnosed earlier in the disease have more cognitive abilities available to engage with non-pharmacological treatments and bolster their own methods for coping with the disease [5]. Previous systematic reviews have found non-pharmacological treatments can improve outcomes; however, these reviews were restricted to a small number of outcome measures [6 7].

Mild cognitive impairment (MCI) has been identified as a potential prodrome for dementia, with approximately 10% of people with MCI converting to a diagnosis of dementia [8]. There is an interest in MCI, as a diagnosis of MCI can facilitate an early diagnosis of dementia and therefore earlier access to dementia services and treatment [9]. MCI is a potentially reversible condition, with many people with MCI reverting back to normal levels of cognition [9] therefore, it is important treatments are available. However, it is not clear which treatments can reverse MCI or prevent conversion to dementia [3]. No drug treatments for MCI have been found to be effective [10 11] and acetylcholinesterase inhibitors are not recommended, however, there is some limited evidence that non-pharmacological interventions may be beneficial [3 12].

Randomised Controlled Trials (RCTs) testing non-pharmacological treatments in dementia and MCI are becoming more common. However, they are highly heterogeneous in terms of participants recruited, quality of the study and the types of interventions they are testing, making it difficult to establish the effectiveness of one treatment over another [6 12 13]. Compounding these issues is the inconsistent use of outcome measures in this area of work [9 14].

For peer review only - http://bmjopen.bmj.com/site/about/guidelines.xhtml

Systematic reviews have identified possible benefits of non-pharmacological treatment, yet meta-analyses are difficult to conduct due to the variation in outcome measures used by studies and typically yield small to moderate effect sizes [6 7]. It is possible that these small effect sizes are due to the selection of outcome measures which either lack sensitivity or the change following the intervention not being in the area covered by the outcome measure. It is important researchers are clear on which domains their interventions are targeting, and which measures are best able to capture this change [15]. Pharmacological treatments target specific biological pathways underlying the disease; therefore, outcome measures have been chosen to reflect this and typically focus on cognitive and functional decline[16]. Non-pharmacological treatments generally do not target the underlying biological pathway of the disease therefore, outcome measures should theoretically differ between pharmacological and non-pharmacological treatments[17]. However, a review on non-pharmacological approaches to treating found that studies tended to pay little attention to the mechanisms of change underlying the intervention[4]. The expected mechanisms of change should affect which outcomes are used in non-pharmacological treatments for mild dementia and MCI.

In addition to being clear on how change arises in non-pharmacological treatments, there needs to be a more coherent use of outcomes and the measures used to capture these between studies to ensure a broad and robust evidence base [15]. In 2008, the INTERDEM group, a consortium of dementia researchers across Europe, did work to draw a consensus on which outcome measures should be used when evaluating non-pharmacological treatments. They recommended 22 measures across nine domains including quality of life, mood, global functioning, behaviour, daily living skills, caregiver mood, caregiver burden and staff morale [15]. This guidance does not explore outcomes by the stage of the disease. The outcome measures were selected based on their applicability to European research. The utility of outcome measures may vary by culture[16], previous reviews exploring the use of outcome measures in dementia research have not investigated how this differs by country[17].

For peer review only - http://bmjopen.bmj.com/site/about/guidelines.xhtml

For peer review only

It is not understood which outcome measures are currently being used in non-pharmacological treatments for early dementia and MCI. Scoping reviews present the opportunity to map the evidence on a topic [18], unlike a systematic review scoping reviews can be used to summarise the evidence in a heterogeneous body of literature. Therefore, the aim of this scoping review is to map trends in which outcome measures are being used in RCTs for non-pharmacological treatments in MCI and mild dementia.

Objectives

The specific objectives of this scoping review are to:

(1) Chart which outcomes measures have been used to assess the effectiveness of nonpharmacological treatments in mild dementia and MCI
(2) Highlight which types of measures have been used most frequently
(3) Explore whether the outcome measures used differ depending on the type of intervention, study population, and country the research was conducted in.

Methods

Protocol registration

The protocol for this review was developed following the guidelines set out by the Preferred Reporting Items for Systematic Reviews and Meta-Analysis Extension (PRISMA) statement [19] and the PRISMA guidelines for Scoping Reviews (PRISMA-ScR) [18]. The protocol was registered prospectively on PROSPERO (ID: CRD42018102649).

Eligibility criteria

We included RCTs testing non-pharmacological interventions for people diagnosed with MCI or mild dementia. Studies were restricted to full RCTs; observational, feasibility and pilot studies were not included.

For peer review only - http://bmjopen.bmj.com/site/about/guidelines.xhtml

For peer review only

Studies were included if they met the following criteria:

- Testing non-pharmacological interventions. Studies were not excluded if participants were also treated with acetylcholinesterase inhibitors.
- Participants had a diagnosis of MCI or mild dementia, which was either diagnosed in clinical practice, or met standardised diagnostic criteria, such as the International Statistical Classification of Diseases (ICD-10) or The Diagnostic Statistical Manual of Mental Disorders (DSM), The National Institute of Communicative disorders and Stroke and the Alzheimer's Disease and Related Disorders (NINCDS-ADRDA), the International working group on MCI criteria, The Consortium to Establish a Registry for Alzheimer's Disease (CERAD), The National Institute on Aging- Alzheimer's Associating Diagnostic Guidelines for Alzheimer's Disease, the Petersen Criteria; or was defined by a standardised clinical measure, such as scores between 24-18 on the Mini-Mental State Exam (MMSE); scores ≤26 on the Montreal Cognitive Assessment (MoCA), scores between 15-27 on the St Louis University Mental Status (SLUMS), a Clinical Dementia Rating (CDR) score of 1 (for dementia) or 0.5 (for MCI); or a 4 (for dementia) or 3 (for MCI) on the Global Deterioration Scale (GDS). Studies which include a mix of participants with early dementia and MCI were included, however, studies which included healthy participants and participants with dementia at the later stages of the disease were excluded.
- The intervention was targeted for the person living with dementia or MCI. Dyadic interventions, interventions delivered to both the person living with dementia and their caregivers, were included. Interventions delivered solely to caregivers or health care professionals were excluded.
- Participants were living in long term care facilities or the community
- Written in English

Studies were excluded if:

For peer review only - http://bmjopen.bmj.com/site/about/guidelines.xhtml

- Only pharmacological interventions were tested
- The participants were diagnosed with vascular cognitive impairment or young-onset dementia or Parkinson's Disease Dementia
- Participants were living in a psychiatric inpatient or acute hospital setting
- The intervention had the primary aim of treating major depressive disorder
- The study tested palliative care interventions or advanced care planning
- The only outcome measures used were economic outcomes, such as cost-effectiveness etc.

Information sources and search strategy

To identify potentially relevant studies, we searched EMBASE, Psych Info, Medline and the Cochrane Register of Controlled Trials from inception until 22nd February 2018. An additional search was conducted on 2nd April 2019. See **Supplementary Table 1** for the final search strategy for MEDLINE, which was adapted for the other databases. The final search results were exported into EndNote where duplicates were removed.

Additional papers were identified by searching the references of included papers and other systematic reviews. Conference abstracts and publications were not included.

Selection of sources of evidence

Study selection was managed in Rayyan, where citations were screened against the inclusion and exclusion criteria. Rayyan is an online app for systematic reviews which allows researchers to create their own coding system for decision making [20]. References were first screened by title and abstract, followed by a full-text screening. A second reviewer (MC) screened 10% of the articles at each stage of the review. Disagreements were resolved by discussions with a third reviewer (MP).

A critical appraisal or assessment of the risk of bias is not necessary for a scoping review [18]. This scoping review is not aiming to critically appraise the cumulative literature of outcome measures for

For peer review only - http://bmjopen.bmj.com/site/about/guidelines.xhtml

non-pharmacological treatment in MCI and mild dementia, therefore we did not conduct a critical appraisal or risk of bias assessment for this review.

Data charting process and data items

Data from eligible studies were charted using a standardised extraction tool designed for this study. Items deemed most relevant to the review objectives were the diagnosis of the study participants, description of interventions being tested, the number of intervention groups, and outcome measures used with references.

Synthesis of results

The charted data were mapped to reflect the objectives of this review. Following data charting, outcome measures which were used more than once across the included studies were grouped by domain. We grouped the interventions thematically by the type of intervention being tested.

We explored which types of outcome measures were used by intervention type, by tabulating the type of intervention against the domain of the outcome measure. We excluded interventions which were only used once from this summary. Results were presented in tables and summarised narratively.

Patient and Participant Involvement

The South London and Maudsley MALADY group, of current and former carers of people living with dementia, were consulted in the planning of this study.

Results

Included studies

After duplicates were removed, a total of 7,056 citations were screened for inclusion, 653 were screened at full text and 76 papers were initially identified. A top-up search in April 2019 identified 119 new citations, 17 were included making the total number of included studies 91, See **Figure 1**.

For peer review only - http://bmjopen.bmj.com/site/about/guidelines.xhtml

The studies included in this review are described in **Table 1**, including diagnosis of included participants, number of intervention groups, details on the interventions and comparisons tested and the number of outcomes measures used. The included studies were published between 2002 and 2019.

The majority of studies included in this review were conducted in the USA (n=10), Hong Kong (n=10), and Italy (n=10). Followed by mainland China (n=7), Japan (n=7), and South Korea (n=7). Studies were also conducted in: Argentina, Australia, Brazil, Canada, Czech Republic, Denmark, France, Finland, Germany, Greece, Hungary, Iran, Norway, Pakistan, Singapore, Spain, Taiwan, The Netherlands, Turkey, and the United Kingdom; these countries had fewer than 5 included studies each.

Most studies only recruited participants with MCI (n=72), followed by mild dementia only (n=15), and six studies recruited both participants with MCI and mild dementia.

Results of individual sources of evidence

We extracted 358 individual outcome measures from the included studies, of these 78 (22%) were used more than once. Out of the 78 measures used more than once, 70 (88%) were measures of participants living with dementia (PLWD), 6 measures were used in both the PLWD and their caregiver, 2 measures were only of the caregiver. The number of outcome measures used by each study ranged between one and 21 with an average of 6.85.

Types of non-pharmacological interventions

We grouped the interventions thematically by type. The most frequently tested type of intervention was cognitive training (n=36) followed by physical activity (n=25), combined physical activity and cognitive training (n=4), multicomponent psychosocial interventions (n=4) and support groups (n=3). Animal-assisted therapies, art-based therapies, case management, Chinese calligraphy, music-based interventions and reminiscence therapy were each tested in two studies.

For peer review only

A group weight loss programme, mindfulness, social activities, transcranial direct current stimulation (TDS), transcutaneous electrical nerve stimulation (TENs), and Transcranial magnetic stimulation (TMS) were each trialled once. These interventions were not included in the analysis of trends in outcome measures.

PLWD outcome measures

Table 2 presents the PLWD specific outcome measures grouped by domain. The most frequently measured domain in PLWD was cognition/memory, which was measured 219 times across the 93 included studies. The most frequent measure of cognition was the MMSE, which was measured 37 times. In addition to measures of memory performance knowledge of memory strategies was measured 3 times in PLWD.

The next most frequently measured domain in PLWD was behavioural and psychological symptoms of dementia (BPSD), within this depression was the most commonly measured BPSD. The Geriatric Depression Scale was the most used measure in this domain, followed by the Neuropsychiatric Inventory which examines a greater number of symptoms. Other BSPDs measured were apathy and agitation resulting from memory problems.

Quality of life and wellbeing were measured 15 times across the included study. Quality of life was measured 15 times using four different instruments, the most popular of which was Logsdon's Quality of Life in Alzheimer's disease scale which was used seven times.

Measures of everyday living, physical ability, biological outcomes and adherence to the intervention delivered in the study were measured less than 20 times across the included studies.

Caregiver measures

Eight interventions in this study were dyadic [21-28], all included outcome measures specific to the caregiver in addition to the PLWD. One study of an intervention solely delivered to the PLWD also included a caregiver specific measure [29].

For peer review only - http://bmjopen.bmj.com/site/about/guidelines.xhtml

Table 2 also presents the outcome measures administered to caregivers grouped by domain. The Center for Epidemiological Studies Depression Scale and the Zarit Caregiver Burden interview were the only measures which were administered solely to caregivers. The other caregiver measures were also administered to PLWD. The most frequently measured domain in caregivers was depression, followed by caregiver burden. General wellbeing, knowledge of memory strategies, quality of life and stress were each measured once.

Use of outcome measures over time

Randomised controlled trials of non-pharmacological treatments in mild dementia and MCI have become more frequent over recent years. Almost half (48%) of studies included in this review were published between 2016 and 2018.

Figure 2 charts trends in outcome measure domains over time. As the number of studies in this area has increased over time, so too has the use of outcome measures in all domains. Cognition/memory has consistently been measured over other domains from the beginning of this sample. The only noticeable trend change is in measures of BPSD, which was generally in line with other domains until around 2012, when it overtakes other domains.

Nearly all studies in 2014 included a measure of everyday living; however, since then, the number of studies including these measures has declined. Where measures of everyday living are being used less, measures of BPSD are being used more.

Similarly, caregiver measures were consistently used until 2011, when in 2010 and 2011 all studies included a caregiver measure, however since then the use of such measures has declined.

Use of outcome measures by intervention

Table 3 presents diagnosis and type of intervention by the domains measured. Cognition/memory was the most measured domain across all diagnostic groups, followed by BPSD. The third most

For peer review only - http://bmjopen.bmj.com/site/about/guidelines.xhtml

For peer review only

common domain for MCI studies was physical performance, whereas caregiver measures were the third most common type of measures used in studies of early dementia,

Cognition/memory was measured in all types of intervention. Measures of BPSD were most common in cognitive training interventions and physical activity interventions, however, they were not used by combined cognitive and physical training interventions. Quality of life was measured by studies of case management, cognitive training, psychosocial interventions, physical activity and support groups.

Caregiver measures were used in five types of interventions. Case management, cognitive training and psychosocial interventions; followed by arts-based therapy and support groups.

Use of outcome measures by country

Table 4 presents the country the research was conducted in by outcome measure domain. Generally, there was not too much variability in the domain of outcome measures used by country. Cognition/memory was the domain most frequently measured by all countries, followed by BPSD. The majority of studies were conducted in China (including Hong Kong and Taiwan), these studies focused on cognition/memory, BPSD and biological outcome measures. Other than China, only three other countries included biological measures (Iran, Pakistan and the USA). The USA had the second largest number of studies included in this review, these studies favoured cognition/memory, BPSD, caregiver measures and quality of life. Out of the 24 countries with studies included in this review, less than half (n=9) included measures of quality of life.

Discussion

In this study, we used a scoping review to map which outcome measures had been used in trials for non-pharmacological treatments of mild dementia and MCI. We extracted 358 individual outcome measures used in 91 trials, only 22% of which were used more than once. We grouped the outcome measures which had been used more than once and examined differences in their use over time, by

For peer review only - http://bmjopen.bmj.com/site/about/guidelines.xhtml

diagnostic group, country the research was set in and by the type of intervention they were being used to evaluate. Measures of cognition and BPSDs were the most frequently used across all studies and types of intervention.

Perhaps unsurprisingly, measures of cognition or memory are the most prevalent across all countries, diagnostic groups and types of intervention with the MMSE being the most frequently used outcome measure, despite the ADAS-cog having been validated as the gold-standard measure of cognition [15 30 31]. Measuring cognition is central to measuring the progression of dementia and is a clinically and empirically useful outcome to measure in dementia research [31]. However, in this review, we charted 40 different measures of cognition. This indicates that while cognition has been prioritised as an outcome in studies of non-pharmacological interventions, there is no consensus between researchers on which specific measures should be used. In addition to measures of cognitive performance, three studies have also measured participants knowledge or retention of memory strategies, indicating an interest in longer-term coping strategies for memory loss.

Measures of the BPSD have become more common over time, becoming in 2017 the most measured outcome after cognition. There is not much variety in the BPSDs which have been measured. Generally, depression was measured over other BPSDs. Other BPSDs such as agitation were measured less, perhaps because they are more associated with the later stages of the disease and depression is associated with the earlier stages [32].

Quality of life and wellbeing were not amongst the most measured domains. Four measures of quality of life were used 13 times across the included studies and all but one of these measures were dementia specific measures. It is surprising quality of life has not been measured more, as previous research has stated that in the absence of a cure, health care providers have a greater ability to improve quality of life than alter the progression of the disease [33]. Furthermore, both people with MCI and caregivers rated quality of life of the patient as the most important outcome to measure, followed by caregiver quality of life/burden [34]. Indicating while quality of life has been identified as a

For peer review only - http://bmjopen.bmj.com/site/about/guidelines.xhtml

priority by PLWD, people diagnosed with MCI and their caregivers in previous research, the findings of this study shows this is not being translated into trials of non-pharmacological treatments for early dementia and MCI.

Likewise, caregiver measures had consistent low use across the studies included in this review. We charted eight caregiver measures which were used 11 times across the included studies. Caregiver measures were more commonly used in studies of PLWD, rather than MCI. Previous research has highlighted the profound effect dementia on their caregivers, with around half of caregivers experiencing high levels of burden [35]. However, a third of caregivers of people with MCI also report extreme levels of burden [36], yet the findings of this study show this is less investigated.

There was great variability in the types of outcomes being used to evaluate the different types of intervention. All studies measured cognition and all but one measured BPSD. A lack of clarity in how change occurs as a result of non-pharmacological treatments is a fundamental weakness in this area of work [4]. It is unlikely that all interventions being tested in this review could hope to improve cognition, however this is the most prevalent domain of outcome measures. There are a number of practical reasons as to why certain outcomes, and therefore outcome measures are used over others, In the past, pharmacological treatments have been required to include some measure of cognition, functional or global assessment[17], it is possible that this approach has influenced the choice in outcomes used in non-pharmacological studies. Furthermore, some measures may be used over others for more practical reasons. For example, measures which are short to administer and free to use may be priorities over others[31]. Several interventions in this review comprise of more than one component, e.g. physical activity and cognitive training. In these cases, it may take multiple measures over many domains to accurately capture change. It is vital that outcome measures are be selected depending on the domains the intervention is seeking to address [31].

In 2008, the INTERDEM group recommended 22 outcome measures for use across nine domains [15]. We found 11 of these 22 measures (50%) were used by the studies included in this review, one of

For peer review only - http://bmjopen.bmj.com/site/about/guidelines.xhtml

the recommended domains (staff carer morale) was not applicable to the studies included in this review. All measures recommended for measuring patient mood, and patient quality of life were charted in this review. Only one of the recommended measures for the activities of daily living, caregiver mood, caregiver burden and caregiver quality of life domains were charted and no measures under the global measures domain were charted in this review. This indicates that there is some consistency between which measures are recommended and which measures are utilised, this is largely for patient measures and there is less consistency for caregiver measures.

In this study, we found that the use of outcome measures did not vary much by the country the study was conducted in. In each country, cognition/memory was the most commonly tested domain, followed by BPSD. The importance of outcomes may vary between cultures; therefore, it is important that the outcomes and measures used reflect this [16]. However, due to the limitations of the methodology used we cannot comment on the cultural relevance of the outcome measures charted in this review. Furthermore, articles were only included if they were published in English. It is possible that more culturally appropriate outcomes were used in articles published in the same language as the population under investigation. This is an important area for future research.

Limitations

The findings of this review must be interpreted in the context of the study. To make this review feasible we only included full RCTs, other outcome measures may have been used in different types of studies. Due to time constraints, some sub-types of dementia and cognitive impairment (young-onset, Parkinson's disease dementia and vascular cognitive impairment) were excluded from this review, which limits the applicability of these findings. Further research is needed to explore whether the pattern in the use of outcomes and outcome measures is similar in these groups, compared with the ones included in this review. Furthermore, only outcome measures which were published could be included in this review. The studies included in this study were heterogeneous in terms of participants recruited, interventions tested, and outcome measures used, making it difficult

For peer review only - http://bmjopen.bmj.com/site/about/guidelines.xhtml

to group them thematically. It is possible some nuance is lost in the exploration of broader themes. As with the nature of scoping reviews, we are only able to present which outcome measures have been used in previous research, we are unable to draw conclusions as to which outcome measures should be used over others. Future research should explore which populations measures have been validated for and what constitutes a clinically useful change.

Implications and recommendations for future research

The findings of this review indicate there is very little consistency in outcome measures used in RCTs for non-pharmacological interventions in MCI and mild dementia, however we are not able to conclude which measures should be used over others. To create a strong evidence base for non-pharmacological treatments more research, with the involvement of PLWD and their carers, is needed to determine which measures are preferable over a greater number of domains. Additionally, the prevalence of cognitive measures found in this study, suggests that researchers are including such measures because there is an expectation to do. Researchers should be clear on the theory behind how their intervention creates change and use the appropriate outcome measures.

Conclusions

In summary, this study has found RCTs for non-pharmacological treatments in mild dementia and MCI use a broad range of outcome measures, with a small proportion being used more than once. Excepting measures of cognition, there is very little commonality between studies. Where previous research has set priorities on outcomes preferred by PLWD, people with MCI and caregivers, quality of life for example, this has not yet translated into studies measuring new treatments. Further research is needed to understand which outcomes should be prioritised and how they should be measured.

Contributors

For peer review only - http://bmjopen.bmj.com/site/about/guidelines.xhtml

EC designed the study, carried out the literature review, the data charting and synthesis, data interpretation, article preparation, article review and correspondence. AMP and VL contributed to the study design, data interpretation, and article review. MC contributed to the data charting.

Funding

EC is supported by a studentship from the ESRC LISS-DTP.

Competing interests

None declared

Data availability statement

No additional data available.

For peer review only

References

1. Organization WH. Global action plan on the public health response to dementia 2017–2025. 2017
2. Prince M, Bryce R, Ferri C. World Alzheimer Report 2011: The benefits of early diagnosis and intervention. 2018
3. Livingston G, Sommerlad A, Orgeta V, et al. Dementia prevention, intervention, and care. *The Lancet* 2017;390(10113):2673-734.
4. Douglas S, James I, Ballard C. Non-pharmacological interventions in dementia. *Advances in psychiatric treatment* 2004;10(3):171-77.
5. Kasl-Godley J, Gatz M. Psychosocial interventions for individuals with dementia: an integration of theory, therapy, and a clinical understanding of dementia. *Clinical psychology review* 2000;20(6):755-82.
6. Olazarán J, Reisberg B, Clare L, et al. Nonpharmacological therapies in Alzheimer's disease: a systematic review of efficacy. *Dementia and geriatric cognitive disorders* 2010;30(2):161-78.
7. Brodaty H, Arasaratnam C. Meta-analysis of nonpharmacological interventions for neuropsychiatric symptoms of dementia. *American Journal of Psychiatry* 2012;169(9):946-53.
8. Bruscoli M, Lovestone S. Is MCI really just early dementia? A systematic review of conversion studies. *International Psychogeriatrics* 2004;16(2):129-40.
9. Portet F, Ousset P, Visser P, et al. Mild cognitive impairment (MCI) in medical practice: a critical review of the concept and new diagnostic procedure. Report of the MCI Working Group of the European Consortium on Alzheimer's Disease. *Journal of Neurology, Neurosurgery & Psychiatry* 2006;77(6):714-18.
10. Allain H, Bentué-Ferrer D, Akwa Y. Treatment of the mild cognitive impairment (MCI). *Human Psychopharmacology: Clinical and Experimental* 2007;22(4):189-97.
11. Petersen RC. MCI treatment trials: failure or not? *The Lancet Neurology* 2007;6(6):473-75.
12. Teixeira CVL, Gobbi LTB, Corazza DI, et al. Non-pharmacological interventions on cognitive functions in older people with mild cognitive impairment (MCI). *Archives of gerontology and geriatrics* 2012;54(1):175-80.
13. Harding AJ, Morbey H, Ahmed F, et al. Developing a core outcome set for people living with dementia at home in their neighbourhoods and communities: study protocol for use in the evaluation of non-pharmacological community-based health and social care interventions. *Trials* 2018;19(1):247.
14. Paulino Ramirez Diaz S, Gil Gregório P, Manuel Ribera Casado J, et al. The need for a consensus in the use of assessment tools for Alzheimer's disease: the Feasibility Study (assessment tools for dementia in Alzheimer Centres across Europe), a European Alzheimer's Disease Consortium's (EADC) survey. *International journal of geriatric psychiatry* 2005;20(8):744-48.
15. Moniz-Cook E, Vernooij-Dassen M, Woods R, et al. A European consensus on outcome measures for psychosocial intervention research in dementia care. *Aging and Mental Health* 2008;12(1):14-29.
16. Katona C, Livingston G, Cooper C, et al. International Psychogeriatric Association consensus statement on defining and measuring treatment benefits in dementia. *International psychogeriatrics* 2007;19(3):345-54.
17. Harrison JK, Noel-Storr AH, Demeyere N, et al. Outcomes measures in a decade of dementia and mild cognitive impairment trials. *Alzheimer's research & therapy* 2016;8(1):48.
18. Tricco AC, Lillie E, Zarin W, et al. PRISMA extension for scoping reviews (PRISMA-ScR): checklist and explanation. *Annals of internal medicine* 2018;169(7):467-73.
19. Moher D, Liberati A, Tetzlaff J, et al. Preferred reporting items for systematic reviews and meta-analyses: the PRISMA statement. *Annals of internal medicine* 2009;151(4):264-69.
20. Ouzzani M, Hammady H, Fedorowicz Z, et al. Rayyan—a web and mobile app for systematic reviews. *Systematic reviews* 2016;5(1):210.

For peer review only - http://bmjopen.bmj.com/site/about/guidelines.xhtml

21. Greenaway M, Duncan N, Smith G. The memory support system for mild cognitive impairment: randomized trial of a cognitive rehabilitation intervention. *International journal of geriatric psychiatry* 2013;28(4):402-09.
22. Jansen AP, van Hout HP, Nijpels G, et al. Effectiveness of case management among older adults with early symptoms of dementia and their primary informal caregivers: a randomized clinical trial. *International journal of nursing studies* 2011;48(8):933-43.
23. Kinsella GJ, Mullaly E, Rand E, et al. Early intervention for mild cognitive impairment: a randomised controlled trial. *Journal of Neurology, Neurosurgery & Psychiatry* 2009;80(7):730-36.
24. Koivisto AM, Hallikainen I, Välimäki T, et al. Early psychosocial intervention does not delay institutionalization in persons with mild Alzheimer disease and has impact on neither disease progression nor caregivers' well-being: ALSOVA 3-year follow-up. *International journal of geriatric psychiatry* 2016;31(3):273-83.
25. Lam LC, Lee JS, Chung JC, et al. A randomized controlled trial to examine the effectiveness of case management model for community dwelling older persons with mild dementia in Hong Kong. *International Journal of Geriatric Psychiatry: A journal of the psychiatry of late life and allied sciences* 2010;25(4):395-402.
26. Logsdon RG, Pike KC, McCurry SM, et al. Early-stage memory loss support groups: outcomes from a randomized controlled clinical trial. *Journals of Gerontology Series B: Psychological Sciences and Social Sciences* 2010;65(6):691-97.
27. Tappen RM, Hain D. The effect of in-home cognitive training on functional performance of individuals with mild cognitive impairment and early-stage Alzheimer's disease. *Research in gerontological nursing* 2013;7(1):14-24.
28. Waldorff F, Buss D, Eckermann A, et al. Efficacy of psychosocial intervention in patients with mild Alzheimer's disease: the multicentre, rater blinded, randomised Danish Alzheimer Intervention Study (DAISY). *Bmj* 2012;345:e4693.
29. Hattori H, Hattori C, Hokao C, et al. Controlled study on the cognitive and psychological effect of coloring and drawing in mild Alzheimer's disease patients. *Geriatrics & gerontology international* 2011;11(4):431-37.
30. Rockwood K, Fay S, Gorman M, et al. The clinical meaningfulness of ADAS-Cog changes in Alzheimer's disease patients treated with donepezil in an open-label trial. *BMC neurology* 2007;7(1):26.
31. Sheehan B. Assessment scales in dementia. *Therapeutic advances in neurological disorders* 2012;5(6):349-58.
32. Piccininni M, Di Carlo A, Baldereschi M, et al. Behavioral and psychological symptoms in Alzheimer's disease: frequency and relationship with duration and severity of the disease. *Dementia and geriatric cognitive disorders* 2005;19(5-6):276-81.
33. Brod M, Stewart AL, Sands L, et al. Conceptualization and measurement of quality of life in dementia: the dementia quality of life instrument (DQoL). *The Gerontologist* 1999;39(1):25-36.
34. Barrios PG, González RP, Hanna SM, et al. Priority of treatment outcomes for caregivers and patients with mild cognitive impairment: preliminary analyses. *Neurology and therapy* 2016;5(2):183-92.
35. Schulz R, O'Brien AT, Bookwala J, et al. Psychiatric and physical morbidity effects of dementia caregiving: prevalence, correlates, and causes. *The Gerontologist* 1995;35(6):771-91.
36. Bruce JM, McQuiggan M, Williams V, et al. Burden among spousal and child caregivers of patients with mild cognitive impairment. *Dementia and geriatric cognitive disorders* 2008;25(4):385.
37. Amjad I, Toor H, Niazi IK, et al. Therapeutic effects of aerobic exercise on EEG parameters and higher cognitive functions in mild cognitive impairment patients. *International Journal of Neuroscience* 2019;129(6):551-62.

For peer review only - http://bmjopen.bmj.com/site/about/guidelines.xhtml

38. Bae S, Lee S, Lee S, et al. The effect of a multicomponent intervention to promote community activity on cognitive function in older adults with mild cognitive impairment: A randomized controlled trial. *Complementary therapies in medicine* 2019;42:164-69.
39. Baker LD, Frank LL, Foster-Schubert K, et al. Effects of aerobic exercise on mild cognitive impairment: a controlled trial. *Archives of neurology* 2010;67(1):71-79.
40. Belleville S, Hudon C, Bier N, et al. MEMO+: efficacy, durability and effect of cognitive training and psychosocial intervention in individuals with mild cognitive impairment. *Journal of the American Geriatrics Society* 2018;66(4):655-63.
41. Biasutti M, Mangiacotti A. Assessing a cognitive music training for older participants: a randomised controlled trial. *International journal of geriatric psychiatry* 2018;33(2):271-78.
42. Bono A, Benvenuti C, Buzzi M, et al. Effects of animal assisted therapy (AAT) carried out with dogs on the evolution of mild cognitive impairment. *G Gerontol* 2015;63(1):32-6.
43. Burgio F, Delazer M, Meneghello F, et al. Cognitive training improves ratio processing and decision making in patients with mild cognitive impairment. *Journal of Alzheimer's Disease* 2018(Preprint):1-14.
44. Buschert VC, Teipel SJ, Jolk S, et al. Long-term observation of a multicomponent cognitive intervention in mild cognitive impairment. *The Journal of clinical psychiatry* 2012
45. Carretti B, Borella E, Fostinelli S, et al. Benefits of training working memory in amnestic mild cognitive impairment: specific and transfer effects. *International Psychogeriatrics* 2013;25(4):617-26.
46. Cavallo M, Hunter EM, van der Hiele K, et al. Computerized structured cognitive training in patients affected by early-stage Alzheimer's disease is feasible and effective: a randomized controlled study. *Archives of Clinical Neuropsychology* 2016;31(8):868-76.
47. Chan SC, Lam TL, Fong KN, et al. Generalization of context-specific training in individuals with mild cognitive impairment: an event-related potential study. *Dementia and geriatric cognitive disorders extra* 2016;6(3):568-79.
48. Chan SC, Chan CC, Derbie AY, et al. Chinese calligraphy writing for augmenting attentional control and working memory of older adults at risk of mild cognitive impairment: a randomized controlled trial. *Journal of Alzheimer's Disease* 2017;58(3):735-46.
49. Choi W, Lee S. Ground kayak paddling exercise improves postural balance, muscle performance, and cognitive function in older adults with mild cognitive impairment: a randomized controlled trial. *Medical science monitor: international medical journal of experimental and clinical research* 2018;24:3909.
50. Donnezan LC, Perrot A, Belleville S, et al. Effects of simultaneous aerobic and cognitive training on executive functions, cardiovascular fitness and functional abilities in older adults with mild cognitive impairment. *Mental Health and Physical Activity* 2018;15:78-87.
51. DiNapoli EA, Scogin F, Bryant AN, et al. Effect of individualized social activities on quality of life among older adults with mild to moderate cognitive impairment in a geriatric psychiatry facility. *Aging & mental health* 2016;20(3):262-70.
52. Doi T, Makizako H, Shimada H, et al. Effects of multicomponent exercise on spatial–temporal gait parameters among the elderly with amnestic mild cognitive impairment (aMCI): Preliminary results from a randomized controlled trial (RCT). *Archives of gerontology and geriatrics* 2013;56(1):104-08.
53. Doi T, Verghese J, Makizako H, et al. Effects of cognitive leisure activity on cognition in mild cognitive impairment: results of a randomized controlled trial. *Journal of the American Medical Directors Association* 2017;18(8):686-91.
54. Drumond Marra HL, Myczkowski ML, Maia Memória C, et al. Transcranial magnetic stimulation to address mild cognitive impairment in the elderly: a randomized controlled study. *Behavioural neurology* 2015;2015

For peer review only - http://bmjopen.bmj.com/site/about/guidelines.xhtml

55. Emsaki G, NeshatDoost HT, Tavakoli M, et al. Memory specificity training can improve working and prospective memory in amnestic mild cognitive impairment. *Dementia & neuropsychologia* 2017;11(3):255-61.
56. Eyre HA, Siddarth P, Acevedo B, et al. A randomized controlled trial of Kundalini yoga in mild cognitive impairment. *International psychogeriatrics* 2017;29(4):557-67.
57. Feng W, Wang D, Tang L, et al. Effects of Different Cognitive Trainings on Amnestic Mild Cognitive Impairment in the Elderly: A One-Year Longitudinal Functional Magnetic Resonance Imaging (MRI) Study. *Medical science monitor: international medical journal of experimental and clinical research* 2018;24:5517.
58. Fernández-Calvo B, Contador I, Ramos F, et al. Effect of unawareness on rehabilitation outcome in a randomised controlled trial of multicomponent intervention for patients with mild Alzheimer's disease. *Neuropsychological rehabilitation* 2015;25(3):448-77.
59. Singh MAF, Gates N, Saigal N, et al. The Study of Mental and Resistance Training (SMART) study—resistance training and/or cognitive training in mild cognitive impairment: a randomized, double-blind, double-sham controlled trial. *Journal of the American Medical Directors Association* 2014;15(12):873-80.
60. Finn M, McDonald S. Repetition-lag training to improve recollection memory in older people with amnestic mild cognitive impairment. A randomized controlled trial. *Aging, Neuropsychology, and Cognition* 2015;22(2):244-58.
61. Fogarty JN, Murphy KJ, McFarlane B, et al. Taoist Tai Chi® and memory intervention for individuals with mild cognitive impairment. *Journal of aging and physical activity* 2016;24(2):169-80.
62. Förster S, Buschert VC, Teipel SJ, et al. Effects of a 6-month cognitive intervention on brain metabolism in patients with amnestic MCI and mild Alzheimer's disease. *Journal of Alzheimer's Disease* 2011;26(s3):337-48.
63. Galante E, Venturini G, Fiaccadori C. Computer-based cognitive intervention for dementia: preliminary results of a randomized clinical trial. *G Ital Med Lav Ergon* 2007;29(3 Suppl B):B26-32.
64. Hagovská M, Dzvoník O, Olekszyová Z. Comparison of two cognitive training programs with effects on functional activities and quality of life. *Research in gerontological nursing* 2017;10(4):172-80.
65. Hagovska M, Takáč P, Dzvonik O. Effect of a combining cognitive and balanced training on the cognitive, postural and functional status of seniors with a mild cognitive deficit in a randomized, controlled trial. *European journal of physical and rehabilitation medicine* 2016;52(1):101-09.
66. Han JW, Son KL, Byun HJ, et al. Efficacy of the Ubiquitous Spaced Retrieval-based Memory Advancement and Rehabilitation Training (USMART) program among patients with mild cognitive impairment: a randomized controlled crossover trial. *Alzheimer's research & therapy* 2017;9(1):39.
67. Han JW, Lee H, Hong JW, et al. Multimodal cognitive enhancement therapy for patients with mild cognitive impairment and mild dementia: A multi-center, randomized, controlled, double-blind, crossover trial. *Journal of Alzheimer's Disease* 2017;55(2):787-96.
68. Ho RT, Fong TC, Chan WC, et al. Psychophysiological Effects of Dance Movement Therapy and Physical Exercise on Older Adults With Mild Dementia: A Randomized Controlled Trial. *The Journals of Gerontology: Series B*
69. Horie NC, Serrao VT, Simon SS, et al. Cognitive effects of intentional weight loss in elderly obese individuals with mild cognitive impairment. *The Journal of Clinical Endocrinology & Metabolism* 2016;101(3):1104-12.
70. Hyer L, Scott C, Atkinson MM, et al. Cognitive training program to improve working memory in older adults with MCI. *Clinical gerontologist* 2016;39(5):410-27.

For peer review only - http://bmjopen.bmj.com/site/about/guidelines.xhtml

71. Jean L, Simard M, Wiederkehr S, et al. Efficacy of a cognitive training programme for mild cognitive impairment: results of a randomised controlled study. *Neuropsychological rehabilitation* 2010;20(3):377-405.
72. Jelcic N, Cagnin A, Meneghello F, et al. Effects of lexical–semantic treatment on memory in early Alzheimer disease: An observer-blinded randomized controlled trial. *Neurorehabilitation and neural repair* 2012;26(8):949-56.
73. Jeong JH, Na HR, Choi SH, et al. Group-and home-based cognitive intervention for patients with mild cognitive impairment: a randomized controlled trial. *Psychotherapy and psychosomatics* 2016;85(4):198-207.
74. Kohanpour MA, Peeri M, Azarbayjani MA. The effects of aerobic exercise with lavender essence use on cognitive state and serum brain-derived neurotrophic factor levels in elderly with mild cognitive impairment. *Journal of herbmed pharmacology* 2017;6
75. Kovacs E, Sztruhar Jonasne I, Karoczi C, et al. Effects of a multimodal exercise program on balance, functional mobility and fall risk in older adults with cognitive impairment: a randomized controlled single-blind study. *Eur J Phys Rehabil Med* 2013;49(5):639-48.
76. Küster OC, Fissler P, Laptinskaya D, et al. Cognitive change is more positively associated with an active lifestyle than with training interventions in older adults at risk of dementia: a controlled interventional clinical trial. *BMC psychiatry* 2016;16(1):315.
77. Kwok TC, Bai X, Li JC, et al. Effectiveness of cognitive training in Chinese older people with subjective cognitive complaints: A randomized placebo-controlled trial. *International journal of geriatric psychiatry* 2013;28(2):208-15.
78. Lam LC, Chau RC, Wong BM, et al. A 1-year randomized controlled trial comparing mind body exercise (Tai Chi) with stretching and toning exercise on cognitive function in older Chinese adults at risk of cognitive decline. *Journal of the American Medical Directors Association* 2012;13(6):568. e15-68. e20.
79. Lam LC-w, Chan WC, Leung T, et al. Would older adults with mild cognitive impairment adhere to and benefit from a structured lifestyle activity intervention to enhance cognition?: a cluster randomized controlled trial. *PLoS One* 2015;10(3):e0118173.
80. Langoni CdS, Resende TdL, Barcellos AB, et al. The effect of group exercises on balance, mobility, and depressive symptoms in older adults with mild cognitive impairment: a randomized controlled trial. *Clinical rehabilitation* 2019;33(3):439-49.
81. Law LL, Barnett F, Yau MK, et al. Effects of functional tasks exercise on older adults with cognitive impairment at risk of Alzheimer's disease: a randomised controlled trial. *Age and ageing* 2014;43(6):813-20.
82. Lazarou I, Parastatidis T, Tsolaki A, et al. International ballroom dancing against neurodegeneration: a randomized controlled trial in Greek community-dwelling elders with mild cognitive impairment. *American Journal of Alzheimer's Disease & Other Dementias®* 2017;32(8):489-99.
83. Li B-Y, He N-Y, Qiao Y, et al. Computerized cognitive training for Chinese mild cognitive impairment patients: A neuropsychological and fMRI study. *NeuroImage: Clinical* 2019;22:101691.
84. Lim H-W, Saw W-Y, Feng L, et al. Dataset on gene expression in the elderly after Mindfulness Awareness Practice or Health Education Program. *Data in brief* 2018;18:902-12.
85. Luijpen MW, Swaab DF, Sergeant JA, et al. Effects of transcutaneous electrical nerve stimulation (TENS) on memory in elderly with mild cognitive impairment. *Behavioural brain research* 2005;158(2):349-57.
86. Maffei L, Picano E, Andreassi M, et al. Randomized trial on the effects of a combined physical/cognitive training in aged MCI subjects: the Train the Brain study. *Scientific reports* 2017;7:39471.

For peer review only - http://bmjopen.bmj.com/site/about/guidelines.xhtml

87. İnel Manav A, Simsek N. The Effect of Reminiscence Therapy With Internet-Based Videos on Cognitive Status and Apathy of Older People With Mild Dementia. *Journal of geriatric psychiatry and neurology* 2019;32(2):104-13.
88. Melendez JC, Torres M, Redondo R, et al. Effectiveness of follow-up reminiscence therapy on autobiographical memory in pathological ageing. *International Journal of Psychology* 2017;52(4):283-90.
89. Nagamatsu LS, Handy TC, Hsu CL, et al. Resistance training promotes cognitive and functional brain plasticity in seniors with probable mild cognitive impairment. *Archives of internal medicine* 2012;172(8):666-68.
90. Olsen C, Pedersen I, Bergland A, et al. Effect of animal-assisted interventions on depression, agitation and quality of life in nursing home residents suffering from cognitive impairment or dementia: a cluster randomized controlled trial. *International journal of geriatric psychiatry* 2016;31(12):1312-21.
91. Pantoni L, Poggesi A, Diciotti S, et al. Effect of attention training in mild cognitive impairment patients with subcortical vascular changes: the RehAtt Study. *Journal of Alzheimer's Disease* 2017;60(2):615-24.
92. Park J-H, Park J-H. Does cognition-specific computer training have better clinical outcomes than non-specific computer training? A single-blind, randomized controlled trial. *Clinical rehabilitation* 2018;32(2):213-22.
93. Poinsatte K, Smith EE, Torres VO, et al. T and B cell subsets differentially correlate with amyloid deposition and neurocognitive function in patients with amnestic mild cognitive impairment after one year of physical activity. *Exercise immunology review* 2019;25
94. Pongan E, Tillmann B, Leveque Y, et al. Can musical or painting interventions improve chronic pain, mood, quality of life, and cognition in patients with mild Alzheimer's disease? Evidence from a randomized controlled trial. *Journal of Alzheimer's Disease* 2017;60(2):663-77.
95. Poptsi E, Lazarou I, Markou N, et al. A Comparative Single-Blind Randomized Controlled Trial With Language Training in People With Mild Cognitive Impairment. *American Journal of Alzheimer's Disease & Other Dementias®* 2019;34(3):176-87.
96. Qi M, Zhu Y, Zhang L, et al. The effect of aerobic dance intervention on brain spontaneous activity in older adults with mild cognitive impairment: A resting-state functional MRI study. *Experimental and therapeutic medicine* 2019;17(1):715-22.
97. Rapp S, Brenes G, Marsh A. Memory enhancement training for older adults with mild cognitive impairment: a preliminary study. *Aging & Mental Health* 2002;6(1):5-11.
98. Rojas GJ, Villar V, Iturry M, et al. Efficacy of a cognitive intervention program in patients with mild cognitive impairment. *International Psychogeriatrics* 2013;25(5):825-31.
99. Rozzini L, Costardi D, Chilovi BV, et al. Efficacy of cognitive rehabilitation in patients with mild cognitive impairment treated with cholinesterase inhibitors. *International Journal of Geriatric Psychiatry: A journal of the psychiatry of late life and allied sciences* 2007;22(4):356-60.
100. Savulich G, Piercy T, Fox C, et al. Cognitive training using a novel memory game on an iPad in patients with amnestic mild cognitive impairment (aMCI). *International Journal of Neuropsychopharmacology* 2017;20(8):624-33.
101. Scherder EJ, Van Paasschen J, Deijen J-B, et al. Physical activity and executive functions in the elderly with mild cognitive impairment. *Aging & mental health* 2005;9(3):272-80.
102. Shimada H, Makizako H, Doi T, et al. Effects of combined physical and cognitive exercises on cognition and mobility in patients with mild cognitive impairment: a randomized clinical trial. *Journal of the American Medical Directors Association* 2018;19(7):584-91.
103. Shimizu N, Umemura T, Matsunaga M, et al. Effects of movement music therapy with a percussion instrument on physical and frontal lobe function in older adults with mild cognitive impairment: A randomized controlled trial. *Aging & mental health* 2018;22(12):1614-26.

For peer review only - http://bmjopen.bmj.com/site/about/guidelines.xhtml

104. Simon SS, Hampstead BM, P Nucci M, et al. Cognitive and brain activity changes after mnemonic strategy training in amnestic mild cognitive impairment: evidence from a randomized controlled trial. *Frontiers in aging neuroscience* 2018;10:342.
105. Song D, Doris S. Effects of a moderate-intensity aerobic exercise programme on the cognitive function and quality of life of community-dwelling elderly people with mild cognitive impairment: A randomised controlled trial. *International journal of nursing studies* 2019;93:97-105.
106. Suzuki T, Shimada H, Makizako H, et al. Effects of multicomponent exercise on cognitive function in older adults with amnestic mild cognitive impairment: a randomized controlled trial. *BMC neurology* 2012;12(1):128.
107. Troyer AK, Murphy KJ, Anderson ND, et al. Changing everyday memory behaviour in amnestic mild cognitive impairment: a randomised controlled trial. *Neuropsychological rehabilitation* 2008;18(1):65-88.
108. Tsai C-L, Ukropec J, Ukropcová B, et al. An acute bout of aerobic or strength exercise specifically modifies circulating exerkine levels and neurocognitive functions in elderly individuals with mild cognitive impairment. *NeuroImage: Clinical* 2018;17:272-84.
109. Tsantali E, Economidis D, Rigopoulou S. Testing the benefits of cognitive training vs. cognitive stimulation in mild Alzheimer's disease: a randomised controlled trial. *Brain Impairment* 2017;18(2):188-96.
110. Van Uffelen JG, Paw MJCA, Hopman-Rock M, et al. The effect of walking and vitamin B supplementation on quality of life in community-dwelling adults with mild cognitive impairment: a randomized, controlled trial. *Quality of Life Research* 2007;16(7):1137-46.
111. Wei X-h, Ji L-l. Effect of handball training on cognitive ability in elderly with mild cognitive impairment. *Neuroscience letters* 2014;566:98-101.
112. Yang H, Leaver AM, Siddarth P, et al. Neurochemical and neuroanatomical plasticity following memory training and yoga interventions in older adults with mild cognitive impairment. *Frontiers in aging neuroscience* 2016;8:277.
113. Yoon DH, Kang D, Kim Hj, et al. Effect of elastic band-based high-speed power training on cognitive function, physical performance and muscle strength in older women with mild cognitive impairment. *Geriatrics & gerontology international* 2017;17(5):765-72.
114. Young DK, Kwok TC, Ng PY. A single blind randomized control trial on support groups for Chinese persons with mild dementia. *Clinical interventions in aging* 2014;9:2105.
115. Young K-w, Ng P, Kwok T, et al. The effects of holistic health group interventions on improving the cognitive ability of persons with mild cognitive impairment: a randomized controlled trial. *Clinical interventions in aging* 2017;12:1543.
116. Yun K, Song I-U, Chung Y-A. Changes in cerebral glucose metabolism after 3 weeks of noninvasive electrical stimulation of mild cognitive impairment patients. *Alzheimer's research & therapy* 2016;8(1):49.
117. Zhao J, Li H, Lin R, et al. effects of creative expression therapy for older adults with mild cognitive impairment at risk of Alzheimer's disease: a randomized controlled clinical trial. *Clinical interventions in aging* 2018;13:1313.
118. Zhu Y, Wu H, Qi M, et al. Effects of a specially designed aerobic dance routine on mild cognitive impairment. *Clinical interventions in aging* 2018;13:1691.

For peer review only - http://bmjopen.bmj.com/site/about/guidelines.xhtml

Table 1. Included Studies

Lead Author	Year	Country	Diagnosis	Number of Groups	Group 1	Group 2	Group 3	Group 4	Group 5	Number of measures
Amjad [37]	2019	Pakistan	MCI	2	Aerobic Exercise	Non-Aerobic Exercise	-	-	-	4
Bae [38]	2019	Japan	MCI	2	Multi-Intervention Programme	Active Control	-	-	-	10
Baker [39]	2010	USA	MCI	2	Aerobic Exercise	Stretching	-	-	-	11
Belleville [40]	2018	Canada	MCI	3	Cognitive Training	Psychosocial Intervention	Control	-	-	7
Biasutti [41]	2017	Italy	MCI	2	Cognitive Training	Gym Activities	-	-	-	4
Bono [42]	2015	Italy	MCI	2	Animal Assisted Therapy	Control	-	-	-	4
Burgio [43]	2018	Italy	MCI	2	Numerical Training	Executive Training	-	-	-	13
Buschert [44]	2012	Germany	MCI	2	Cognitive Training	Active Control	-	-	-	5
Carretti [45]	2013	Italy	MCI	2	Cognitive Training	Active Control	-	-	-	16
Cavallo [46]	2016	Italy	Dementia	2	Cognitive Training	Active Control	-	-	-	3
Chan [47]	2016	Hong Kong	MCI	2	Chinese Calligraphy	Computer Activities	-	-	-	13
Chan [48]	2017	Hong Kong	MCI	2	Chinese Calligraphy	Computer Activities	-	-	-	8
Choi [49]	2018	South Korea	MCI	2	Ground Kayaking	Home Exercise Education	-	-	-	7
Combourieu Donnezan [50]	2018	France	MCI	4	Physical Training	Cognitive Training	Simultaneous Cognitive and Physical Training	Control	-	4
DiNapoli [51]	2016	USA	MCI	2	Individualised Social Activities	Control	-	-	-	4
Doi [52]	2013	Japan	MCI	2	Exercise	Active Control	-	-	-	4
Doi [53]	2017	Japan	MCI	3	Dance	Playing Musical Instruments	Health Education Group	-	-	4
Drumond Marra [54]	2015	Brazil	MCI	2	TMS	Sham TMS	-	-	-	6
Emsaki [55]	2017	Iran	MCI	2	Cognitive Training	Active Control	-	-	-	9
Eyre [56]	2017	USA	MCI	2	Yoga	Cognitive Training	-	-	-	10
Feng [57]	2018	China	MCI	2	Single Component Cognitive Training	Multiple Component Cognitive Training	-	-	-	3
Fernandez-Calvo [58]	2015	Spain	Dementia	2	Multi-Intervention Programme	Control	-	-	-	21
Fiatarone Singh [59]	2014	Australia	MCI	4	Progressive resistance training and sham cognitive training	Progressive resistance training and cognitive training	Cognitive training	Control	-	12

For peer review only - http://bmjopen.bmj.com/site/about/guidelines.xhtml

Finn [60]	2015	Australia	MCI	2	Repetition-lag Training	Control	-	-	-	6
Fogarty [61]	2016	Canada	MCI	2	Memory Intervention Program and Tai Chi	Memory Intervention Program	-	-	-	5
Forster [62]	2011	Germany	Both	2	Cognitive Training	Control	-	-	-	10
Galante [63]	2007	Italy	Dementia	2	Cognitive Training	Active Control	-	-	-	12
Greenaway [21]	2013	USA	MCI	2	Memory Support System (Memory Rehabilitation) with Training	Memory Support System without Training	-	-	-	15
Hagovska [64]	2017	Czech Republic	MCI	2	Cognitive Training (Computer Based)	Cognitive Training	-	-	-	0
Hagovska [65]	2016	Czech Republic	MCI	2	Cognitive Training and Dynamic Balance Training	Balance Training	-	-	-	4
Han [66]	2017	South Korea	MCI	2	Ubiquitous Spaced Retrieval-based Memory Advancement and Rehabilitation Training	Control	-	-	-	4
Han [67]	2017	South Korea	Both	2	Multimodal Cognitive Enhancement Therapy	Active Control	-	-	-	7
Hattori [29]	2011	Japan	Dementia	2	Art Therapy	Active Control	-	-	-	4
Ho [68]	2018	Hong Kong	Both	3	Dance Movement Therapy	Physical Exercise	Control	-	-	7
Horie [69]	2016	Brazil	MCI	2	Group Weight Loss Programme	Control	-	-	-	10
Hyer [70]	2016	USA	MCI	2	Cognitive Training (Computer Based)	Active Control	-	-	-	3
Jansen [22]	2011	The Netherlands	Dementia	2	Case Management	Control	-	-	-	5
Jean [71]	2010	Canada	MCI	2	Cognitive Training	Active Control	-	-	-	10
Jelcic [72]	2012	Italy	Dementia	2	Lexical-Semantic Treatment	Cognitive Stimulation	-	-	-	11
Jeong [73]	2016	South Korea	MCI	2	Cognitive Intervention (Group based)	Cognitive Intervention (Home Based)	-	-	-	8
Kinsella [23]	2009	Australia	MCI	2	Cognitive Intervention	Control	-	-	-	4
Kohanpour [74]	2017	Iran	MCI	4	Aerobic Exercise	Lavender Extract	Aerobic Exercise and Lavender Extract	Control	-	14
Koivisto [24]	2016	Finland	Dementia	2	Psychosocial Intervention	Control	-	-	-	7
Kovacs [75]	2013	Hungary	MCI	2	Multimodal Exercise	Control	-	-	-	1
Kuster [76]	2016	Germany	MCI	3	Cognitive Training	Physical Training	Control	-	-	7
Kwok [77]	2012	Hong Kong	MCI	2	Cognitive Training	Active Control	-	-	-	5

For peer review only - http://bmjopen.bmj.com/site/about/guidelines.xhtml

For peer review only

Lam [78]	2012	Hong Kong	MCI	2	Tai Chi	Stretching	-	-	-	4
Lam [79]	2015	Hong Kong	MCI	4	Cognitive Training	Cognitive and Physical Training	Physical Training	Social Groups	-	2
Lam [25]	2010	Hong Kong	Dementia	2	Case Management	Control	-	-	-	2
Langoni [80]	2019	Brazil	MCI	2	Group Exercise	Control	-	-	-	14
Law [81]	2014	Hong Kong	MCI	2	Functional Tasks Exercise Programme	Cognitive Training	-	-	-	7
Lazarou [82]	2017	Greece	MCI	2	Ballroom Dancing	Control	-	-	-	5
Li [83]	2019	China	MCI	2	Computerised Cognitive Training	Control	-	-	-	4
Lim [84]	2018	Singapore	MCI	2	Mindfulness	Health Education	-	-	-	5
Logsdon [26]	2010	USA	Dementia	2	Early Stage Memory Loss Support Group	Control	-	-	-	10
Luijpen [85]	2005	The Netherlands	MCI	2	TENs	Sham TENS	-	-	-	6
Maffei [86]	2017	Italy	MCI	2	Multidomain Training	Control	-	-	-	10
Manav [87]	2019	Turkey	Dementia	2	Reminiscence Therapy	Social Interview	-	-	-	6
Melendez [88]	2015	Spain	Both	2	Reminiscence Therapy	Control	-	-	-	6
Nagamatsu [89]	2012	Canada	MCI	2	Aerobic Exercise	Resistance Training	-	-	-	13
Olsen [90]	2016	Norway	Both	2	Animal Assisted Therapy	Control	-	-	-	9
Pantoni [91]	2017	Italy	MCI	2	Attention Process Training	Control	-	-	-	4
Park [92]	2018	South Korea	MCI	2	Cognition specific computer training	Non-specific computer training	-	-	-	5
Poinsatte [93]	2019	USA	MCI	2	Aerobic Exercise	Stretching	-	-	-	3
Pongan [94]	2017	France	Dementia	2	Choral Singing	Painting	-	-	-	14
Poptsi [95]	2018	Greece	MCI	5	Paper Language Tasks	Computer Language Tasks	Oral Language Tasks	Active Control	Control	4
Qi [96]	2019	China	MCI	2	Aerobic Exercise	Control	-	-	-	3
Rapp [97]	2002	USA	MCI	2	Memory Enhancement Training (Multi-Component)	Control	-	-	-	9
Rojas [98]	2013	Argentina	MCI	2	Cognitive Intervention	Control	-	-	-	8
Rozzini [99]	2007	Italy	MCI	2	Cognitive Training and AChEIs	AChEIs	-	-	-	7
Savulich [100]	2017	UK	MCI	2	Cognitive Training	Control	-	-	-	9

For peer review only - http://bmjopen.bmj.com/site/about/guidelines.xhtml

Scherder [101]	2010	The Netherlands	MCI	3	Walking	Hand and Face Exercises	Control	-	-	11
Shimada [102]	2017	Japan	MCI	2	Physical and Cognitive Training	Health Education Group	-	-	-	7
Shimizu [103]	2017	Japan	MCI	2	Movement Music Therapy	Single Training Task	-	-	-	4
Simon [104]	2018	Brazil	MCI	2	Memory Training	Active Control	-	-	-	8
Song [105]	2019	China	MCI	2	Aerobic Exercise	Active Control	-	-	-	4
Suzuki [106]	2012	Japan	MCI	2	Multicomponent Exercise Group	Active Control	-	-	-	6
Tappen [27]	2014	USA	Both	2	Cognitive Training (Home Based)	Life Story Interview	-	-	-	11
Troyer [107]	2008	Canada	MCI	2	Multicomponent Intervention	Control	-	-	-	6
Tsai [108]	2018	Taiwan	MCI	3	Aerobic Exercise	Resistance Training	Control	-	-	7
Tsantali [109]	2017	Greece	Dementia	3	Cognitive Training	Cognitive Stimulation	Control	-	-	5
van Uffelen [110]	2007	The Netherlands	MCI	4	Walking	Placebo Activity	Folic Acid/ Vitamin B Supplements	Placebo Pills	-	3
Waldorff [28]	2012	Denmark	Dementia	2	Multifaceted Counselling, Education and Support	Control	-	-	-	2
Wei [111]	2014	China	MCI	2	Handball Training	Control	-	-	-	8
Yang [112]	2016	USA	MCI	2	Memory Enhancement Training	Yoga	-	-	-	3
Yoon [113]	2017	South Korea	MCI	2	High-Speed Power Strength Training	Low-Speed Strength Training	-	-	-	5
Young [114]	2014	Hong Kong	Dementia	2	Support Groups	Control	-	-	-	4
Young [115]	2017	Hong Kong	MCI	2	Holistic Health Group	Control	-	-	-	4
Yun [116]	2016	South Korea	MCI	2	TDS	Sham TDS	-	-	-	1
Zhao [117]	2018	China	MCI	2	Creative Expression Therapy	Cognitive Training	-	-	-	7
Zhu [118]	2018	China	MCI	2	Dance	Control	-	-	-	7

For peer review only - http://bmjopen.bmj.com/site/about/guidelines.xhtml

Table 2. Outcome measures by domain and subdomains

**Person living with dementia measures**<br>**Domain and subdomain**	**Outcome Measure**	**N**
**Cognition/Memory**		**219**
Cognition	MMSE	37
	Trail Making Test	27
	Digit Span Test	12
	ADAS-Cog	10
	Rey Auditory Test	9
	Rivermead Behavioural Memory Test	9
	Stroop Test	7
	MMQ	7
	Novelli Lexical Test	7
	MoCA	6
	CDR	6
	Verbal Fluency	6
	CERAD-NB	5
	Addenbrooke's Cognitive Examination	4
	Boston Naming Test	4
	Rey Osterrieth Complex Figure Task	4
	Montreal Cognitive Test	3
	Attentional Matrices Test	3
	California Verbal Learning Test	3
	Digit Symbol Coding Test	3
	Hopkins Verbal Learning Test	3
	The Wechsler Memory Scale	3
	CAMcog	2
	Cognitive Failures Test	2
	Color Trails Test	2
	Dementia Rating Scale-2	2
	DSM IV Test	2
	Auditory Verbal Learning Test	2
	Corsi's Block Tapping Test	2
	Frontal Assessment Test	2
	Fuld Object Memory Evaluation	2
	Logical Memory (Subtest of Wechsler Memory Scale)	2
	Prospective and Retrospective Memory Questionnaire	2
	Pyramids & Palm Trees	2
	Questionnaire d'Auto Evaluation de la Memoire	2
	Raven's Coloured Matrices	2
	Repeatable Battery Test	2
	The verbal learning and memory test	2
	Visual Memory Span	2
	Wechsler Adult Intelligence Scale	2
Knowledge of Memory Strategies	Memory Strategy Toolbox	2
	Strategy Knowledge Repertoire	1
Attention	Test of Everyday Attention	2
**Behavioural and Psychological Symptoms of dementia**		**51**
Anxiety/Depression	Geriatric Depression Scale*	21
	Cornell Scale for Depression in Dementia*	7

For peer review only - http://bmjopen.bmj.com/site/about/guidelines.xhtml

	Hospital Anxiety and Depression Scale	4
	Beck Depression Inventory	1
Other	Neuropsychiatric Inventory*	12
	Apathy Evaluation Scale	3
	Revised Memory and behaviour problem checklist*	
**Everyday Living**		**20**
Activities of daily Living	Instrumental Activities of Daily Living*	8
	Bayer Activities of Daily Living Scale	3
	Alzheimer's Disease Cooperative Study Activities of Daily Living Scale	2
	Barthel index	2
Functional Ability	Functional Activities Questionnaire	3
	Functional and Cognitive Assessment Test and Functional Rating Scale for Dementia	2
**Physical Outcomes**		**19**
Physical Performance	Timed Up and Go Test	7
	Gait	3
	Handgrip strength	3
	Stride	2
	Walking Speed	2
Physical Measures	Weight	2
**Quality of Life/Wellbeing**		**15**
Quality of Life	QoL in Alzheimer's Disease*	7
	Dementia Quality of Life Instrument*	3
	EuroQoL EQ 5D*	2
	EQ-VAS	1
Stress	Perceived Stress Scale	1
General Wellbeing	SF-36	1
**Biological Outcome**		**9**
Brain Activity	EEG	4
	MRI	2
Biomarker	BDNF	3
**Adherence to Intervention**		**2**
Adherence to intervention	Adherence	2

Caregiver Measures

**Domain**	**Outcome Measure**	**N**
**Depression**		**5**
	The Center for Epidemiological Studies Depression Scale*	3
	Geriatric Depression Scale	1
	Beck Depression Inventory	1
**Caregiver Burden**		**2**
	Zarit caregiver burden interview*	2
**General Wellbeing**		**1**
	Short Form Health Survey (SF-36)*	1
**Knowledge of Memory Strategies**		**1**
	Strategy Knowledge Repertoire	1
**Quality of Life**		**1**
	EQ-VAS	1

For peer review only - http://bmjopen.bmj.com/site/about/guidelines.xhtml

For peer review only

**Stress**		**1**
	Perceived Stress Scale	1

*Measure recommended by INTERDEM Consensus [14]

For peer review only

Table 3. Outcome measure domain by diagnosis and intervention

	Number of Studies	BPSD	Biological Outcome	Caregiver Measure	Cognition/ Memory	Everyday Living	Physical Measures	Physical Performance	Quality of Life/ Wellbeing	Task Performance
**Diagnosis**										
Both	6	5	-	1	12	1	-	-	-	-
Dementia	14	16	-	7	42	6	-	-	6	-
MCI	71	30	9	3	163	12	2	17	9	2
**Type of Intervention**										
Animal Assisted Therapy	2	2	-	-	2	1	-	-	-	-
Art-Based Therapy	2	1	-	1	6	1	-	-	-	-
Case Management	2	2	-	3	1	-	-	-	1	-
Chinese Calligraphy	2	1	1	-	4	-	-	-	-	-
Cognitive Training	37	23	2	3	103	11	-	1	6	2
Cognitive Training and Physical Activity	4	-	-	-	14	2	-	2	-	-
Multicomponent Psychosocial Intervention	4	6	-	3	10	2	-	2	3	-
Music Based Intervention	2	1	-	-	7	-	1	2	1	-
Physical Activity	25	11	6	-	53	3	1	10	2	-
Reminiscence Therapy	2	1	-	-	2	-	-	-	-	-
Support Group	3	3	-	1	1	-	-	-	1	-

For peer review only - http://bmjopen.bmj.com/site/about/guidelines.xhtml

Table 4. Outcome measure domain by country

Country	Number of studies	BPSD	Biological Outcome	Caregiver Measure	Cognition/Memory	Functional ability	Physical Measures	Physical Performance	Quality of Life/ Wellbeing	Task Performance
**Argentina**	1	1	0	0	6	1	0	0	0	0
**Australia**	4	0	0	1	5	1	0	0	0	0
**Brazil**	5	1	1	0	14	0	0	1	0	0
**Canada**	6	2	0	0	16	0	0	2	0	0
**Mainland China, Hong Kong and Taiwan**	20	10	5	1	35	2	0	0	0	1
**Czech Republic**	3	0	0	0	3	2	0	1	0	0
**Denmark**	1	2	0	2	1	1	0	0	2	0
**Finland**	1	1	0	1	3	1	0	0	1	0
**France**	3	1	0	0	6	0	0	2	1	0
**Germany**	4	1	0	0	10	0	0	0	1	0
**Greece**	4	3	0	0	18	2	0	0	1	0
**Hungary**	1	0	0	0	0	0	0	1	0	0
**Iran**	3	1	1	0	3	0	1	0	0	0
**Italy**	11	8	0	0	32	6	0	0	1	0
**Japan**	8	2	0	1	16	1	1	6	0	0
**Norway**	1	1	0	0	1	0	0	0	0	0
**Pakistan**	1	0	1	0	3	0	0	0	0	0
**Singapore**	1	0	0	0	0	0	0	0	0	0
**South Korea**	8	5	0	0	14	1	0	4	3	0
**Spain**	3	2	0	0	2	0	0	0	0	0
**The Netherlands**	5	0	0	2	10	0	0	0	2	0
**Turkey**	1	1	0	0	1	0	0	0	0	0
**UK**	1	3	0	0	1	0	0	0	0	0
**USA**	10	6	1	3	19	2	0	0	3	1

For peer review only - http://bmjopen.bmj.com/site/about/guidelines.xhtml

Figure 1. Flow Chart of Included Studies

Figure 2. Trends in outcome measures over time

Note: QoL = quality of life; BPSD = behavioural and psychological symptoms of dementia

For peer review only

For peer review only - http://bmjopen.bmj.com/site/about/guidelines.xhtml

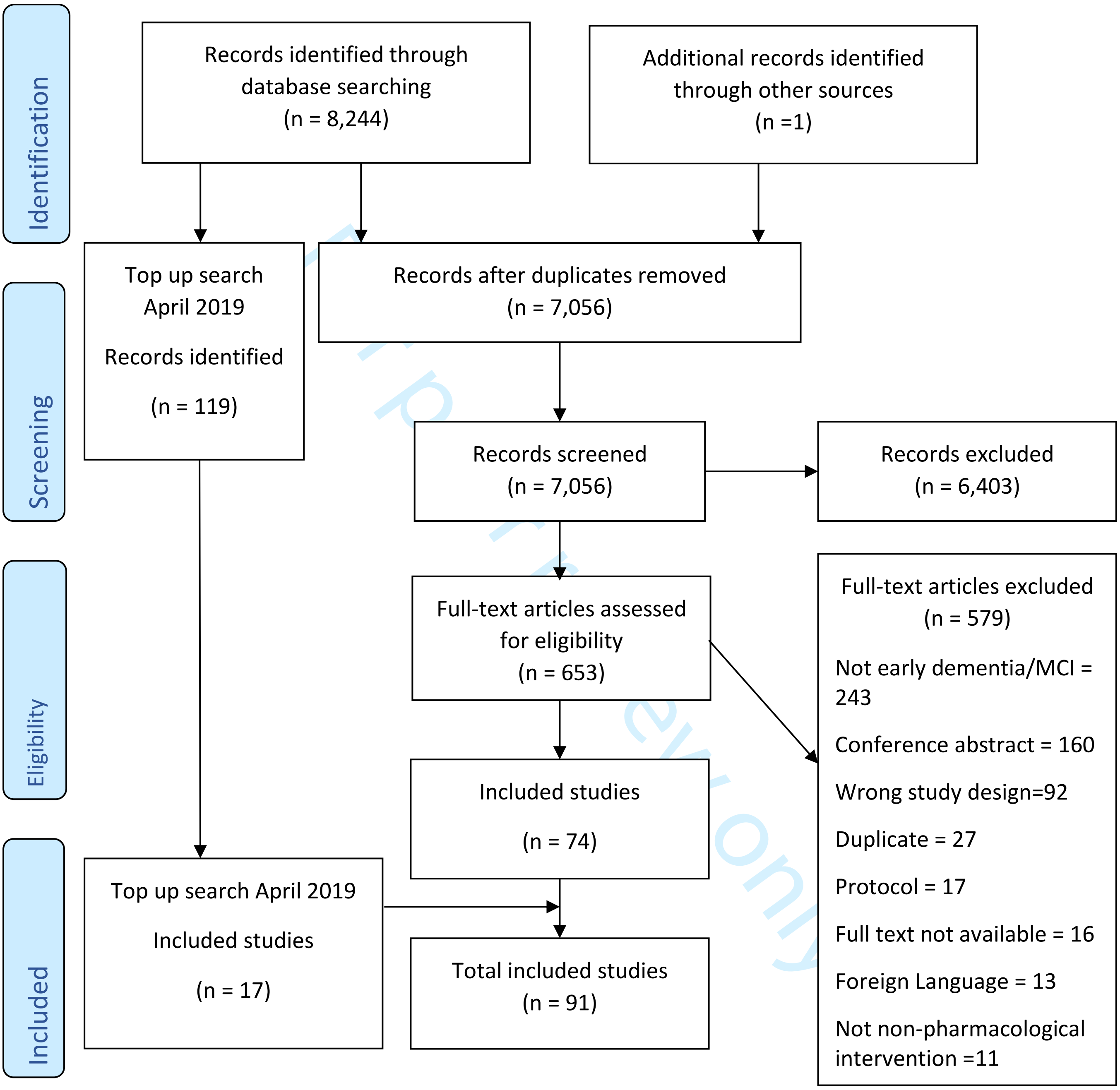

For peer review only

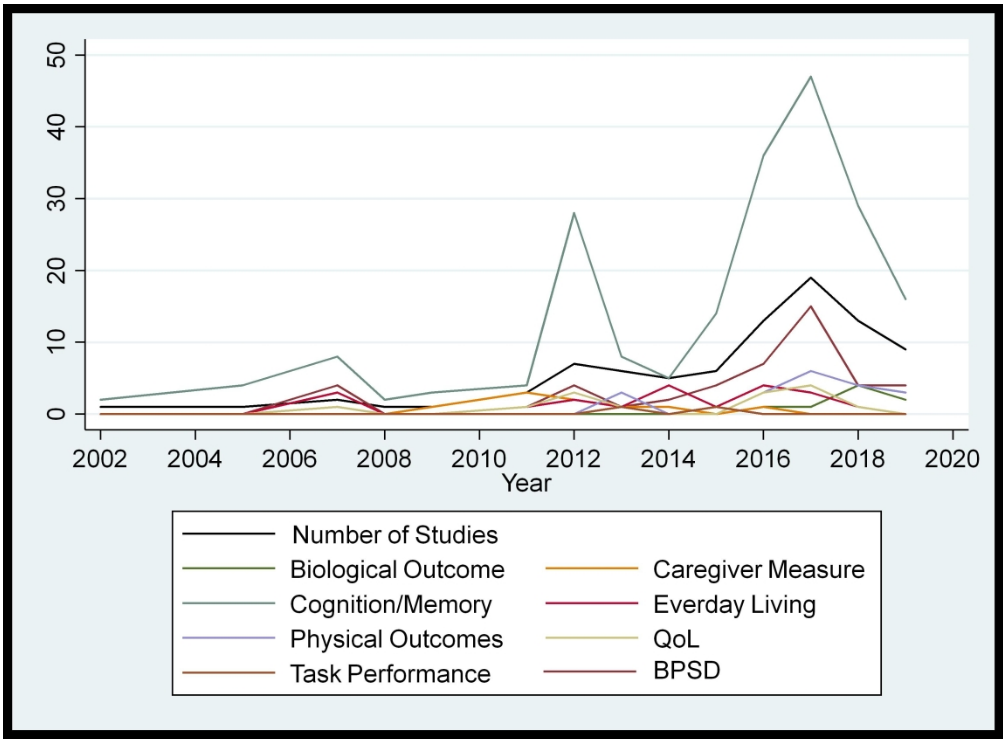

Figure 2. outcome measures over time
Note: QoL = quality of life; BPSD = behavioural and psychological symptoms of dementia.

887x651mm (96 x 96 DPI)

Supplementary Table 1. Search Strategy for OVID

	Search term		Search term continued
**1**	Early dementia	**39**	self help group
**2**	Mild dementia	**40**	psychotherapy
**3**	mild alzheimer*	**41**	CBT
**4**	early alzheimer*	**42**	Cognitive behavio?ral therap*
**5**	cognitive impairment	**43**	Cognitive behavioural therap*
**6**	age related cognitive impairment	**44**	Talking therap*
**7**	Mild cognitive impairment	**45**	Individual therap*
**8**	MCI	**46**	Peer support
**9**	mild neurocognitive disorder	**47**	Counselling
**10**	**1 OR 2 OR 3 OR 4 OR 5 OR 6 OR 7 OR 8 OR 9**	**48**	Communication
**11**	cognitive training	**49**	acupuncture therap*
**12**	brain training	**50**	acupuncture
**13**	memory training	**51**	acupuncture points
**14**	Behavio?r therap*	**52**	Transcranial Magnetic Stimulation
**15**	Behavio?r modification	**53**	TMS
**16**	pleasant activit*	**54**	Relaxation therap*
**17**	Cognitive stimulation therapy	**55**	Therap* relaxation
**18**	CST	**56**	Relaxation techniques
**19**	Transcutaneous Electrical Nerve Stimulation	**57**	Early intervention
**20**	TENS	**58**	Alternative therap*
**21**	Exercise	**59**	**11 OR 12 OR 13 OR 14 OR 15 OR 16 OR 17 OR 18 OR 19 OR 20 OR 21 OR 22 OR 23 OR 24 OR 25 OR 26 OR 27 OR 28 OR 29 OR 30 OR 31 OR 32 OR 33 OR 34 OR 35 OR 36 OR 37 OR 38 OR 39 OR 40 OR 41 OR 42 OR 43 OR 44 OR 45 OR 46 OR 47 OR 48 OR 49 OR 50 OR 51 OR 52 OR 53 OR 54 OR 55 OR 56 OR 57 OR 58 OR 59**
**22**	exercise therap*	**60**	randomized controlled trial
**23**	Walking	**61**	randomised controlled trial

For peer review only

**24**	music therap*	**62**	RCT
**15**	reminiscence therap*	**63**	Clinical Trial
**26**	massage therap*	**64**	intervention
**27**	therap* touch	**65**	**60 OR 61 OR 62 OR 63 OR 64 OR 65**
**28**	recreation therap*	**66**	early dementia
**29**	light therap*	**67**	mild dementia
**30**	therap* light	**68**	mild alzheimer*
**31**	sensory stimulation	**69**	early alzheimer*
**32**	multisensory stimulation	**70**	cognitive impairment
**33**	complementary therap*	**71**	age related cognitive impairment
**34**	aromatherapy	**72**	Mild cognitive impairment
**35**	support group	**73**	MCI
**36**	therap* group	**74**	mild neurocognitive disorder
**37**	memory group	**75**	**66 OR 67 OR 68 OR 69 OR 70 OR 71 OR 72 OR 73 OR 74 OR 75**
**38**	self help	**76**	**10 AND 59 AND 75**

For peer review only

Preferred Reporting Items for Systematic reviews and Meta-Analyses extension for Scoping Reviews (PRISMA-ScR) Checklist

SECTION	ITEM	PRISMA-ScR CHECKLIST ITEM	REPORTED ON PAGE #
**TITLE**			
Title	1	Identify the report as a scoping review.	1
**ABSTRACT**			
Structured summary	2	Provide a structured summary that includes (as applicable): background, objectives, eligibility criteria, sources of evidence, charting methods, results, and conclusions that relate to the review questions and objectives.	2
**INTRODUCTION**			
Rationale	3	Describe the rationale for the review in the context of what is already known. Explain why the review questions/objectives lend themselves to a scoping review approach.	5-6
Objectives	4	Provide an explicit statement of the questions and objectives being addressed with reference to their key elements (e.g., population or participants, concepts, and context) or other relevant key elements used to conceptualize the review questions and/or objectives.	6
**METHODS**			
Protocol and registration	5	Indicate whether a review protocol exists; state if and where it can be accessed (e.g., a Web address); and if available, provide registration information, including the registration number.	6
Eligibility criteria	6	Specify characteristics of the sources of evidence used as eligibility criteria (e.g., years considered, language, and publication status), and provide a rationale.	7-8
Information sources*	7	Describe all information sources in the search (e.g., databases with dates of coverage and contact with authors to identify additional sources), as well as the date the most recent search was executed.	8
Search	8	Present the full electronic search strategy for at least 1 database, including any limits used, such that it could be repeated.	Table 1
Selection of sources of evidence†	9	State the process for selecting sources of evidence (i.e., screening and eligibility) included in the scoping review.	8-9
Data charting process‡	10	Describe the methods of charting data from the included sources of evidence (e.g., calibrated forms or forms that have been tested by the team before their use, and whether data charting was done independently or in duplicate) and any processes for obtaining and confirming data from investigators.	9
Data items	11	List and define all variables for which data were sought and any assumptions and simplifications made.	9
Critical appraisal of individual sources of evidence§	12	If done, provide a rationale for conducting a critical appraisal of included sources of evidence; describe the methods used and how this information was used in any data synthesis (if appropriate).	N/A
Synthesis of results	13	Describe the methods of handling and summarizing the data that were charted.	9

For peer review only

For peer review only - http://bmjopen.bmj.com/site/about/guidelines.xhtml

SECTION	ITEM	PRISMA-ScR CHECKLIST ITEM	REPORTED ON PAGE #
**RESULTS**			
Selection of sources of evidence	14	Give numbers of sources of evidence screened, assessed for eligibility, and included in the review, with reasons for exclusions at each stage, ideally using a flow diagram.	9-10 and Figure 1
Characteristics of sources of evidence	15	For each source of evidence, present characteristics for which data were charted and provide the citations.	10 and Table 2
Critical appraisal within sources of evidence	16	If done, present data on critical appraisal of included sources of evidence (see item 12).	N/A
Results of individual sources of evidence	17	For each included source of evidence, present the relevant data that were charted that relate to the review questions and objectives.	Not feasible
Synthesis of results	18	Summarize and/or present the charting results as they relate to the review questions and objectives.	10-13
**DISCUSSION**			
Summary of evidence	19	Summarize the main results (including an overview of concepts, themes, and types of evidence available), link to the review questions and objectives, and consider the relevance to key groups.	13-14
Limitations	20	Discuss the limitations of the scoping review process.	16-17
Conclusions	21	Provide a general interpretation of the results with respect to the review questions and objectives, as well as potential implications and/or next steps.	17
**FUNDING**			
Funding	22	Describe sources of funding for the included sources of evidence, as well as sources of funding for the scoping review. Describe the role of the funders of the scoping review.	18

JBI = Joanna Briggs Institute; PRISMA-ScR = Preferred Reporting Items for Systematic reviews and Meta-Analyses extension for Scoping Reviews.

* Where *sources of evidence* (see second footnote) are compiled from, such as bibliographic databases, social media platforms, and Web sites.

† A more inclusive/heterogeneous term used to account for the different types of evidence or data sources (e.g., quantitative and/or qualitative research, expert opinion, and policy documents) that may be eligible in a scoping review as opposed to only studies. This is not to be confused with *information sources* (see first footnote).

‡ The frameworks by Arksey and O'Malley (6) and Levac and colleagues (7) and the JBI guidance (4, 5) refer to the process of data extraction in a scoping review as data charting.

§ The process of systematically examining research evidence to assess its validity, results, and relevance before using it to inform a decision. This term is used for items 12 and 19 instead of "risk of bias" (which is more applicable to systematic reviews of interventions) to include and acknowledge the various sources of evidence that may be used in a scoping review (e.g., quantitative and/or qualitative research, expert opinion, and policy document).

From: Tricco AC, Lillie E, Zarin W, O'Brien KK, Colquhoun H, Levac D, et al. PRISMA Extension for Scoping Reviews (PRISMA-ScR): Checklist and Explanation. Ann Intern Med. ;169:467–473. doi: 10.7326/M18-0850

For peer review only - http://bmjopen.bmj.com/site/about/guidelines.xhtml

BMJ Open

Outcomes tested in non-pharmacological interventions in mild cognitive impairment and mild dementia: a scoping review

Journal:	*BMJ Open*
Manuscript ID	bmjopen-2019-035980.R2
Article Type:	Original research
Date Submitted by the Author:	05-Feb-2020
Complete List of Authors:	Couch, Elyse; King's College London, Health Service and Population Research<br>Lawrence, Vanessa; King's College London, Health Service and Population Research<br>Co, Melissa; King's College London, Health Service and Population Research<br>Prina, A.Matthew; King's College London, Health Service and Population Research
**Primary Subject Heading**:	Mental health
Secondary Subject Heading:	Geriatric medicine
Keywords:	Dementia < NEUROLOGY, Old age psychiatry < PSYCHIATRY, STATISTICS & RESEARCH METHODS

SCHOLARONE™
Manuscripts

I, the Submitting Author has the right to grant and does grant on behalf of all authors of the Work (as defined in the below author licence), an exclusive licence and/or a non-exclusive licence for contributions from authors who are: i) UK Crown employees; ii) where BMJ has agreed a CC-BY licence shall apply, and/or iii) in accordance with the terms applicable for US Federal Government officers or employees acting as part of their official duties; on a worldwide, perpetual, irrevocable, royalty-free basis to BMJ Publishing Group Ltd ("BMJ") its licensees and where the relevant Journal is co-owned by BMJ to the co-owners of the Journal, to publish the Work in this journal and any other BMJ products and to exploit all rights, as set out in our licence.

The Submitting Author accepts and understands that any supply made under these terms is made by BMJ to the Submitting Author unless you are acting as an employee on behalf of your employer or a postgraduate student of an affiliated institution which is paying any applicable article publishing charge ("APC") for Open Access articles. Where the Submitting Author wishes to make the Work available on an Open Access basis (and intends to pay the relevant APC), the terms of reuse of such Open Access shall be governed by a Creative Commons licence – details of these licences and which Creative Commons licence will apply to this Work are set out in our licence referred to above.

Other than as permitted in any relevant BMJ Author's Self Archiving Policies, I confirm this Work has not been accepted for publication elsewhere, is not being considered for publication elsewhere and does not duplicate material already published. I confirm all authors consent to publication of this Work and authorise the granting of this licence.

For peer review only

Outcomes tested in non-pharmacological interventions in mild cognitive impairment and mild dementia: a scoping review

Elyse Couch[1*], Vanessa Lawrence[1], Melissa Co[1], and A. Matthew Prina[1]

[1] Health Service and Population Research Department, Institute of Psychiatry, Psychology and Neuroscience, King's College London, UK

Elyse.Couch@kcl.ac.uk

Melissa.Co@kcl.ac.uk

Matthew.Prina@kcl.ac.uk

Vanessa.c.Lawrence@kcl.ac.uk

*Corresponding author information: Health Service and Population Research Department, Institute of Psychiatry, Psychology and Neuroscience, King's College London, UK SE5 8AF.

Email: Elyse.Couch@kcl.ac.uk

For peer review only

For peer review only - http://bmjopen.bmj.com/site/about/guidelines.xhtml

Abstract

Objectives: Non-pharmacological treatments are an important aspect of dementia care. A wide range of interventions have been trialled for mild dementia and mild cognitive impairment (MCI). However, the variety of outcome measures used in these trials makes it difficult to make meaningful comparisons. The objective of this study is to map trends in which outcome measures are used in trials of non-pharmacological treatments in MCI and mild dementia.

Design: Scoping Review

Data Sources: EMBASE, Psych Info, Medline and the Cochrane Register of Controlled Trials were searched from inception until February 2018. An additional search was conducted in April 2019

Eligibility: We included RCTs testing non-pharmacological interventions for people diagnosed with MCI or mild dementia. Studies were restricted to full RCTs; observational, feasibility and pilot studies were not included.

Charting Methods: All outcome measures used by included studies were extracted and grouped thematically. Trends in the types of outcome measures used were explored by type of intervention, country and year of publication.

Results: 91 studies were included in this review. We extracted 358 individual outcome measures, of which 78 (22%) were used more than once. Cognitive measures were the most frequently used, with the MMSE being the most popular.

Conclusions: Our findings highlight an inconsistency in the use of outcome measures. Cognition has been prioritised over other domains, despite previous research highlighting the importance of quality of life and caregiver measures. To ensure a robust evidence base, more research is needed to highlight which outcome measures should be used over others.

Protocol Registration**:** The protocol for this study was registered on PROSPERO (ID: CRD42018102649).

For peer review only

For peer review only - http://bmjopen.bmj.com/site/about/guidelines.xhtml

Word count: 4,255

Strengths and Limitations of this study:

- This scoping review has systematically mapped which outcome measures have been used by randomised controlled trials testing non-pharmacological treatments in mild dementia and mild cognitive impairment.
- This review has explored how the use of outcome measures varies by diagnosis, type of intervention, country and year of publication.
- The papers included in this review were limited to full randomised controlled trials, other study designs may be using different types of outcome measures.
- Further research is needed to establish which measures should be used over others.

For peer review only

For peer review only - http://bmjopen.bmj.com/site/about/guidelines.xhtml

Introduction

Delivery of both pharmacological and non-pharmacological treatment in the early stages of dementia has been identified as a global priority [1 2]. Current pharmacological treatments for the cognitive symptoms of dementia have been found to have a greater effect when delivered as early as possible [3]. However, the benefits of delivering non-pharmacological treatments early are less well understood. Non-pharmacological treatments are an important clinical tool for managing dementia as they are more acceptable to some and less prone to side effects, making them a safe alternative to drug treatments [4]. Those diagnosed earlier in the disease have more cognitive abilities available to engage with non-pharmacological treatments and bolster their own methods for coping with the disease [5]. Previous systematic reviews have found non-pharmacological treatments can improve outcomes; however, these reviews were restricted to a small number of outcome measures [6 7].

Mild cognitive impairment (MCI) has been identified as a potential prodrome for dementia, with approximately 10% of people with MCI converting to a diagnosis of dementia per annum [8]. There is an interest in MCI, as a diagnosis of MCI can facilitate an early diagnosis of dementia and therefore earlier access to dementia services and treatment [9]. MCI is a potentially reversible condition, with many people with MCI reverting back to normal levels of cognition [9]. Therefore, it is important treatments are available. However, it is not clear which treatments can reverse MCI or prevent conversion to dementia [3]. No drug treatments for MCI have been found to be effective [10 11] and acetylcholinesterase inhibitors are not recommended, however, there is some limited evidence that non-pharmacological interventions may be beneficial [3 12].

Randomised Controlled Trials (RCTs) testing non-pharmacological treatments in dementia and MCI are becoming more common. However, they are highly heterogeneous in terms of participants recruited, quality of the study and the types of interventions they are testing, making it difficult to establish the effectiveness of one treatment over another [6 12 13]. Compounding these issues is the inconsistent use of outcome measures in this area of work [9 14].

For peer review only - http://bmjopen.bmj.com/site/about/guidelines.xhtml

Systematic reviews have identified possible benefits of non-pharmacological treatment, yet meta-analyses are difficult to conduct due to the variation in outcome measures used by studies and typically yield small to moderate effect sizes [6 7]. It is possible that these small effect sizes are due to the selection of outcome measures which either lack sensitivity or the change following the intervention not being in the area covered by the outcome measure. It is important researchers are clear on which domains their interventions are targeting, and which measures are best able to capture this change [15]. Pharmacological treatments target specific biological pathways underlying the disease; therefore, outcome measures have been chosen to reflect this and typically focus on cognitive and functional decline[16]. Non-pharmacological treatments generally do not target the underlying biological pathway of the disease therefore, outcome measures should theoretically differ between pharmacological and non-pharmacological treatments[17]. However, a review on non-pharmacological approaches to treating found that studies tended to pay little attention to the mechanisms of change underlying the intervention[4]. The expected mechanisms of change should affect which outcomes are used in non-pharmacological treatments for mild dementia and MCI.

In addition to being clear on how change arises in non-pharmacological treatments, there needs to be a more coherent use of outcomes and the measures used to capture these between studies to ensure a broad and robust evidence base [15]. In 2008, the INTERDEM group, a consortium of dementia researchers across Europe, did work to draw a consensus on which outcome measures should be used when evaluating non-pharmacological treatments. They recommended 22 measures across nine domains including quality of life, mood, global functioning, behaviour, daily living skills, caregiver mood, caregiver burden and staff morale [15]. This guidance does not explore outcomes by the stage of the disease. The outcome measures were selected based on their applicability to European research. The utility of outcome measures may vary by culture[16], previous reviews exploring the use of outcome measures in dementia research have not investigated how this differs by country[17].

For peer review only - http://bmjopen.bmj.com/site/about/guidelines.xhtml

It is not understood which outcome measures are currently being used in non-pharmacological treatments for early dementia and MCI. Scoping reviews present the opportunity to map the evidence on a topic [18], unlike a systematic review scoping reviews can be used to summarise the evidence in a heterogeneous body of literature. Therefore, the aim of this scoping review is to map trends in which outcome measures are being used in RCTs for non-pharmacological treatments in MCI and mild dementia.

Objectives

The specific objectives of this scoping review are to:

(1) Chart which outcomes measures have been used to assess the effectiveness of nonpharmacological treatments in mild dementia and MCI
(2) Highlight which types of measures have been used most frequently
(3) Explore whether the outcome measures used differ depending on the type of intervention, study population, and country the research was conducted in.

Methods

Protocol registration

The protocol for this review was developed following the guidelines set out by the Preferred Reporting Items for Systematic Reviews and Meta-Analysis Extension (PRISMA) statement [19] and the PRISMA guidelines for Scoping Reviews (PRISMA-ScR) [18]. The protocol was registered prospectively on PROSPERO (ID: CRD42018102649).

Eligibility criteria

We included RCTs testing non-pharmacological interventions for people diagnosed with MCI or mild dementia. Studies were restricted to full RCTs; observational, feasibility and pilot studies were not included.

For peer review only - http://bmjopen.bmj.com/site/about/guidelines.xhtml

Studies were included if they met the following criteria:

- Testing non-pharmacological interventions. Studies were not excluded if participants were also treated with acetylcholinesterase inhibitors.
- Participants had a diagnosis of MCI or mild dementia, which was either diagnosed in clinical practice, or met standardised diagnostic criteria, such as the International Statistical Classification of Diseases (ICD-10) or The Diagnostic Statistical Manual of Mental Disorders (DSM), The National Institute of Communicative disorders and Stroke and the Alzheimer's Disease and Related Disorders (NINCDS-ADRDA), the International working group on MCI criteria, The Consortium to Establish a Registry for Alzheimer's Disease (CERAD), The National Institute on Aging- Alzheimer's Associating Diagnostic Guidelines for Alzheimer's Disease, the Petersen Criteria; or was defined by a standardised clinical measure, such as scores between 24-18 on the Mini-Mental State Exam (MMSE); scores ≤26 on the Montreal Cognitive Assessment (MoCA), scores between 15-27 on the St Louis University Mental Status (SLUMS), a Clinical Dementia Rating (CDR) score of 1 (for dementia) or 0.5 (for MCI); or a 4 (for dementia) or 3 (for MCI) on the Global Deterioration Scale (GDS). Studies which include a mix of participants with early dementia and MCI were included, however, studies which included healthy participants and participants with dementia at the later stages of the disease were excluded.
- The intervention was targeted for the person living with dementia or MCI. Dyadic interventions, interventions delivered to both the person living with dementia and their caregivers, were included. Interventions delivered solely to caregivers or health care professionals were excluded.
- Participants were living in long term care facilities or the community
- Written in English

Studies were excluded if:

For peer review only - http://bmjopen.bmj.com/site/about/guidelines.xhtml

- Only pharmacological interventions were tested
- The participants were diagnosed with vascular cognitive impairment, young-onset dementia, Parkinson's Disease Dementia, or MCI with Parkinson's Disease
- Participants were living in a psychiatric inpatient or acute hospital setting
- The intervention had the primary aim of treating major depressive disorder
- The study tested palliative care interventions or advanced care planning
- The only outcome measures used were economic outcomes, such as cost-effectiveness etc.

Information sources and search strategy

To identify potentially relevant studies, we searched EMBASE, Psych Info, Medline and the Cochrane Register of Controlled Trials from inception until 22nd February 2018. An additional search was conducted on 2nd April 2019. See **Supplementary Table 1** for the final search strategy for MEDLINE, which was adapted for the other databases. The final search results were exported into EndNote where duplicates were removed.

Additional papers were identified by searching the references of included papers and other systematic reviews. Conference abstracts and publications were not included.

Selection of sources of evidence

Study selection was managed in Rayyan, where citations were screened against the inclusion and exclusion criteria. Rayyan is an online app for systematic reviews which allows researchers to create their own coding system for decision making [20]. References were first screened by title and abstract, followed by a full-text screening. A second reviewer (MC) screened 10% of the articles at each stage of the review. Disagreements were resolved by discussions with a third reviewer (MP).

A critical appraisal or assessment of the risk of bias is not necessary for a scoping review [18]. This scoping review is not aiming to critically appraise the cumulative literature of outcome measures for

For peer review only - http://bmjopen.bmj.com/site/about/guidelines.xhtml

non-pharmacological treatment in MCI and mild dementia, therefore we did not conduct a critical appraisal or risk of bias assessment for this review.

Data charting process and data items

Data from eligible studies were charted using a standardised extraction tool designed for this study. Items deemed most relevant to the review objectives were the diagnosis of the study participants, description of interventions being tested, the number of intervention groups, and outcome measures used with references.

Synthesis of results

The charted data were mapped to reflect the objectives of this review. Following data charting, outcome measures which were used more than once across the included studies were grouped by domain. We grouped the interventions thematically by the type of intervention being tested.

We explored which types of outcome measures were used by intervention type, by tabulating the type of intervention against the domain of the outcome measure. We excluded interventions which were only used once from this summary. Results were presented in tables and summarised narratively.

Patient and Participant Involvement

The South London and Maudsley MALADY group, of current and former carers of people living with dementia, were consulted in the planning of this study.

Results

Included studies

After duplicates were removed, a total of 7,056 citations were screened for inclusion, 653 were screened at full text and 76 papers were initially identified. A top-up search in April 2019 identified 119 new citations, 17 were included making the total number of included studies 91, See **Figure 1**.

For peer review only - http://bmjopen.bmj.com/site/about/guidelines.xhtml

The studies included in this review are described in **Table 1**, including diagnosis of included participants, number of intervention groups, details on the interventions and comparisons tested and the number of outcomes measures used. The included studies were published between 2002 and 2019.

The majority of studies included in this review were conducted in the USA (n=10), Hong Kong (n=10), and Italy (n=10). Followed by mainland China (n=7), Japan (n=7), and South Korea (n=7). Studies were also conducted in: Argentina, Australia, Brazil, Canada, Czech Republic, Denmark, France, Finland, Germany, Greece, Hungary, Iran, Norway, Pakistan, Singapore, Spain, Taiwan, The Netherlands, Turkey, and the United Kingdom; these countries had fewer than 5 included studies each.

Most studies only recruited participants with MCI (n=72), followed by mild dementia only (n=15), and six studies recruited both participants with MCI and mild dementia.

Results of individual sources of evidence

We extracted 358 individual outcome measures from the included studies, of these 78 (22%) were used more than once. Out of the 78 measures used more than once, 70 (88%) were measures of participants living with dementia (PLWD), 6 measures were used in both the PLWD and their caregiver, 2 measures were only of the caregiver. The number of outcome measures used by each study ranged between one and 21 with an average of 6.85.

Types of non-pharmacological interventions

We grouped the interventions thematically by type. The most frequently tested type of intervention was cognitive training (n=36) followed by physical activity (n=25), combined physical activity and cognitive training (n=4), multicomponent psychosocial interventions (n=4) and support groups (n=3). Animal-assisted therapies, art-based therapies, case management, Chinese calligraphy, music-based interventions and reminiscence therapy were each tested in two studies.

For peer review only - http://bmjopen.bmj.com/site/about/guidelines.xhtml

For peer review only

A group weight loss programme, mindfulness, social activities, transcranial direct current stimulation (TDS), transcutaneous electrical nerve stimulation (TENs), and Transcranial magnetic stimulation (TMS) were each trialled once. These interventions were not included in the analysis of trends in outcome measures.

PLWD outcome measures

Table 2 presents the PLWD specific outcome measures grouped by domain. The most frequently measured domain in PLWD was cognition/memory, which was measured 219 times across the 93 included studies. The most frequent measure of cognition was the MMSE, which was measured 37 times. In addition to measures of memory performance knowledge of memory strategies was measured 3 times in PLWD.

The next most frequently measured domain in PLWD was behavioural and psychological symptoms of dementia (BPSD), within this depression was the most commonly measured BPSD. The Geriatric Depression Scale was the most used measure in this domain, followed by the Neuropsychiatric Inventory which examines a greater number of symptoms. Other BSPDs measured were apathy and agitation resulting from memory problems.

Quality of life and wellbeing were measured 15 times across the included study. Quality of life was measured 15 times using four different instruments, the most popular of which was Logsdon's Quality of Life in Alzheimer's disease scale which was used seven times.

Measures of everyday living, physical ability, biological outcomes and adherence to the intervention delivered in the study were measured less than 20 times across the included studies.

Caregiver measures

Eight interventions in this study were dyadic [21-28], all included outcome measures specific to the caregiver in addition to the PLWD. One study of an intervention solely delivered to the PLWD also included a caregiver specific measure [29].

For peer review only - http://bmjopen.bmj.com/site/about/guidelines.xhtml

Table 2 also presents the outcome measures administered to caregivers grouped by domain. The Center for Epidemiological Studies Depression Scale and the Zarit Caregiver Burden interview were the only measures which were administered solely to caregivers. The other caregiver measures were also administered to PLWD. The most frequently measured domain in caregivers was depression, followed by caregiver burden. General wellbeing, knowledge of memory strategies, quality of life and stress were each measured once.

Use of outcome measures over time

Randomised controlled trials of non-pharmacological treatments in mild dementia and MCI have become more frequent over recent years. Almost half (48%) of studies included in this review were published between 2016 and 2018.

Figure 2 charts trends in outcome measure domains over time. As the number of studies in this area has increased over time, so too has the use of outcome measures in all domains. Cognition/memory has consistently been measured over other domains from the beginning of this sample. The only noticeable trend change is in measures of BPSD, which was generally in line with other domains until around 2012, when it overtakes other domains.

Nearly all studies in 2014 included a measure of everyday living; however, since then, the number of studies including these measures has declined. Where measures of everyday living are being used less, measures of BPSD are being used more.

Similarly, caregiver measures were consistently used until 2011, when in 2010 and 2011 all studies included a caregiver measure, however since then the use of such measures has declined.

Use of outcome measures by intervention

Table 3 presents diagnosis and type of intervention by the domains measured. Cognition/memory was the most measured domain across all diagnostic groups, followed by BPSD. The third most

For peer review only

common domain for MCI studies was physical performance, whereas caregiver measures were the third most common type of measures used in studies of early dementia,

Cognition/memory was measured in all types of intervention. Measures of BPSD were most common in cognitive training interventions and physical activity interventions, however, they were not used by combined cognitive and physical training interventions. Quality of life was measured by studies of case management, cognitive training, psychosocial interventions, physical activity and support groups.

Caregiver measures were used in five types of interventions. Case management, cognitive training and psychosocial interventions; followed by arts-based therapy and support groups.

Use of outcome measures by country

Table 4 presents the country the research was conducted in by outcome measure domain. Generally, there was not too much variability in the domain of outcome measures used by country. Cognition/memory was the domain most frequently measured by all countries, followed by BPSD. The majority of studies were conducted in China (including Hong Kong and Taiwan), these studies focused on cognition/memory, BPSD and biological outcome measures. Other than China, only three other countries included biological measures (Iran, Pakistan and the USA). The USA had the second largest number of studies included in this review, these studies favoured cognition/memory, BPSD, caregiver measures and quality of life. Out of the 24 countries with studies included in this review, less than half (n=9) included measures of quality of life.

Discussion

In this study, we used a scoping review to map which outcome measures had been used in trials for non-pharmacological treatments of mild dementia and MCI. We extracted 358 individual outcome measures used in 91 trials, only 22% of which were used more than once. We grouped the outcome measures which had been used more than once and examined differences in their use over time, by

For peer review only - http://bmjopen.bmj.com/site/about/guidelines.xhtml

diagnostic group, country the research was set in and by the type of intervention they were being used to evaluate. Measures of cognition and BPSDs were the most frequently used across all studies and types of intervention.

Perhaps unsurprisingly, measures of cognition or memory are the most prevalent across all countries, diagnostic groups and types of intervention with the MMSE being the most frequently used outcome measure, despite the ADAS-cog having been validated as the gold-standard measure of cognition [15 30 31]. Measuring cognition is central to measuring the progression of dementia and is a clinically and empirically useful outcome to measure in dementia research [31]. However, in this review, we charted 40 different measures of cognition. This indicates that while cognition has been prioritised as an outcome in studies of non-pharmacological interventions, there is no consensus between researchers on which specific measures should be used. In addition to measures of cognitive performance, three studies have also measured participant's knowledge or retention of memory strategies, indicating an interest in longer-term coping strategies for memory loss.

Measures of the BPSD have become more common over time, becoming in 2017 the most measured outcome after cognition. There is not much variety in the BPSDs which have been measured. Generally, depression was measured over other BPSDs. Other BPSDs such as agitation were measured less, perhaps because they are more associated with the later stages of the disease and depression is associated with the earlier stages [32].

Quality of life and wellbeing were not amongst the most measured domains. Four measures of quality of life were used 13 times across the included studies and all but one of these measures were dementia specific measures. It is surprising quality of life has not been measured more, as previous research has stated that in the absence of a cure, health care providers have a greater ability to improve quality of life than alter the progression of the disease [33]. Furthermore, both people with MCI and caregivers rated quality of life of the patient as the most important outcome to measure, followed by caregiver quality of life/burden [34]. Indicating while quality of life has been identified as a

For peer review only - http://bmjopen.bmj.com/site/about/guidelines.xhtml

priority by PLWD, people diagnosed with MCI and their caregivers in previous research, the findings of this study shows this is not being translated into trials of non-pharmacological treatments for early dementia and MCI.

Likewise, caregiver measures had consistent low use across the studies included in this review. We charted eight caregiver measures which were used 11 times across the included studies. Caregiver measures were more commonly used in studies of PLWD, rather than MCI. Previous research has highlighted the profound effect of dementia on their caregivers, with around half of caregivers experiencing high levels of burden [35]. However, a third of caregivers of people with MCI also report extreme levels of burden [36], yet the findings of this study show this is less investigated.

There was great variability in the types of outcomes being used to evaluate the different types of intervention. All studies measured cognition and all but one measured BPSD. A lack of clarity in how change occurs as a result of non-pharmacological treatments is a fundamental weakness in this area of work [4]. It is unlikely that all interventions being tested in this review could hope to improve cognition, however this is the most prevalent domain of outcome measures. There are a number of practical reasons as to why certain outcomes, and therefore outcome measures are used over others, In the past, pharmacological treatments have been required to include some measure of cognition, functional or global assessment[17], it is possible that this approach has influenced the choice in outcomes used in non-pharmacological studies. Furthermore, some measures may be used over others for more practical reasons. For example, measures which are short to administer and free to use may be priorities over others[31]. Several interventions in this review comprise of more than one component, e.g. physical activity and cognitive training. In these cases, it may take multiple measures over many domains to accurately capture change. It is vital that outcome measures are selected depending on the domains the intervention is seeking to address [31].

In 2008, the INTERDEM group recommended 22 outcome measures for use across nine domains [15]. We found 11 of these 22 measures (50%) were used by the studies included in this review, one of

For peer review only - http://bmjopen.bmj.com/site/about/guidelines.xhtml

the recommended domains (staff carer morale) was not applicable to the studies included in this review. All measures recommended for measuring patient mood, and patient quality of life were charted in this review. Only one of the recommended measures for the activities of daily living, caregiver mood, caregiver burden and caregiver quality of life domains were charted and no measures under the global measures domain were charted in this review. This indicates that there is some consistency between which measures are recommended and which measures are utilised, this is largely for patient measures and there is less consistency for caregiver measures.

In this study, we found that the use of outcome measures did not vary much by the country the study was conducted in. In each country, cognition/memory was the most commonly tested domain, followed by BPSD. The importance of outcomes may vary between cultures; therefore, it is important that the outcomes and measures used reflect this [16]. However, due to the limitations of the methodology used we cannot comment on the cultural relevance of the outcome measures charted in this review. Furthermore, articles were only included if they were published in English. It is possible that more culturally appropriate outcomes were used in articles published in the same language as the population under investigation. This is an important area for future research.

Limitations

The findings of this review must be interpreted in the context of the study. To make this review feasible we only included full RCTs, other outcome measures may have been used in different types of studies. Due to time constraints, some sub-types of dementia and cognitive impairment (young-onset, Parkinson's disease dementia and vascular cognitive impairment) were excluded from this review, which limits the applicability of these findings. Further research is needed to explore whether the pattern in the use of outcomes and outcome measures is similar in these groups, compared with the ones included in this review. Furthermore, only outcome measures which were published could be included in this review. The studies included in this study were heterogeneous in terms of participants recruited, interventions tested, and outcome measures used, making it difficult

For peer review only - http://bmjopen.bmj.com/site/about/guidelines.xhtml

to group them thematically. It is possible some nuance is lost in the exploration of broader themes. As with the nature of scoping reviews, we are only able to present which outcome measures have been used in previous research, we are unable to draw conclusions as to which outcome measures should be used over others. Future research should explore which populations measures have been validated for and what constitutes a clinically useful change.

Implications and recommendations for future research

The findings of this review indicate there is very little consistency in outcome measures used in RCTs for non-pharmacological interventions in MCI and mild dementia, however we are not able to conclude which measures should be used over others. To create a strong evidence base for non-pharmacological treatments more research, with the involvement of PLWD and their carers, is needed to determine which measures are preferable over a greater number of domains. Additionally, the prevalence of cognitive measures found in this study, suggests that researchers are including such measures because there is an expectation to do. Researchers should be clear on the theory behind how their intervention creates change and use the appropriate outcome measures.

Conclusions

In summary, this study has found RCTs for non-pharmacological treatments in mild dementia and MCI use a broad range of outcome measures, with a small proportion being used more than once. Excepting measures of cognition, there is very little commonality between studies. Where previous research has set priorities on outcomes preferred by PLWD, people with MCI and caregivers, quality of life for example, this has not yet translated into studies measuring new treatments. Further research is needed to understand which outcomes should be prioritised and how they should be measured.

Contributors

For peer review only - http://bmjopen.bmj.com/site/about/guidelines.xhtml

EC designed the study, carried out the literature review, the data charting and synthesis, data interpretation, article preparation, article review and correspondence. AMP and VL contributed to the study design, data interpretation, and article review. MC contributed to the data charting.

Funding

EC is supported by a studentship from the ESRC LISS-DTP.

Competing interests

None declared

Data availability statement

No additional data available.

For peer review only

References

1. Organization WH. Global action plan on the public health response to dementia 2017–2025. 2017
2. Prince M, Bryce R, Ferri C. World Alzheimer Report 2011: The benefits of early diagnosis and intervention. 2018
3. Livingston G, Sommerlad A, Orgeta V, et al. Dementia prevention, intervention, and care. *The Lancet* 2017;390(10113):2673-734.
4. Douglas S, James I, Ballard C. Non-pharmacological interventions in dementia. *Advances in psychiatric treatment* 2004;10(3):171-77.
5. Kasl-Godley J, Gatz M. Psychosocial interventions for individuals with dementia: an integration of theory, therapy, and a clinical understanding of dementia. *Clinical psychology review* 2000;20(6):755-82.
6. Olazarán J, Reisberg B, Clare L, et al. Nonpharmacological therapies in Alzheimer's disease: a systematic review of efficacy. *Dementia and geriatric cognitive disorders* 2010;30(2):161-78.
7. Brodaty H, Arasaratnam C. Meta-analysis of nonpharmacological interventions for neuropsychiatric symptoms of dementia. *American Journal of Psychiatry* 2012;169(9):946-53.
8. Bruscoli M, Lovestone S. Is MCI really just early dementia? A systematic review of conversion studies. *International Psychogeriatrics* 2004;16(2):129-40.
9. Portet F, Ousset P, Visser P, et al. Mild cognitive impairment (MCI) in medical practice: a critical review of the concept and new diagnostic procedure. Report of the MCI Working Group of the European Consortium on Alzheimer's Disease. *Journal of Neurology, Neurosurgery & Psychiatry* 2006;77(6):714-18.
10. Allain H, Bentué-Ferrer D, Akwa Y. Treatment of the mild cognitive impairment (MCI). *Human Psychopharmacology: Clinical and Experimental* 2007;22(4):189-97.
11. Petersen RC. MCI treatment trials: failure or not? *The Lancet Neurology* 2007;6(6):473-75.
12. Teixeira CVL, Gobbi LTB, Corazza DI, et al. Non-pharmacological interventions on cognitive functions in older people with mild cognitive impairment (MCI). *Archives of gerontology and geriatrics* 2012;54(1):175-80.
13. Harding AJ, Morbey H, Ahmed F, et al. Developing a core outcome set for people living with dementia at home in their neighbourhoods and communities: study protocol for use in the evaluation of non-pharmacological community-based health and social care interventions. *Trials* 2018;19(1):247.
14. Paulino Ramirez Diaz S, Gil Gregório P, Manuel Ribera Casado J, et al. The need for a consensus in the use of assessment tools for Alzheimer's disease: the Feasibility Study (assessment tools for dementia in Alzheimer Centres across Europe), a European Alzheimer's Disease Consortium's (EADC) survey. *International journal of geriatric psychiatry* 2005;20(8):744-48.
15. Moniz-Cook E, Vernooij-Dassen M, Woods R, et al. A European consensus on outcome measures for psychosocial intervention research in dementia care. *Aging and Mental Health* 2008;12(1):14-29.
16. Katona C, Livingston G, Cooper C, et al. International Psychogeriatric Association consensus statement on defining and measuring treatment benefits in dementia. *International psychogeriatrics* 2007;19(3):345-54.
17. Harrison JK, Noel-Storr AH, Demeyere N, et al. Outcomes measures in a decade of dementia and mild cognitive impairment trials. *Alzheimer's research & therapy* 2016;8(1):48.
18. Tricco AC, Lillie E, Zarin W, et al. PRISMA extension for scoping reviews (PRISMA-ScR): checklist and explanation. *Annals of internal medicine* 2018;169(7):467-73.
19. Moher D, Liberati A, Tetzlaff J, et al. Preferred reporting items for systematic reviews and meta-analyses: the PRISMA statement. *Annals of internal medicine* 2009;151(4):264-69.
20. Ouzzani M, Hammady H, Fedorowicz Z, et al. Rayyan—a web and mobile app for systematic reviews. *Systematic reviews* 2016;5(1):210.

For peer review only - http://bmjopen.bmj.com/site/about/guidelines.xhtml

21. Greenaway M, Duncan N, Smith G. The memory support system for mild cognitive impairment: randomized trial of a cognitive rehabilitation intervention. *International journal of geriatric psychiatry* 2013;28(4):402-09.
22. Jansen AP, van Hout HP, Nijpels G, et al. Effectiveness of case management among older adults with early symptoms of dementia and their primary informal caregivers: a randomized clinical trial. *International journal of nursing studies* 2011;48(8):933-43.
23. Kinsella GJ, Mullaly E, Rand E, et al. Early intervention for mild cognitive impairment: a randomised controlled trial. *Journal of Neurology, Neurosurgery & Psychiatry* 2009;80(7):730-36.
24. Koivisto AM, Hallikainen I, Välimäki T, et al. Early psychosocial intervention does not delay institutionalization in persons with mild Alzheimer disease and has impact on neither disease progression nor caregivers' well-being: ALSOVA 3-year follow-up. *International journal of geriatric psychiatry* 2016;31(3):273-83.
25. Lam LC, Lee JS, Chung JC, et al. A randomized controlled trial to examine the effectiveness of case management model for community dwelling older persons with mild dementia in Hong Kong. *International Journal of Geriatric Psychiatry: A journal of the psychiatry of late life and allied sciences* 2010;25(4):395-402.
26. Logsdon RG, Pike KC, McCurry SM, et al. Early-stage memory loss support groups: outcomes from a randomized controlled clinical trial. *Journals of Gerontology Series B: Psychological Sciences and Social Sciences* 2010;65(6):691-97.
27. Tappen RM, Hain D. The effect of in-home cognitive training on functional performance of individuals with mild cognitive impairment and early-stage Alzheimer's disease. *Research in gerontological nursing* 2013;7(1):14-24.
28. Waldorff F, Buss D, Eckermann A, et al. Efficacy of psychosocial intervention in patients with mild Alzheimer's disease: the multicentre, rater blinded, randomised Danish Alzheimer Intervention Study (DAISY). *Bmj* 2012;345:e4693.
29. Hattori H, Hattori C, Hokao C, et al. Controlled study on the cognitive and psychological effect of coloring and drawing in mild Alzheimer's disease patients. *Geriatrics & gerontology international* 2011;11(4):431-37.
30. Rockwood K, Fay S, Gorman M, et al. The clinical meaningfulness of ADAS-Cog changes in Alzheimer's disease patients treated with donepezil in an open-label trial. *BMC neurology* 2007;7(1):26.
31. Sheehan B. Assessment scales in dementia. *Therapeutic advances in neurological disorders* 2012;5(6):349-58.
32. Piccininni M, Di Carlo A, Baldereschi M, et al. Behavioral and psychological symptoms in Alzheimer's disease: frequency and relationship with duration and severity of the disease. *Dementia and geriatric cognitive disorders* 2005;19(5-6):276-81.
33. Brod M, Stewart AL, Sands L, et al. Conceptualization and measurement of quality of life in dementia: the dementia quality of life instrument (DQoL). *The Gerontologist* 1999;39(1):25-36.
34. Barrios PG, González RP, Hanna SM, et al. Priority of treatment outcomes for caregivers and patients with mild cognitive impairment: preliminary analyses. *Neurology and therapy* 2016;5(2):183-92.
35. Schulz R, O'Brien AT, Bookwala J, et al. Psychiatric and physical morbidity effects of dementia caregiving: prevalence, correlates, and causes. *The Gerontologist* 1995;35(6):771-91.
36. Bruce JM, McQuiggan M, Williams V, et al. Burden among spousal and child caregivers of patients with mild cognitive impairment. *Dementia and geriatric cognitive disorders* 2008;25(4):385.
37. Amjad I, Toor H, Niazi IK, et al. Therapeutic effects of aerobic exercise on EEG parameters and higher cognitive functions in mild cognitive impairment patients. *International Journal of Neuroscience* 2019;129(6):551-62.

For peer review only - http://bmjopen.bmj.com/site/about/guidelines.xhtml

38. Bae S, Lee S, Lee S, et al. The effect of a multicomponent intervention to promote community activity on cognitive function in older adults with mild cognitive impairment: A randomized controlled trial. *Complementary therapies in medicine* 2019;42:164-69.
39. Baker LD, Frank LL, Foster-Schubert K, et al. Effects of aerobic exercise on mild cognitive impairment: a controlled trial. *Archives of neurology* 2010;67(1):71-79.
40. Belleville S, Hudon C, Bier N, et al. MEMO+: efficacy, durability and effect of cognitive training and psychosocial intervention in individuals with mild cognitive impairment. *Journal of the American Geriatrics Society* 2018;66(4):655-63.
41. Biasutti M, Mangiacotti A. Assessing a cognitive music training for older participants: a randomised controlled trial. *International journal of geriatric psychiatry* 2018;33(2):271-78.
42. Bono A, Benvenuti C, Buzzi M, et al. Effects of animal assisted therapy (AAT) carried out with dogs on the evolution of mild cognitive impairment. *G Gerontol* 2015;63(1):32-6.
43. Burgio F, Delazer M, Meneghello F, et al. Cognitive training improves ratio processing and decision making in patients with mild cognitive impairment. *Journal of Alzheimer's Disease* 2018(Preprint):1-14.
44. Buschert VC, Teipel SJ, Jolk S, et al. Long-term observation of a multicomponent cognitive intervention in mild cognitive impairment. *The Journal of clinical psychiatry* 2012
45. Carretti B, Borella E, Fostinelli S, et al. Benefits of training working memory in amnestic mild cognitive impairment: specific and transfer effects. *International Psychogeriatrics* 2013;25(4):617-26.
46. Cavallo M, Hunter EM, van der Hiele K, et al. Computerized structured cognitive training in patients affected by early-stage Alzheimer's disease is feasible and effective: a randomized controlled study. *Archives of Clinical Neuropsychology* 2016;31(8):868-76.
47. Chan SC, Lam TL, Fong KN, et al. Generalization of context-specific training in individuals with mild cognitive impairment: an event-related potential study. *Dementia and geriatric cognitive disorders extra* 2016;6(3):568-79.
48. Chan SC, Chan CC, Derbie AY, et al. Chinese calligraphy writing for augmenting attentional control and working memory of older adults at risk of mild cognitive impairment: a randomized controlled trial. *Journal of Alzheimer's Disease* 2017;58(3):735-46.
49. Choi W, Lee S. Ground kayak paddling exercise improves postural balance, muscle performance, and cognitive function in older adults with mild cognitive impairment: a randomized controlled trial. *Medical science monitor: international medical journal of experimental and clinical research* 2018;24:3909.
50. Donnezan LC, Perrot A, Belleville S, et al. Effects of simultaneous aerobic and cognitive training on executive functions, cardiovascular fitness and functional abilities in older adults with mild cognitive impairment. *Mental Health and Physical Activity* 2018;15:78-87.
51. DiNapoli EA, Scogin F, Bryant AN, et al. Effect of individualized social activities on quality of life among older adults with mild to moderate cognitive impairment in a geriatric psychiatry facility. *Aging & mental health* 2016;20(3):262-70.
52. Doi T, Makizako H, Shimada H, et al. Effects of multicomponent exercise on spatial–temporal gait parameters among the elderly with amnestic mild cognitive impairment (aMCI): Preliminary results from a randomized controlled trial (RCT). *Archives of gerontology and geriatrics* 2013;56(1):104-08.
53. Doi T, Verghese J, Makizako H, et al. Effects of cognitive leisure activity on cognition in mild cognitive impairment: results of a randomized controlled trial. *Journal of the American Medical Directors Association* 2017;18(8):686-91.
54. Drumond Marra HL, Myczkowski ML, Maia Memória C, et al. Transcranial magnetic stimulation to address mild cognitive impairment in the elderly: a randomized controlled study. *Behavioural neurology* 2015;2015

For peer review only

55. Emsaki G, NeshatDoost HT, Tavakoli M, et al. Memory specificity training can improve working and prospective memory in amnestic mild cognitive impairment. *Dementia & neuropsychologia* 2017;11(3):255-61.
56. Eyre HA, Siddarth P, Acevedo B, et al. A randomized controlled trial of Kundalini yoga in mild cognitive impairment. *International psychogeriatrics* 2017;29(4):557-67.
57. Feng W, Wang D, Tang L, et al. Effects of Different Cognitive Trainings on Amnestic Mild Cognitive Impairment in the Elderly: A One-Year Longitudinal Functional Magnetic Resonance Imaging (MRI) Study. *Medical science monitor: international medical journal of experimental and clinical research* 2018;24:5517.
58. Fernández-Calvo B, Contador I, Ramos F, et al. Effect of unawareness on rehabilitation outcome in a randomised controlled trial of multicomponent intervention for patients with mild Alzheimer's disease. *Neuropsychological rehabilitation* 2015;25(3):448-77.
59. Singh MAF, Gates N, Saigal N, et al. The Study of Mental and Resistance Training (SMART) study—resistance training and/or cognitive training in mild cognitive impairment: a randomized, double-blind, double-sham controlled trial. *Journal of the American Medical Directors Association* 2014;15(12):873-80.
60. Finn M, McDonald S. Repetition-lag training to improve recollection memory in older people with amnestic mild cognitive impairment. A randomized controlled trial. *Aging, Neuropsychology, and Cognition* 2015;22(2):244-58.
61. Fogarty JN, Murphy KJ, McFarlane B, et al. Taoist Tai Chi® and memory intervention for individuals with mild cognitive impairment. *Journal of aging and physical activity* 2016;24(2):169-80.
62. Förster S, Buschert VC, Teipel SJ, et al. Effects of a 6-month cognitive intervention on brain metabolism in patients with amnestic MCI and mild Alzheimer's disease. *Journal of Alzheimer's Disease* 2011;26(s3):337-48.
63. Galante E, Venturini G, Fiaccadori C. Computer-based cognitive intervention for dementia: preliminary results of a randomized clinical trial. *G Ital Med Lav Ergon* 2007;29(3 Suppl B):B26-32.
64. Hagovská M, Dzvoník O, Olekszyová Z. Comparison of two cognitive training programs with effects on functional activities and quality of life. *Research in gerontological nursing* 2017;10(4):172-80.
65. Hagovska M, Takáč P, Dzvonik O. Effect of a combining cognitive and balanced training on the cognitive, postural and functional status of seniors with a mild cognitive deficit in a randomized, controlled trial. *European journal of physical and rehabilitation medicine* 2016;52(1):101-09.
66. Han JW, Son KL, Byun HJ, et al. Efficacy of the Ubiquitous Spaced Retrieval-based Memory Advancement and Rehabilitation Training (USMART) program among patients with mild cognitive impairment: a randomized controlled crossover trial. *Alzheimer's research & therapy* 2017;9(1):39.
67. Han JW, Lee H, Hong JW, et al. Multimodal cognitive enhancement therapy for patients with mild cognitive impairment and mild dementia: A multi-center, randomized, controlled, double-blind, crossover trial. *Journal of Alzheimer's Disease* 2017;55(2):787-96.
68. Ho RT, Fong TC, Chan WC, et al. Psychophysiological Effects of Dance Movement Therapy and Physical Exercise on Older Adults With Mild Dementia: A Randomized Controlled Trial. *The Journals of Gerontology: Series B*
69. Horie NC, Serrao VT, Simon SS, et al. Cognitive effects of intentional weight loss in elderly obese individuals with mild cognitive impairment. *The Journal of Clinical Endocrinology & Metabolism* 2016;101(3):1104-12.
70. Hyer L, Scott C, Atkinson MM, et al. Cognitive training program to improve working memory in older adults with MCI. *Clinical gerontologist* 2016;39(5):410-27.

For peer review only - http://bmjopen.bmj.com/site/about/guidelines.xhtml

71. Jean L, Simard M, Wiederkehr S, et al. Efficacy of a cognitive training programme for mild cognitive impairment: results of a randomised controlled study. *Neuropsychological rehabilitation* 2010;20(3):377-405.
72. Jelcic N, Cagnin A, Meneghello F, et al. Effects of lexical–semantic treatment on memory in early Alzheimer disease: An observer-blinded randomized controlled trial. *Neurorehabilitation and neural repair* 2012;26(8):949-56.
73. Jeong JH, Na HR, Choi SH, et al. Group-and home-based cognitive intervention for patients with mild cognitive impairment: a randomized controlled trial. *Psychotherapy and psychosomatics* 2016;85(4):198-207.
74. Kohanpour MA, Peeri M, Azarbayjani MA. The effects of aerobic exercise with lavender essence use on cognitive state and serum brain-derived neurotrophic factor levels in elderly with mild cognitive impairment. *Journal of herbmed pharmacology* 2017;6
75. Kovacs E, Sztruhar Jonasne I, Karoczi C, et al. Effects of a multimodal exercise program on balance, functional mobility and fall risk in older adults with cognitive impairment: a randomized controlled single-blind study. *Eur J Phys Rehabil Med* 2013;49(5):639-48.
76. Küster OC, Fissler P, Laptinskaya D, et al. Cognitive change is more positively associated with an active lifestyle than with training interventions in older adults at risk of dementia: a controlled interventional clinical trial. *BMC psychiatry* 2016;16(1):315.
77. Kwok TC, Bai X, Li JC, et al. Effectiveness of cognitive training in Chinese older people with subjective cognitive complaints: A randomized placebo-controlled trial. *International journal of geriatric psychiatry* 2013;28(2):208-15.
78. Lam LC, Chau RC, Wong BM, et al. A 1-year randomized controlled trial comparing mind body exercise (Tai Chi) with stretching and toning exercise on cognitive function in older Chinese adults at risk of cognitive decline. *Journal of the American Medical Directors Association* 2012;13(6):568. e15-68. e20.
79. Lam LC-w, Chan WC, Leung T, et al. Would older adults with mild cognitive impairment adhere to and benefit from a structured lifestyle activity intervention to enhance cognition?: a cluster randomized controlled trial. *PLoS One* 2015;10(3):e0118173.
80. Langoni CdS, Resende TdL, Barcellos AB, et al. The effect of group exercises on balance, mobility, and depressive symptoms in older adults with mild cognitive impairment: a randomized controlled trial. *Clinical rehabilitation* 2019;33(3):439-49.
81. Law LL, Barnett F, Yau MK, et al. Effects of functional tasks exercise on older adults with cognitive impairment at risk of Alzheimer's disease: a randomised controlled trial. *Age and ageing* 2014;43(6):813-20.
82. Lazarou I, Parastatidis T, Tsolaki A, et al. International ballroom dancing against neurodegeneration: a randomized controlled trial in Greek community-dwelling elders with mild cognitive impairment. *American Journal of Alzheimer's Disease & Other Dementias®* 2017;32(8):489-99.
83. Li B-Y, He N-Y, Qiao Y, et al. Computerized cognitive training for Chinese mild cognitive impairment patients: A neuropsychological and fMRI study. *NeuroImage: Clinical* 2019;22:101691.
84. Lim H-W, Saw W-Y, Feng L, et al. Dataset on gene expression in the elderly after Mindfulness Awareness Practice or Health Education Program. *Data in brief* 2018;18:902-12.
85. Luijpen MW, Swaab DF, Sergeant JA, et al. Effects of transcutaneous electrical nerve stimulation (TENS) on memory in elderly with mild cognitive impairment. *Behavioural brain research* 2005;158(2):349-57.
86. Maffei L, Picano E, Andreassi M, et al. Randomized trial on the effects of a combined physical/cognitive training in aged MCI subjects: the Train the Brain study. *Scientific reports* 2017;7:39471.

For peer review only - http://bmjopen.bmj.com/site/about/guidelines.xhtml

87. İnel Manav A, Simsek N. The Effect of Reminiscence Therapy With Internet-Based Videos on Cognitive Status and Apathy of Older People With Mild Dementia. *Journal of geriatric psychiatry and neurology* 2019;32(2):104-13.
88. Melendez JC, Torres M, Redondo R, et al. Effectiveness of follow-up reminiscence therapy on autobiographical memory in pathological ageing. *International Journal of Psychology* 2017;52(4):283-90.
89. Nagamatsu LS, Handy TC, Hsu CL, et al. Resistance training promotes cognitive and functional brain plasticity in seniors with probable mild cognitive impairment. *Archives of internal medicine* 2012;172(8):666-68.
90. Olsen C, Pedersen I, Bergland A, et al. Effect of animal-assisted interventions on depression, agitation and quality of life in nursing home residents suffering from cognitive impairment or dementia: a cluster randomized controlled trial. *International journal of geriatric psychiatry* 2016;31(12):1312-21.
91. Pantoni L, Poggesi A, Diciotti S, et al. Effect of attention training in mild cognitive impairment patients with subcortical vascular changes: the RehAtt Study. *Journal of Alzheimer's Disease* 2017;60(2):615-24.
92. Park J-H, Park J-H. Does cognition-specific computer training have better clinical outcomes than non-specific computer training? A single-blind, randomized controlled trial. *Clinical rehabilitation* 2018;32(2):213-22.
93. Poinsatte K, Smith EE, Torres VO, et al. T and B cell subsets differentially correlate with amyloid deposition and neurocognitive function in patients with amnestic mild cognitive impairment after one year of physical activity. *Exercise immunology review* 2019;25
94. Pongan E, Tillmann B, Leveque Y, et al. Can musical or painting interventions improve chronic pain, mood, quality of life, and cognition in patients with mild Alzheimer's disease? Evidence from a randomized controlled trial. *Journal of Alzheimer's Disease* 2017;60(2):663-77.
95. Poptsi E, Lazarou I, Markou N, et al. A Comparative Single-Blind Randomized Controlled Trial With Language Training in People With Mild Cognitive Impairment. *American Journal of Alzheimer's Disease & Other Dementias®* 2019;34(3):176-87.
96. Qi M, Zhu Y, Zhang L, et al. The effect of aerobic dance intervention on brain spontaneous activity in older adults with mild cognitive impairment: A resting-state functional MRI study. *Experimental and therapeutic medicine* 2019;17(1):715-22.
97. Rapp S, Brenes G, Marsh A. Memory enhancement training for older adults with mild cognitive impairment: a preliminary study. *Aging & Mental Health* 2002;6(1):5-11.
98. Rojas GJ, Villar V, Iturry M, et al. Efficacy of a cognitive intervention program in patients with mild cognitive impairment. *International Psychogeriatrics* 2013;25(5):825-31.
99. Rozzini L, Costardi D, Chilovi BV, et al. Efficacy of cognitive rehabilitation in patients with mild cognitive impairment treated with cholinesterase inhibitors. *International Journal of Geriatric Psychiatry: A journal of the psychiatry of late life and allied sciences* 2007;22(4):356-60.
100. Savulich G, Piercy T, Fox C, et al. Cognitive training using a novel memory game on an iPad in patients with amnestic mild cognitive impairment (aMCI). *International Journal of Neuropsychopharmacology* 2017;20(8):624-33.
101. Scherder EJ, Van Paasschen J, Deijen J-B, et al. Physical activity and executive functions in the elderly with mild cognitive impairment. *Aging & mental health* 2005;9(3):272-80.
102. Shimada H, Makizako H, Doi T, et al. Effects of combined physical and cognitive exercises on cognition and mobility in patients with mild cognitive impairment: a randomized clinical trial. *Journal of the American Medical Directors Association* 2018;19(7):584-91.
103. Shimizu N, Umemura T, Matsunaga M, et al. Effects of movement music therapy with a percussion instrument on physical and frontal lobe function in older adults with mild cognitive impairment: A randomized controlled trial. *Aging & mental health* 2018;22(12):1614-26.

For peer review only - http://bmjopen.bmj.com/site/about/guidelines.xhtml

104. Simon SS, Hampstead BM, P Nucci M, et al. Cognitive and brain activity changes after mnemonic strategy training in amnestic mild cognitive impairment: evidence from a randomized controlled trial. *Frontiers in aging neuroscience* 2018;10:342.
105. Song D, Doris S. Effects of a moderate-intensity aerobic exercise programme on the cognitive function and quality of life of community-dwelling elderly people with mild cognitive impairment: A randomised controlled trial. *International journal of nursing studies* 2019;93:97-105.
106. Suzuki T, Shimada H, Makizako H, et al. Effects of multicomponent exercise on cognitive function in older adults with amnestic mild cognitive impairment: a randomized controlled trial. *BMC neurology* 2012;12(1):128.
107. Troyer AK, Murphy KJ, Anderson ND, et al. Changing everyday memory behaviour in amnestic mild cognitive impairment: a randomised controlled trial. *Neuropsychological rehabilitation* 2008;18(1):65-88.
108. Tsai C-L, Ukropec J, Ukropcová B, et al. An acute bout of aerobic or strength exercise specifically modifies circulating exerkine levels and neurocognitive functions in elderly individuals with mild cognitive impairment. *NeuroImage: Clinical* 2018;17:272-84.
109. Tsantali E, Economidis D, Rigopoulou S. Testing the benefits of cognitive training vs. cognitive stimulation in mild Alzheimer's disease: a randomised controlled trial. *Brain Impairment* 2017;18(2):188-96.
110. Van Uffelen JG, Paw MJCA, Hopman-Rock M, et al. The effect of walking and vitamin B supplementation on quality of life in community-dwelling adults with mild cognitive impairment: a randomized, controlled trial. *Quality of Life Research* 2007;16(7):1137-46.
111. Wei X-h, Ji L-l. Effect of handball training on cognitive ability in elderly with mild cognitive impairment. *Neuroscience letters* 2014;566:98-101.
112. Yang H, Leaver AM, Siddarth P, et al. Neurochemical and neuroanatomical plasticity following memory training and yoga interventions in older adults with mild cognitive impairment. *Frontiers in aging neuroscience* 2016;8:277.
113. Yoon DH, Kang D, Kim Hj, et al. Effect of elastic band-based high-speed power training on cognitive function, physical performance and muscle strength in older women with mild cognitive impairment. *Geriatrics & gerontology international* 2017;17(5):765-72.
114. Young DK, Kwok TC, Ng PY. A single blind randomized control trial on support groups for Chinese persons with mild dementia. *Clinical interventions in aging* 2014;9:2105.
115. Young K-w, Ng P, Kwok T, et al. The effects of holistic health group interventions on improving the cognitive ability of persons with mild cognitive impairment: a randomized controlled trial. *Clinical interventions in aging* 2017;12:1543.
116. Yun K, Song I-U, Chung Y-A. Changes in cerebral glucose metabolism after 3 weeks of noninvasive electrical stimulation of mild cognitive impairment patients. *Alzheimer's research & therapy* 2016;8(1):49.
117. Zhao J, Li H, Lin R, et al. effects of creative expression therapy for older adults with mild cognitive impairment at risk of Alzheimer’s disease: a randomized controlled clinical trial. *Clinical interventions in aging* 2018;13:1313.
118. Zhu Y, Wu H, Qi M, et al. Effects of a specially designed aerobic dance routine on mild cognitive impairment. *Clinical interventions in aging* 2018;13:1691.

For peer review only

For peer review only - http://bmjopen.bmj.com/site/about/guidelines.xhtml

Table 1. Included Studies

Lead Author	Year	Country	Diagnosis	Number of Groups	Group 1	Group 2	Group 3	Group 4	Group 5	Number of measures
Amjad [37]	2019	Pakistan	MCI	2	Aerobic Exercise	Non-Aerobic Exercise	-	-	-	4
Bae [38]	2019	Japan	MCI	2	Multi-Intervention Programme	Active Control	-	-	-	10
Baker [39]	2010	USA	MCI	2	Aerobic Exercise	Stretching	-	-	-	11
Belleville [40]	2018	Canada	MCI	3	Cognitive Training	Psychosocial Intervention	Control	-	-	7
Biasutti [41]	2017	Italy	MCI	2	Cognitive Training	Gym Activities	-	-	-	4
Bono [42]	2015	Italy	MCI	2	Animal Assisted Therapy	Control	-	-	-	4
Burgio [43]	2018	Italy	MCI	2	Numerical Training	Executive Training	-	-	-	13
Buschert [44]	2012	Germany	MCI	2	Cognitive Training	Active Control	-	-	-	5
Carretti [45]	2013	Italy	MCI	2	Cognitive Training	Active Control	-	-	-	16
Cavallo [46]	2016	Italy	Dementia	2	Cognitive Training	Active Control	-	-	-	3
Chan [47]	2016	Hong Kong	MCI	2	Chinese Calligraphy	Computer Activities	-	-	-	13
Chan [48]	2017	Hong Kong	MCI	2	Chinese Calligraphy	Computer Activities	-	-	-	8
Choi [49]	2018	South Korea	MCI	2	Ground Kayaking	Home Exercise Education	-	-	-	7
Combourieu Donnezan [50]	2018	France	MCI	4	Physical Training	Cognitive Training	Simultaneous Cognitive and Physical Training	Control	-	4
DiNapoli [51]	2016	USA	MCI	2	Individualised Social Activities	Control	-	-	-	4
Doi [52]	2013	Japan	MCI	2	Exercise	Active Control	-	-	-	4
Doi [53]	2017	Japan	MCI	3	Dance	Playing Musical Instruments	Health Education Group	-	-	4
Drumond Marra [54]	2015	Brazil	MCI	2	TMS	Sham TMS	-	-	-	6
Emsaki [55]	2017	Iran	MCI	2	Cognitive Training	Active Control	-	-	-	9
Eyre [56]	2017	USA	MCI	2	Yoga	Cognitive Training	-	-	-	10
Feng [57]	2018	China	MCI	2	Single Component Cognitive Training	Multiple Component Cognitive Training	-	-	-	3
Fernandez-Calvo [58]	2015	Spain	Dementia	2	Multi-Intervention Programme	Control	-	-	-	21
Fiatarone Singh [59]	2014	Australia	MCI	4	Progressive resistance training and sham cognitive training	Progressive resistance training and cognitive training	Cognitive training	Control	-	12

For peer review only - http://bmjopen.bmj.com/site/about/guidelines.xhtml

For peer review only

Finn [60]	2015	Australia	MCI	2	Repetition-lag Training	Control	-	-	-	6
Fogarty [61]	2016	Canada	MCI	2	Memory Intervention Program and Tai Chi	Memory Intervention Program	-	-	-	5
Forster [62]	2011	Germany	Both	2	Cognitive Training	Control	-	-	-	10
Galante [63]	2007	Italy	Dementia	2	Cognitive Training	Active Control	-	-	-	12
Greenaway [21]	2013	USA	MCI	2	Memory Support System (Memory Rehabilitation) with Training	Memory Support System without Training	-	-	-	15
Hagovska [64]	2017	Czech Republic	MCI	2	Cognitive Training (Computer Based)	Cognitive Training	-	-	-	0
Hagovska [65]	2016	Czech Republic	MCI	2	Cognitive Training and Dynamic Balance Training	Balance Training	-	-	-	4
Han [66]	2017	South Korea	MCI	2	Ubiquitous Spaced Retrieval-based Memory Advancement and Rehabilitation Training	Control	-	-	-	4
Han [67]	2017	South Korea	Both	2	Multimodal Cognitive Enhancement Therapy	Active Control	-	-	-	7
Hattori [29]	2011	Japan	Dementia	2	Art Therapy	Active Control	-	-	-	4
Ho [68]	2018	Hong Kong	Both	3	Dance Movement Therapy	Physical Exercise	Control	-	-	7
Horie [69]	2016	Brazil	MCI	2	Group Weight Loss Programme	Control	-	-	-	10
Hyer [70]	2016	USA	MCI	2	Cognitive Training (Computer Based)	Active Control	-	-	-	3
Jansen [22]	2011	The Netherlands	Dementia	2	Case Management	Control	-	-	-	5
Jean [71]	2010	Canada	MCI	2	Cognitive Training	Active Control	-	-	-	10
Jelcic [72]	2012	Italy	Dementia	2	Lexical-Semantic Treatment	Cognitive Stimulation	-	-	-	11
Jeong [73]	2016	South Korea	MCI	2	Cognitive Intervention (Group based)	Cognitive Intervention (Home Based)	-	-	-	8
Kinsella [23]	2009	Australia	MCI	2	Cognitive Intervention	Control	-	-	-	4
Kohanpour [74]	2017	Iran	MCI	4	Aerobic Exercise	Lavender Extract	Aerobic Exercise and Lavender Extract	Control	-	14
Koivisto [24]	2016	Finland	Dementia	2	Psychosocial Intervention	Control	-	-	-	7
Kovacs [75]	2013	Hungary	MCI	2	Multimodal Exercise	Control	-	-	-	1
Kuster [76]	2016	Germany	MCI	3	Cognitive Training	Physical Training	Control	-	-	7
Kwok [77]	2012	Hong Kong	MCI	2	Cognitive Training	Active Control	-	-	-	5

For peer review only - http://bmjopen.bmj.com/site/about/guidelines.xhtml

Lam [78]	2012	Hong Kong	MCI	2	Tai Chi	Stretching	-	-	-	4
Lam [79]	2015	Hong Kong	MCI	4	Cognitive Training	Cognitive and Physical Training	Physical Training	Social Groups	-	2
Lam [25]	2010	Hong Kong	Dementia	2	Case Management	Control	-	-	-	2
Langoni [80]	2019	Brazil	MCI	2	Group Exercise	Control	-	-	-	14
Law [81]	2014	Hong Kong	MCI	2	Functional Tasks Exercise Programme	Cognitive Training	-	-	-	7
Lazarou [82]	2017	Greece	MCI	2	Ballroom Dancing	Control	-	-	-	5
Li [83]	2019	China	MCI	2	Computerised Cognitive Training	Control	-	-	-	4
Lim [84]	2018	Singapore	MCI	2	Mindfulness	Health Education	-	-	-	5
Logsdon [26]	2010	USA	Dementia	2	Early Stage Memory Loss Support Group	Control	-	-	-	10
Luijpen [85]	2005	The Netherlands	MCI	2	TENs	Sham TENS	-	-	-	6
Maffei [86]	2017	Italy	MCI	2	Multidomain Training	Control	-	-	-	10
Manav [87]	2019	Turkey	Dementia	2	Reminiscence Therapy	Social Interview	-	-	-	6
Melendez [88]	2015	Spain	Both	2	Reminiscence Therapy	Control	-	-	-	6
Nagamatsu [89]	2012	Canada	MCI	2	Aerobic Exercise	Resistance Training	-	-	-	13
Olsen [90]	2016	Norway	Both	2	Animal Assisted Therapy	Control	-	-	-	9
Pantoni [91]	2017	Italy	MCI	2	Attention Process Training	Control	-	-	-	4
Park [92]	2018	South Korea	MCI	2	Cognition specific computer training	Non-specific computer training	-	-	-	5
Poinsatte [93]	2019	USA	MCI	2	Aerobic Exercise	Stretching	-	-	-	3
Pongan [94]	2017	France	Dementia	2	Choral Singing	Painting	-	-	-	14
Poptsi [95]	2018	Greece	MCI	5	Paper Language Tasks	Computer Language Tasks	Oral Language Tasks	Active Control	Control	4
Qi [96]	2019	China	MCI	2	Aerobic Exercise	Control	-	-	-	3
Rapp [97]	2002	USA	MCI	2	Memory Enhancement Training (Multi-Component)	Control	-	-	-	9
Rojas [98]	2013	Argentina	MCI	2	Cognitive Intervention	Control	-	-	-	8
Rozzini [99]	2007	Italy	MCI	2	Cognitive Training and AChEIs	AChEIs	-	-	-	7
Savulich [100]	2017	UK	MCI	2	Cognitive Training	Control	-	-	-	9

For peer review only - http://bmjopen.bmj.com/site/about/guidelines.xhtml

Scherder [101]	2010	The Netherlands	MCI	3	Walking	Hand and Face Exercises	Control	-	-	11
Shimada [102]	2017	Japan	MCI	2	Physical and Cognitive Training	Health Education Group	-	-	-	7
Shimizu [103]	2017	Japan	MCI	2	Movement Music Therapy	Single Training Task	-	-	-	4
Simon [104]	2018	Brazil	MCI	2	Memory Training	Active Control	-	-	-	8
Song [105]	2019	China	MCI	2	Aerobic Exercise	Active Control	-	-	-	4
Suzuki [106]	2012	Japan	MCI	2	Multicomponent Exercise Group	Active Control	-	-	-	6
Tappen [27]	2014	USA	Both	2	Cognitive Training (Home Based)	Life Story Interview	-	-	-	11
Troyer [107]	2008	Canada	MCI	2	Multicomponent Intervention	Control	-	-	-	6
Tsai [108]	2018	Taiwan	MCI	3	Aerobic Exercise	Resistance Training	Control	-	-	7
Tsantali [109]	2017	Greece	Dementia	3	Cognitive Training	Cognitive Stimulation	Control	-	-	5
van Uffelen [110]	2007	The Netherlands	MCI	4	Walking	Placebo Activity	Folic Acid/ Vitamin B Supplements	Placebo Pills	-	3
Waldorff [28]	2012	Denmark	Dementia	2	Multifaceted Counselling, Education and Support	Control	-	-	-	2
Wei [111]	2014	China	MCI	2	Handball Training	Control	-	-	-	8
Yang [112]	2016	USA	MCI	2	Memory Enhancement Training	Yoga	-	-	-	3
Yoon [113]	2017	South Korea	MCI	2	High-Speed Power Strength Training	Low-Speed Strength Training	-	-	-	5
Young [114]	2014	Hong Kong	Dementia	2	Support Groups	Control	-	-	-	4
Young [115]	2017	Hong Kong	MCI	2	Holistic Health Group	Control	-	-	-	4
Yun [116]	2016	South Korea	MCI	2	TDS	Sham TDS	-	-	-	1
Zhao [117]	2018	China	MCI	2	Creative Expression Therapy	Cognitive Training	-	-	-	7
Zhu [118]	2018	China	MCI	2	Dance	Control	-	-	-	7

For peer review only - http://bmjopen.bmj.com/site/about/guidelines.xhtml

Table 2. Outcome measures by domain and subdomains

Person living with dementia measures<br>Domain and subdomain	Outcome Measure	N
**Cognition/Memory**		**219**
Cognition	MMSE	37
	Trail Making Test	27
	Digit Span Test	12
	ADAS-Cog	10
	Rey Auditory Test	9
	Rivermead Behavioural Memory Test	9
	Stroop Test	7
	MMQ	7
	Novelli Lexical Test	7
	MoCA	6
	CDR	6
	Verbal Fluency	6
	CERAD-NB	5
	Addenbrooke's Cognitive Examination	4
	Boston Naming Test	4
	Rey Osterrieth Complex Figure Task	4
	Montreal Cognitive Test	3
	Attentional Matrices Test	3
	California Verbal Learning Test	3
	Digit Symbol Coding Test	3
	Hopkins Verbal Learning Test	3
	The Wechsler Memory Scale	3
	CAMcog	2
	Cognitive Failures Test	2
	Color Trails Test	2
	Dementia Rating Scale-2	2
	DSM IV Test	2
	Auditory Verbal Learning Test	2
	Corsi's Block Tapping Test	2
	Frontal Assessment Test	2
	Fuld Object Memory Evaluation	2
	Logical Memory (Subtest of Wechsler Memory Scale)	2
	Prospective and Retrospective Memory Questionnaire	2
	Pyramids & Palm Trees	2
	Questionnaire d'Auto Evaluation de la Memoire	2
	Raven's Coloured Matrices	2
	Repeatable Battery Test	2
	The verbal learning and memory test	2
	Visual Memory Span	2
	Wechsler Adult Intelligence Scale	2
Knowledge of Memory Strategies	Memory Strategy Toolbox	2
	Strategy Knowledge Repertoire	1
Attention	Test of Everyday Attention	2
**Behavioural and Psychological Symptoms of dementia**		**51**
Anxiety/Depression	Geriatric Depression Scale*	21
	Cornell Scale for Depression in Dementia*	7

For peer review only - http://bmjopen.bmj.com/site/about/guidelines.xhtml

	Hospital Anxiety and Depression Scale	4
	Beck Depression Inventory	1
Other	Neuropsychiatric Inventory*	12
	Apathy Evaluation Scale	3
	Revised Memory and behaviour problem checklist*	
**Everyday Living**		**20**
Activities of daily Living	Instrumental Activities of Daily Living*	8
	Bayer Activities of Daily Living Scale	3
	Alzheimer's Disease Cooperative Study Activities of Daily Living Scale	2
	Barthel index	2
Functional Ability	Functional Activities Questionnaire	3
	Functional and Cognitive Assessment Test and Functional Rating Scale for Dementia	2
**Physical Outcomes**		**19**
Physical Performance	Timed Up and Go Test	7
	Gait	3
	Handgrip strength	3
	Stride	2
	Walking Speed	2
Physical Measures	Weight	2
**Quality of Life/Wellbeing**		**15**
Quality of Life	QoL in Alzheimer's Disease*	7
	Dementia Quality of Life Instrument*	3
	EuroQoL EQ 5D*	2
	EQ-VAS	1
Stress	Perceived Stress Scale	1
General Wellbeing	SF-36	1
**Biological Outcome**		**9**
Brain Activity	EEG	4
	MRI	2
Biomarker	BDNF	3
**Adherence to Intervention**		**2**
Adherence to intervention	Adherence	2

Caregiver Measures

**Domain**	**Outcome Measure**	**N**
**Depression**		**5**
	The Center for Epidemiological Studies Depression Scale*	3
	Geriatric Depression Scale	1
	Beck Depression Inventory	1
**Caregiver Burden**		**2**
	Zarit caregiver burden interview*	2
**General Wellbeing**		**1**
	Short Form Health Survey (SF-36)*	1
**Knowledge of Memory Strategies**		**1**
	Strategy Knowledge Repertoire	1
**Quality of Life**		**1**
	EQ-VAS	1

For peer review only - http://bmjopen.bmj.com/site/about/guidelines.xhtml

**Stress**		**1**
	Perceived Stress Scale	1

*Measure recommended by INTERDEM Consensus [14]

For peer review only

Table 3. Outcome measure domain by diagnosis and intervention

	Number of Studies	BPSD	Biological Outcome	Caregiver Measure	Cognition/ Memory	Everyday Living	Physical Measures	Physical Performance	Quality of Life/ Wellbeing	Task Performance
**Diagnosis**										
Both	6	5	-	1	12	1	-	-	-	-
Dementia	14	16	-	7	42	6	-	-	6	-
MCI	71	30	9	3	163	12	2	17	9	2
**Type of Intervention**										
Animal Assisted Therapy	2	2	-	-	2	1	-	-	-	-
Art-Based Therapy	2	1	-	1	6	1	-	-	-	-
Case Management	2	2	-	3	1	-	-	-	1	-
Chinese Calligraphy	2	1	1	-	4	-	-	-	-	-
Cognitive Training	37	23	2	3	103	11	-	1	6	2
Cognitive Training and Physical Activity	4	-	-	-	14	2	-	2	-	-
Multicomponent Psychosocial Intervention	4	6	-	3	10	2	-	2	3	-
Music Based Intervention	2	1	-	-	7	-	1	2	1	-
Physical Activity	25	11	6	-	53	3	1	10	2	-
Reminiscence Therapy	2	1	-	-	2	-	-	-	-	-
Support Group	3	3	-	1	1	-	-	-	1	-

For peer review only - http://bmjopen.bmj.com/site/about/guidelines.xhtml

Table 4. Outcome measure domain by country

Country	Number of studies	BPSD	Biological Outcome	Caregiver Measure	Cognition/Memory	Functional ability	Physical Measures	Physical Performance	Quality of Life/ Wellbeing	Task Performance
**Argentina**	1	1	0	0	6	1	0	0	0	0
**Australia**	4	0	0	1	5	1	0	0	0	0
**Brazil**	5	1	1	0	14	0	0	1	0	0
**Canada**	6	2	0	0	16	0	0	2	0	0
**Mainland China, Hong Kong and Taiwan**	20	10	5	1	35	2	0	0	0	1
**Czech Republic**	3	0	0	0	3	2	0	1	0	0
**Denmark**	1	2	0	2	1	1	0	0	2	0
**Finland**	1	1	0	1	3	1	0	0	1	0
**France**	3	1	0	0	6	0	0	2	1	0
**Germany**	4	1	0	0	10	0	0	0	1	0
**Greece**	4	3	0	0	18	2	0	0	1	0
**Hungary**	1	0	0	0	0	0	0	1	0	0
**Iran**	3	1	1	0	3	0	1	0	0	0
**Italy**	11	8	0	0	32	6	0	0	1	0
**Japan**	8	2	0	1	16	1	1	6	0	0
**Norway**	1	1	0	0	1	0	0	0	0	0
**Pakistan**	1	0	1	0	3	0	0	0	0	0
**Singapore**	1	0	0	0	0	0	0	0	0	0
**South Korea**	8	5	0	0	14	1	0	4	3	0
**Spain**	3	2	0	0	2	0	0	0	0	0
**The Netherlands**	5	0	0	2	10	0	0	0	2	0
**Turkey**	1	1	0	0	1	0	0	0	0	0
**UK**	1	3	0	0	1	0	0	0	0	0
**USA**	10	6	1	3	19	2	0	0	3	1

For peer review only - http://bmjopen.bmj.com/site/about/guidelines.xhtml

Figure 1. Flow Chart of Included Studies

Figure 2. Trends in outcome measures over time

Note: QoL = quality of life; BPSD = behavioural and psychological symptoms of dementia

For peer review only

For peer review only - http://bmjopen.bmj.com/site/about/guidelines.xhtml

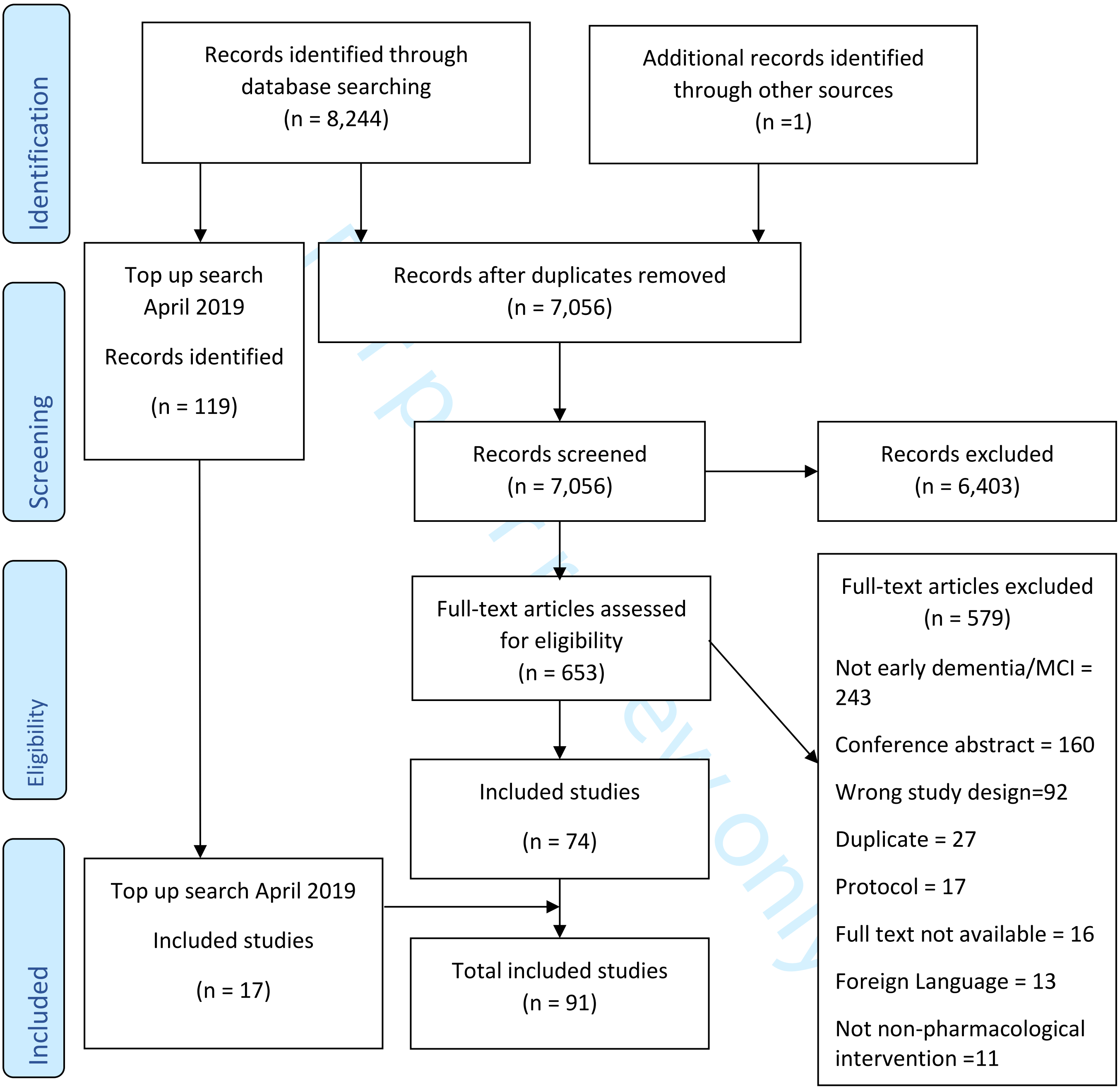
Identification
Screening
Eligibility
Included
Records identified through database searching (n = 8,244)
Additional records identified through other sources (n =1)
Top up search April 2019
Records identified
(n = 119)
Records after duplicates removed (n = 7,056)
Records screened (n = 7,056)
Records excluded (n = 6,403)
Full-text articles assessed for eligibility (n = 653)
Full-text articles excluded (n = 579)
Not early dementia/MCI = 243
Conference abstract = 160
Wrong study design=92
Duplicate = 27
Protocol = 17
Full text not available = 16
Foreign Language = 13
Not non-pharmacological intervention =11
Included studies
(n = 74)
Top up search April 2019
Included studies
(n = 17)
Total included studies (n = 91)

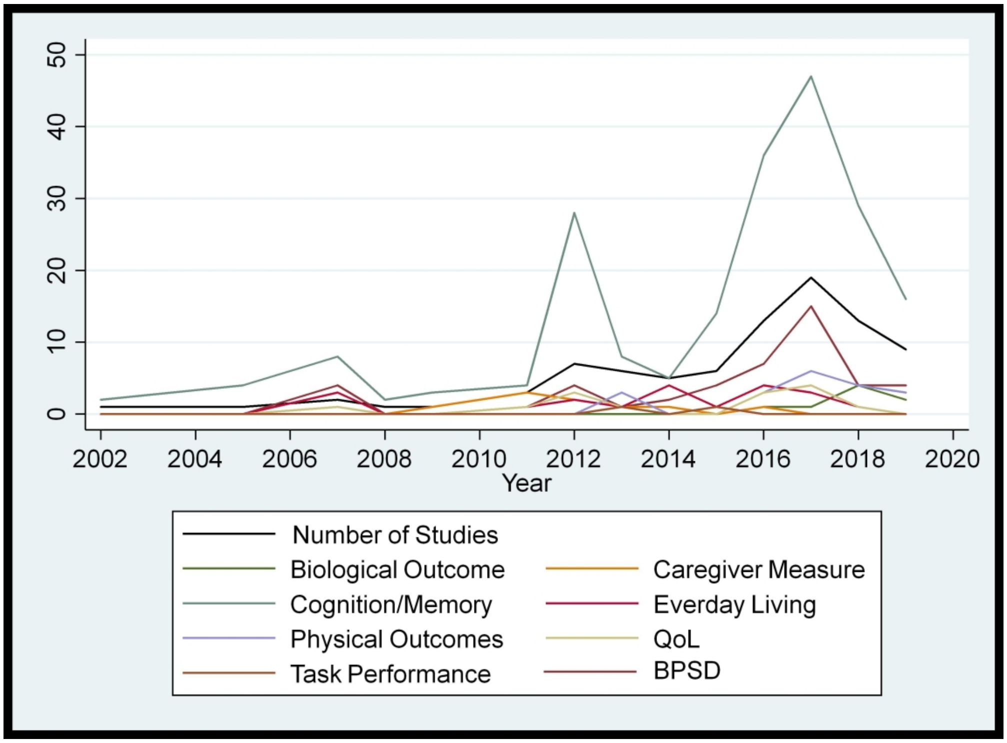

Figure 2. outcome measures over time
Note: QoL = quality of life; BPSD = behavioural and psychological symptoms of dementia.

887x651mm (96 x 96 DPI)

Supplementary Table 1. Search Strategy for OVID

	Search term		Search term continued
1	Early dementia	39	self help group
2	Mild dementia	40	psychotherapy
3	mild alzheimer*	41	CBT
4	early alzheimer*	42	Cognitive behavio?ral therap*
5	cognitive impairment	43	Cognitive behavioural therap*
6	age related cognitive impairment	44	Talking therap*
7	Mild cognitive impairment	45	Individual therap*
8	MCI	46	Peer support
9	mild neurocognitive disorder	47	Counselling
**10**	**1 OR 2 OR 3 OR 4 OR 5 OR 6 OR 7 OR 8 OR 9**	48	Communication
11	cognitive training	49	acupuncture therap*
12	brain training	50	acupuncture
13	memory training	51	acupuncture points
14	Behavio?r therap*	52	Transcranial Magnetic Stimulation
15	Behavio?r modification	53	TMS
16	pleasant activit*	54	Relaxation therap*
17	Cognitive stimulation therapy	55	Therap* relaxation
18	CST	56	Relaxation techniques
19	Transcutaneous Electrical Nerve Stimulation	57	Early intervention
20	TENS	58	Alternative therap*
21	Exercise	**59**	**11 OR 12 OR 13 OR 14 OR 15 OR 16 OR 17 OR 18 OR 19 OR 20 OR 21 OR 22 OR 23 OR 24 OR 25 OR 26 OR 27 OR 28 OR 29 OR 30 OR 31 OR 32 OR 33 OR 34 OR 35 OR 36 OR 37 OR 38 OR 39 OR 40 OR 41 OR 42 OR 43 OR 44 OR 45 OR 46 OR 47 OR 48 OR 49 OR 50 OR 51 OR 52 OR 53 OR 54 OR 55 OR 56 OR 57 OR 58 OR 59**
22	exercise therap*	60	randomized controlled trial
23	Walking	61	randomised controlled trial

For peer review only

**24**	music therap*	**62**	RCT
**15**	reminiscence therap*	**63**	Clinical Trial
**26**	massage therap*	**64**	intervention
**27**	therap* touch	**65**	**60 OR 61 OR 62 OR 63 OR 64 OR 65**
**28**	recreation therap*	**66**	early dementia
**29**	light therap*	**67**	mild dementia
**30**	therap* light	**68**	mild alzheimer*
**31**	sensory stimulation	**69**	early alzheimer*
**32**	multisensory stimulation	**70**	cognitive impairment
**33**	complementary therap*	**71**	age related cognitive impairment
**34**	aromatherapy	**72**	Mild cognitive impairment
**35**	support group	**73**	MCI
**36**	therap* group	**74**	mild neurocognitive disorder
**37**	memory group	**75**	**66 OR 67 OR 68 OR 69 OR 70 OR 71 OR 72 OR 73 OR 74 OR 75**
**38**	self help	**76**	**10 AND 59 AND 75**

For peer review only

Preferred Reporting Items for Systematic reviews and Meta-Analyses extension for Scoping Reviews (PRISMA-ScR) Checklist

SECTION	ITEM	PRISMA-ScR CHECKLIST ITEM	REPORTED ON PAGE #
**TITLE**			
Title	1	Identify the report as a scoping review.	1
**ABSTRACT**			
Structured summary	2	Provide a structured summary that includes (as applicable): background, objectives, eligibility criteria, sources of evidence, charting methods, results, and conclusions that relate to the review questions and objectives.	2
**INTRODUCTION**			
Rationale	3	Describe the rationale for the review in the context of what is already known. Explain why the review questions/objectives lend themselves to a scoping review approach.	5-6
Objectives	4	Provide an explicit statement of the questions and objectives being addressed with reference to their key elements (e.g., population or participants, concepts, and context) or other relevant key elements used to conceptualize the review questions and/or objectives.	6
**METHODS**			
Protocol and registration	5	Indicate whether a review protocol exists; state if and where it can be accessed (e.g., a Web address); and if available, provide registration information, including the registration number.	6
Eligibility criteria	6	Specify characteristics of the sources of evidence used as eligibility criteria (e.g., years considered, language, and publication status), and provide a rationale.	7-8
Information sources*	7	Describe all information sources in the search (e.g., databases with dates of coverage and contact with authors to identify additional sources), as well as the date the most recent search was executed.	8
Search	8	Present the full electronic search strategy for at least 1 database, including any limits used, such that it could be repeated.	Table 1
Selection of sources of evidence†	9	State the process for selecting sources of evidence (i.e., screening and eligibility) included in the scoping review.	8-9
Data charting process‡	10	Describe the methods of charting data from the included sources of evidence (e.g., calibrated forms or forms that have been tested by the team before their use, and whether data charting was done independently or in duplicate) and any processes for obtaining and confirming data from investigators.	9
Data items	11	List and define all variables for which data were sought and any assumptions and simplifications made.	9
Critical appraisal of individual sources of evidence§	12	If done, provide a rationale for conducting a critical appraisal of included sources of evidence; describe the methods used and how this information was used in any data synthesis (if appropriate).	N/A
Synthesis of results	13	Describe the methods of handling and summarizing the data that were charted.	9

For peer review only - http://bmjopen.bmj.com/site/about/guidelines.xhtml

SECTION	ITEM	PRISMA-ScR CHECKLIST ITEM	REPORTED ON PAGE #
**RESULTS**			
Selection of sources of evidence	14	Give numbers of sources of evidence screened, assessed for eligibility, and included in the review, with reasons for exclusions at each stage, ideally using a flow diagram.	9-10 and Figure 1
Characteristics of sources of evidence	15	For each source of evidence, present characteristics for which data were charted and provide the citations.	10 and Table 2
Critical appraisal within sources of evidence	16	If done, present data on critical appraisal of included sources of evidence (see item 12).	N/A
Results of individual sources of evidence	17	For each included source of evidence, present the relevant data that were charted that relate to the review questions and objectives.	Not feasible
Synthesis of results	18	Summarize and/or present the charting results as they relate to the review questions and objectives.	10-13
**DISCUSSION**			
Summary of evidence	19	Summarize the main results (including an overview of concepts, themes, and types of evidence available), link to the review questions and objectives, and consider the relevance to key groups.	13-14
Limitations	20	Discuss the limitations of the scoping review process.	16-17
Conclusions	21	Provide a general interpretation of the results with respect to the review questions and objectives, as well as potential implications and/or next steps.	17
**FUNDING**			
Funding	22	Describe sources of funding for the included sources of evidence, as well as sources of funding for the scoping review. Describe the role of the funders of the scoping review.	18

JBI = Joanna Briggs Institute; PRISMA-ScR = Preferred Reporting Items for Systematic reviews and Meta-Analyses extension for Scoping Reviews.

* Where *sources of evidence* (see second footnote) are compiled from, such as bibliographic databases, social media platforms, and Web sites.

† A more inclusive/heterogeneous term used to account for the different types of evidence or data sources (e.g., quantitative and/or qualitative research, expert opinion, and policy documents) that may be eligible in a scoping review as opposed to only studies. This is not to be confused with *information sources* (see first footnote).

‡ The frameworks by Arksey and O'Malley (6) and Levac and colleagues (7) and the JBI guidance (4, 5) refer to the process of data extraction in a scoping review as data charting.

§ The process of systematically examining research evidence to assess its validity, results, and relevance before using it to inform a decision. This term is used for items 12 and 19 instead of "risk of bias" (which is more applicable to systematic reviews of interventions) to include and acknowledge the various sources of evidence that may be used in a scoping review (e.g., quantitative and/or qualitative research, expert opinion, and policy document).

From: Tricco AC, Lillie E, Zarin W, O'Brien KK, Colquhoun H, Levac D, et al. PRISMA Extension for Scoping Reviews (PRISMA-ScR): Checklist and Explanation. Ann Intern Med. ;169:467–473. doi: 10.7326/M18-0850

For peer review only
